# Tuning chiroptical properties and device hyperfluorescence efficiency *via* acceptor modification in an intramolecularly sensitised TADF dendrimer

**DOI:** 10.1039/d6sc04906k

**Published:** 2026-09-03

**Authors:** Mahni Fatahi, Yan Xu, Dongyang Chen, Praveen Choudhary, Jhon Sebastian Oviedo Ortiz, Jasmin Seibert, Stefan Bräse, Jeanne Crassous, Xiao-Hong Zhang, Eli Zysman-Colman

**Affiliations:** a Organic Semiconductor Centre, EaStCHEM School of Chemistry, University of St Andrews St Andrews KY16 9ST UK eli.zysman-colman@st-andrews.ac.uk; b Institute of Functional Nano & Soft Materials (FUNSOM), Joint International Research Laboratory of Carbon-Based Functional Materials and Devices, Soochow University Suzhou Jiangsu 21523 P. R. China xiaohong_zhang@suda.edu.cn; c University of Rennes, CNRS, ISCR (Institut des Sciences Chimiques de Rennes) – UMR 6226 F-35000 Rennes France jeanne.crassous@univ-rennes.fr; d Institute of Organic Chemistry (IOC), Karlsruhe Institute of Technology (KIT) Kaiserstraße 12 76131 Karlsruhe Germany; e Institute of Biological and Chemical Systems (IBCS-FMS), Karlsruhe Institute of Technology (KIT) Kaiserstraße 12 76131 Karlsruhe Germany

## Abstract

Multi-resonant thermally activated delayed fluorescence (MR-TADF) emitters offer an attractive solution for the fabrication of colour-saturated organic light-emitting diodes (OLEDs). Still, their rigid frameworks often lead to slow reverse intersystem crossing and aggregation-caused quenching. To address these challenges and simultaneously introduce chiroptical properties in solution-processable materials, here we present the first examples of chiral intramolecular Förster resonance energy transfer (FRET) MR-TADF dendrimers, CzPBN-CzBN and CzPBA-CzBN. These emitters combine an MR-TADF core with chiral paracyclophane (CzP)-based donor dendrons linked to different acceptor units, benzonitrile for CzPBN-CzBN and benzoic acid for CzPBA-CzBN. While both dendrimers show narrowband TADF emission, the nature of the acceptor impacts their chiroptical properties. Specifically, CzPBA-CzBN exhibits circularly polarised luminescence (CPL), whereas CzPBN-CzBN does not due to a weaker circular dichroism signal. Conversely, in solution-processed hyperfluorescent (HF) OLEDs, the device with CzPBN-CzBN achieved a superior maximum external quantum efficiency (EQE_max_) of 19.7% and lower efficiency roll-off (EQE of 18.9% at 1000 cd m^−2^) compared to the device with CzPBA-CzBN (EQE_max/1000_ = 17.2/16.5%). These findings reveal an intrinsic trade-off between CPL response and HF device performance, demonstrating how precise modulation of the excited-state manifold *via* acceptor engineering can be used to tailor the properties of intramolecular sensitised TADF emitters.

## Abstract 2

We report the first examples of chiral MR-TADF dendrimers using an intramolecular FRET design. Tuning the acceptor reveals a trade-off between CPL and device efficiency. This design allows for selecting either CPL activity or high device efficiency.

## Graphical abstract

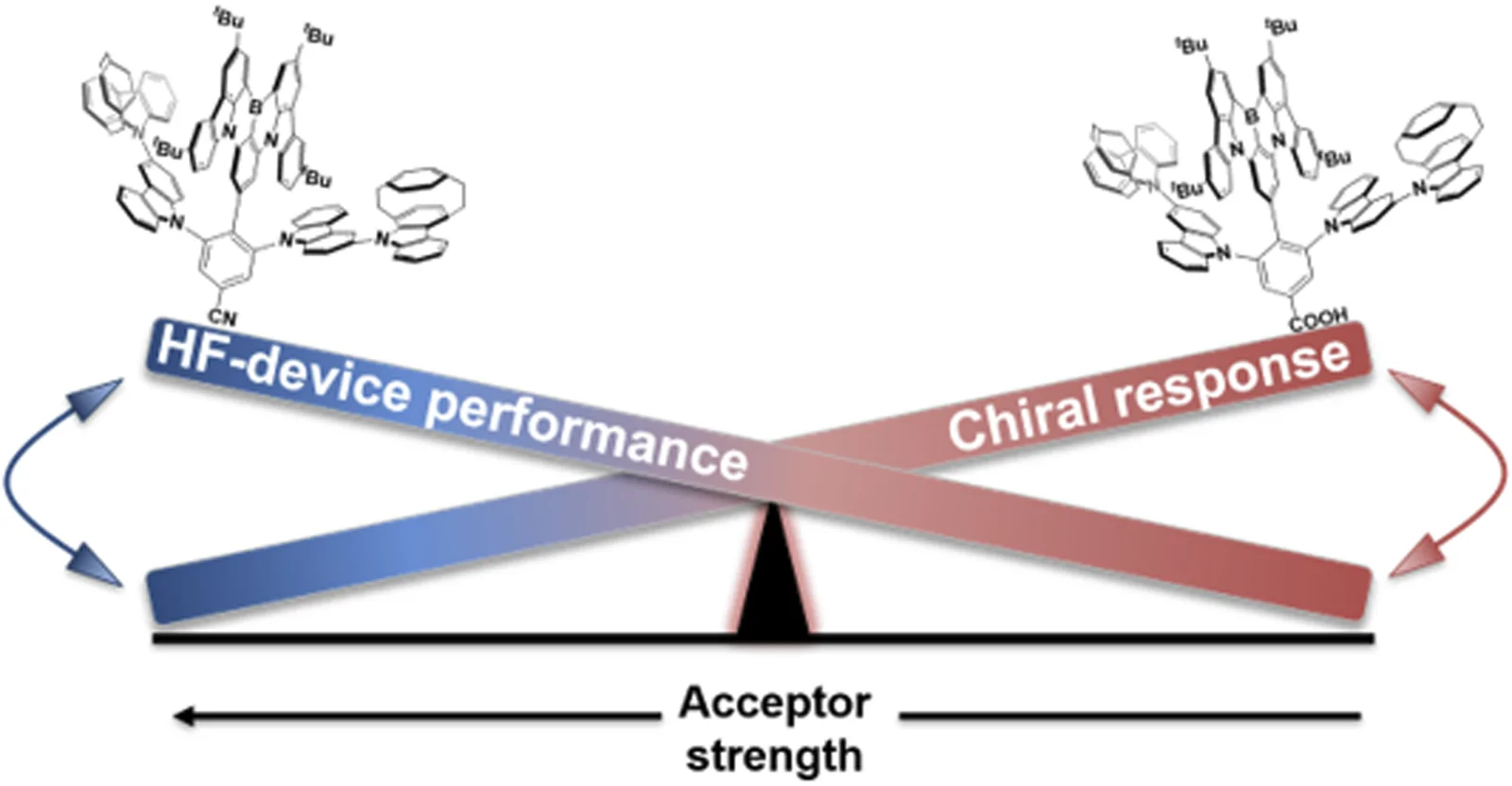

## Introduction

Thermally activated delayed fluorescence (TADF) emitters enable the harvesting of electrically generated singlet and triplet excitons, the latter *via* reverse intersystem crossing (RISC), allowing organic light-emitting diodes (OLEDs) to reach up to 100% internal quantum efficiency.^1^ Among TADF emitters, multiresonant TADF (MR-TADF) materials have garnered particular attention owing to their bright, narrowband emission arising from their rigid π-conjugated frameworks and short-range charge transfer (SRCT) excited states.^2–5^ However, the intrinsically slow RISC kinetics of many MR-TADF emitters (*k*_RISC_ often < 10^4^ s^−1^), combined with their planar and rigid structures, promote triplet accumulation and aggregation-caused quenching (ACQ), leading to pronounced efficiency roll-off, particularly in solution-processed (SP) devices.^1,5,6^ To overcome these intrinsic limitations, several complementary molecular design strategies have been developed to modulate the excited-state manifolds without sacrificing spectral purity. One effective approach is to decorate auxiliary donor–acceptor (D–A) TADF motifs onto MR-TADF moieties.^2,7,8^ By covalently linking a higher-energy D–A TADF unit to an MR-TADF core, excitons can be more effectively harvested using the former before energy transfer to the MR-TADF core, conserving the narrowband SRCT emission. For instance, the *k*_RISC_ of DtCzBN-CNBT2 ^2^ (Fig. 1) is 1.1 × 10^5^ s^−1^ and the *Φ*_PL_ is 97% (15 wt% in PhCzBCz), translating into SP-OLEDs with EQE_max_ values of 24.4% but showing strong efficiency roll-off (EQE_1000_ = 5.6%). This example demonstrates that intramolecular exciton harvesting cannot fully emulate so-called hyperfluorescent architectures without requiring ternary blends in SP OLEDs. A related concept has been implemented in dendrimeric MR-TADF emitters that are tailored for SP-OLEDs.^7^ Embedding a D–A TADF sensitising antenna and an MR-TADF emitting core within a single dendrimer decorated molecule enables efficient intramolecular Förster resonance energy transfer (FRET), while steric encapsulation suppresses ACQ. The second-generation dendrimer 2GtBuCzCO_2_HDCzB^7^ (Fig. 1) has a *Φ*_PL_ of 98% in 30 wt% doped films in mCP and a *k*_RISC_ on the order of 10^4^ s^−1^, alongside strong resistance to concentration-induced quenching. SP OLEDs achieved an EQE_max_ of 27.9% and low-efficiency roll-off out to >1000 cd m^−2^. These findings highlight the dual role of the donor dendrons in exciton management and ACQ control.

**Fig. 1:**
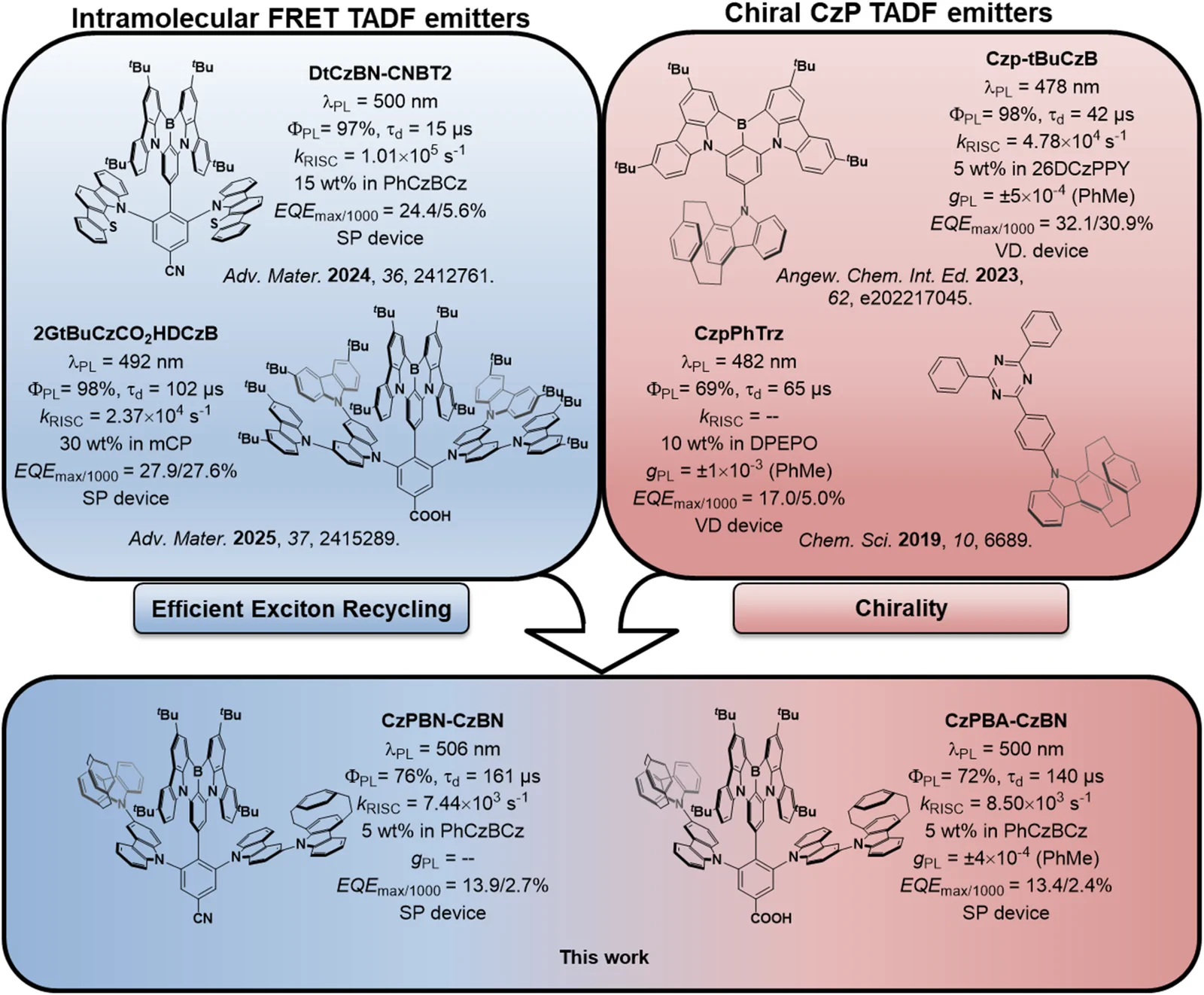
Chemical structures, key photophysical and device data of chiral CzP-based TADF emitters, intramolecular FRET TADF emitters and novel emitters presented in this work, CzPBN-CzBN and CzPBA-CzBN.

Beyond excited-state engineering, chemical modification of the donor itself to render it chiral offers a direct route for tuning the photophysical and chiroptical properties. In 2019, we introduced the carbazolophane (Czp) donor, an indole-annulated [2.2]paracyclophane, as a bulky, through-space-interacting donor motif for TADF emitter design. Conjugation of Czp with a triazine acceptor produced CzpPhTrz (Fig. 1).^9^ The use of Czp increased the donor-bridge torsion and delocalized the HOMO across the stacked cyclophane framework, decreasing Δ*E*_ST_ to 0.16 eV and activating efficient TADF at 10 wt% doping in DPEPO films, while the carbazole analogue (CzPhTRZ) has a significantly larger Δ*E*_ST_ (0.32 eV) and much weaker TADF character. Sky-blue OLEDs with CzpPhTrz showed an EQE_max_ of 17% and strong efficiency roll-off (EQE_1000_ = 5%). Enantiomerically pure samples exhibited mirror-image circular dichroism (CD) and circularly polarized luminescence (CPL) spectra, with *g*_PL_ of ±1 × 10^−3^ in toluene. More recently, planar chiral cyclophane motifs have been integrated directly with MR-TADF frameworks. For example, Czp-tBuCzB^10^ (Fig. 1) emits at *λ*_PL_ of 478 nm and has a *Φ*_PL_ of 98% (5 wt% in 26DCzPPY), where the rigid cyclophane scaffold suppressed aggregation and reduced efficiency roll-off in vacuum-deposited OLEDs (EQE_max_ = 32.1%; EQE_1000_ = 30.9%); the *g*_PL_ is on the order of 5 × 10^−4^ (in toluene).

Chiroptical information can be transmitted *via* FRET, for instance, from a chiral FRET donor to an achiral FRET acceptor, allowing the emitting species to exhibit CPL despite lacking intrinsic chirality. Such chiral FRET concepts provide a powerful strategy to decouple photophysical and chiroptical functionality by spatially segregating these moieties within a single molecular or supramolecular architecture.^11–13^

Collectively, these advances suggest that combining intramolecular exciton harvesting with chiral cyclophane-based donor engineering offers a promising route to simultaneously enhance RISC kinetics, suppress aggregation, and introduce chiroptical control in MR-TADF emitters. Guided by this rationale, here we report the design of an intramolecular FRET TADF dendrimer in which chiral donor dendron moieties are incorporated into the D–A unit of the intramolecular FRET dyad, enabling efficient exciton management alongside CPL. To the best of our knowledge, CzPBN-CzBN and CzPBA-CzBN represent the first examples of chiral TADF dendrimers and were designed by combining the MR-TADF core DtBuCzB and a D–A type emitter based either on a donor dendron decorated benzonitrile (CzPBN-CzBN) or benzoic acid (CzPBA-CzBN). In dilute toluene, CzPBN-CzBN and CzPBA-CzBN emit at *λ*_PL_ of 499 and 494 nm with a narrow envelope of 33 and 27 nm, respectively. Chiroptical measurements revealed distinct differences between CzPBN-CzBN and CzPBA-CzBN, where the latter shows a CPL response while the former does not due to weak circular dichroism (CD). When doped at 5 wt% into PhCzBCz both emitters maintain the narrowband emission (FWHM = 33 and 28 nm), and there is a clear temperature dependence of the delayed lifetime, confirming their TADF character. SP-OLEDs were fabricated, where binary devices exhibited moderate EQE_max_ of 13.9% (CzPBN-CzBN) and 13.4% (CzPBA-CzBN). To overcome the severe efficiency roll-off and moderate EQE_max_, hyperfluorescent devices were fabricated using 5*t*BuCzTRZ as the sensitiser. The EQE_max_ increased to 19.7% (CzPBN-CzBN) and 17.5% (CzPBA-CzBN), and more importantly, the efficiency roll-off was greatly reduced, with EQE_1000_ of 18.9% (CzPBN-CzBN) and 16.2% (CzPBA-CzBN) at Commission Internationale de l’Éclairage (CIE) coordinates of (0.15, 0.50) and (0.15, 0.44), respectively.

## Results and discussion

### Synthesis and characterisation

The synthesis was carried out in two steps, as shown in Scheme 1. Firstly, the donor dendron (CzP-Cz) was synthesised starting from 3-bromo-9-(*tert*-butyldimethylsilyl)-9*H*-carbazole^14^ to which 1^9^*H*-1(1,4)-carbazola-4(1,4)-benzenacyclohexaphane (CzP)^9^ was coupled under Buchwald–Hartwig type conditions. This was followed by silyl ether deprotection with TBAF, affording CzP-Cz in an overall yield of 69%. Two equivalents of CzP-Cz were then coupled onto DCzBDFCN^7^ in an S_N_Ar-type reaction.

**Scheme 1:**
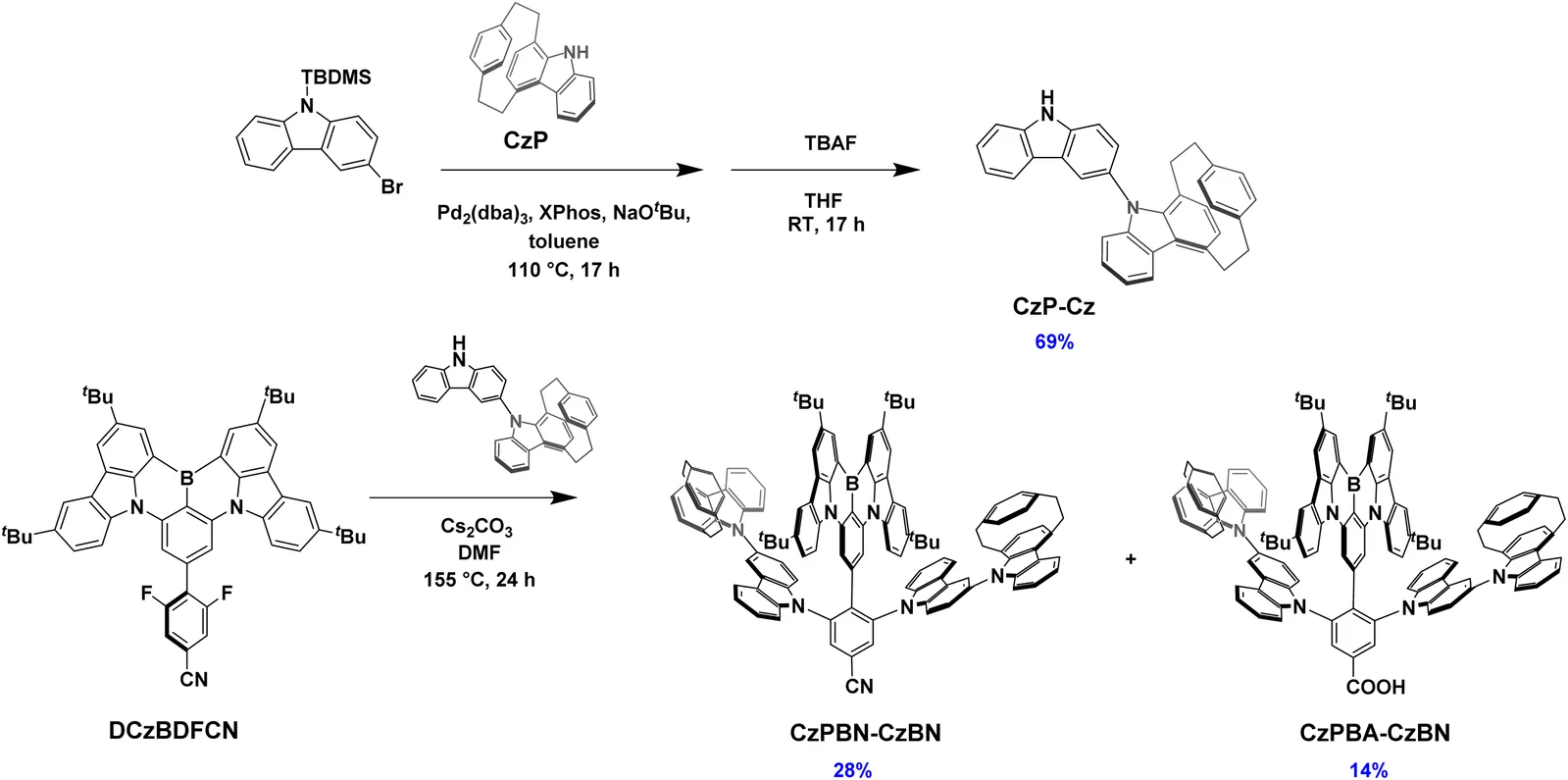
Synthetic route for CzPBN-CzBN and CzPBA-CzBN.

Despite the use of anhydrous dimethylformamide, a portion of the benzonitrile was hydrolysed to the corresponding benzoic acid. This hydrolysis was previously reported by our group;^7^ in that case the DMSO was sufficiently wet to promote the full conversion, whereas here in DMF only a partial conversion to the benzoic acid derivative was observed. CzPBN-CzBN and CzPBA-CzBN were isolated in low yields of 28 and 14%, respectively. The enantiopure derivatives (*R*_p_)-CzPBN-CzBN, (*R*_p_)-CzPBA-CzBN, (*S*_p_)-CzPBA-CzBN, and (*S*_p_)-CzPBN-CzBN were synthesised following the same route, starting from enantiopure CzP, the absolute configurations of the enantiomers were determined by comparison of the (*S*_p_)-CzP and (*R*_p_)-CzP with (*S*_p_)-4a and (*R*_p_)-4a, previously reported by our group (Fig. S15–S17).^15^ The structure and purity of CzPBN-CzBN and CzPBA-CzBN were confirmed by a combination of ^1^H, and 2D NMR spectroscopy (COSY), high-resolution mass spectrometry (HRMS), high-performance liquid chromatography (HPLC), infrared spectroscopy (IR) and melting point determination (Mp) (Fig. S18–S50). The observation of a single set of cross-peaks along the diagonal in the ^1^H–^1^H COSY NMR spectra, together with a single peak in the HPLC chromatograms, confirms the presence of a single molecular species. When combined with HRMS data, this provides conclusive evidence for the proposed structure.

### Theoretical calculations

To understand the impact of the use of CzP-based donor dendrons on the optoelectronic properties of CzPBN-CzBN and CzPBA-CzBN, we began with a computational study at the PBE0/6-31G(d,p) level of theory in the gas phase. This functional was chosen based on a benchmarking study by Moral *et al.*, which demonstrated the high accuracy of PBE0 functional for the predictions of the electronic structure of TADF emitters.^16^ To reduce computational costs, we replaced the *tert*-butyl groups of the central DtBuCzB unit with methyl groups (MeCzBN, Fig. 2), this modification has negligible impact on the electronic structure of the central DtBuCzB unit itself and therefore serves as a suitable model system (Fig. S3). The ground-state-optimised structures of the model compounds CzPBN-MeCzBN and CzPBA-MeCzBN possess similar geometries, with similar torsion angles and bond length between the benzonitrile/benzoic acid acceptor and the chiral CzP-based donor dendrons, and bond length between the benzonitrile/benzoic acid moiety and the MeCzBN. Only the torsion angle between the latter two differs slightly between CzPBN-MeCzBN (57.32°) and CzPBA-MeCzBN (51.10°), this difference potentially leads to minor differences in orbital overlap (Fig. S1, S2 and Table S2). Both CzPBN-MeCzBN and CzPBA-MeCzBN show similar HOMO and LUMO energies of −5.26/−2.18 and −5.18/−2.18 eV, respectively. To understand the interplay between the central MR-TADF moiety and the attached D–A TADF moiety, we also modelled the building blocks MeCzBN, CzPBN and CzPBA (Fig. 2). For both CzPBN-MeCzBN and CzPBA-MeCzBN, the HOMO is distributed across the CzP-Cz moiety, whereas the LUMO is localised on the MeCzBN core and extending to the benzonitrile or benzoic acid moiety. This is a similar orbital distribution to those present in CzPBN and CzPBA, suggesting a through-space charge transfer (TSCT) between the CzP-Cz donor dendrons and the central MeCzBN core.

**Fig. 2:**
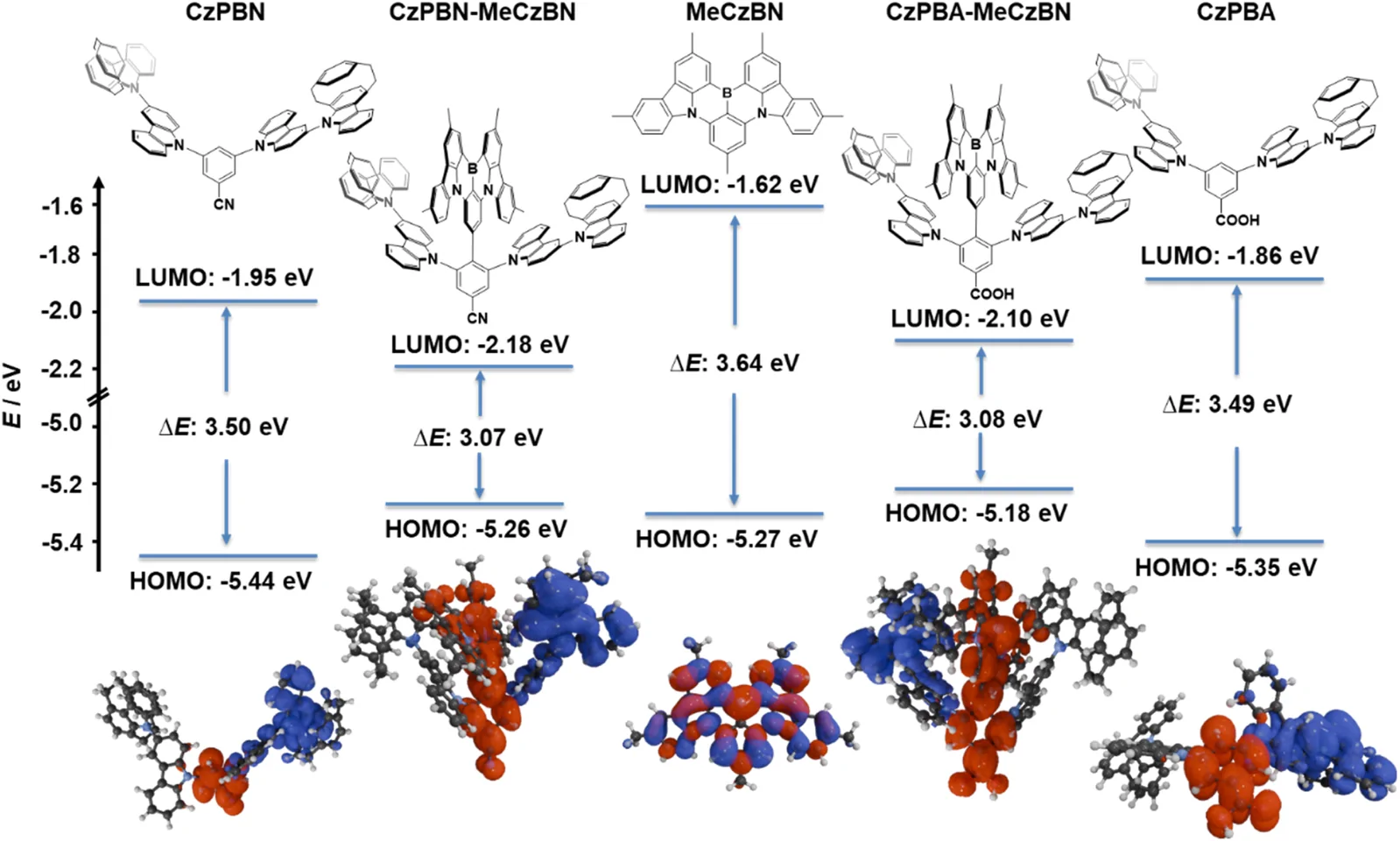
HOMO and LUMO energy levels and density plots (red = LUMO; blue = HOMO; isovalue = 0.02) for CzPBN-MeCzBN and CzPBA-MeCzBN, as well as the respective building blocks CzPBN, CzPBA and MeCzBN, calculated at PBE0/6-31G(d,p) level of theory in the gas phase.

The singlet and triplet excited-state energies of the model systems were calculated at the TDA-DFT-PBE0/6-31G(d,p) level of theory. The computed S_1_/T_1_ levels of CzPBN-MeCzBN and CzPBA-MeCzBN are 2.57/2.33 eV and 2.60/2.34 eV, respectively, and the corresponding Δ*E*_ST_ values are 0.25 and 0.26 eV. Density plots of the natural transition orbitals (NTOs) for the S_1_ state reveal a similar picture for both CzPBN-MeCzBN and CzPBA-MeCzBN, where the electron is distributed on the central MR-TADF unit and the benzonitrile or benzoic acid, while the hole is distributed across the CzP dendrons, consistent with a TSCT state. The T_1_ state is primarily localised on the MR-TADF MeCzBN core and exhibits SRCT character (Fig. 3). The fragments CzPBN and CzPBA are predicted to have higher energy S_1_/T_1_ states of 3.05/2.97 eV (CzPBN); 3.03/2.94 eV (CzPBA) of LRCT character (Fig. S4). Despite having similar excited-state energies (Fig. 3), CzPBN-MeCzBN and CzPBA-MeCzBN have different spin–orbit coupling matrix elements (SOCME) between these excited states. For CzPBN-MeCzBN, the SOCME is larger between the lower lying excited states (S_1_/T_3_ and S_2_/T_2_; Table S1) than for CzPBA-MeCzBN. However, for CzPBA-MeCzBN, a larger SOCME than for CzPBN-MeCzBN is predicted for the higher-lying excited states (S_3_/T_4_ and S_3_/T_5_; Table S1). These differences indicate the two emitters might utilize different RISC channels for the upconversion of triplet excitons to singlets. A smaller angle *θ*_*µ*,*m*_ of 45.09° compared to 59.68° was found for CzPBA-MeCzBN and CzPBN-MeCzBN upon comparison of the electric (*µ*) and magnetic (*m*) transition dipole moments, respectively, indicating a lower dissymmetry factor for the latter (more detailed discussion in the SI).

**Fig. 3:**
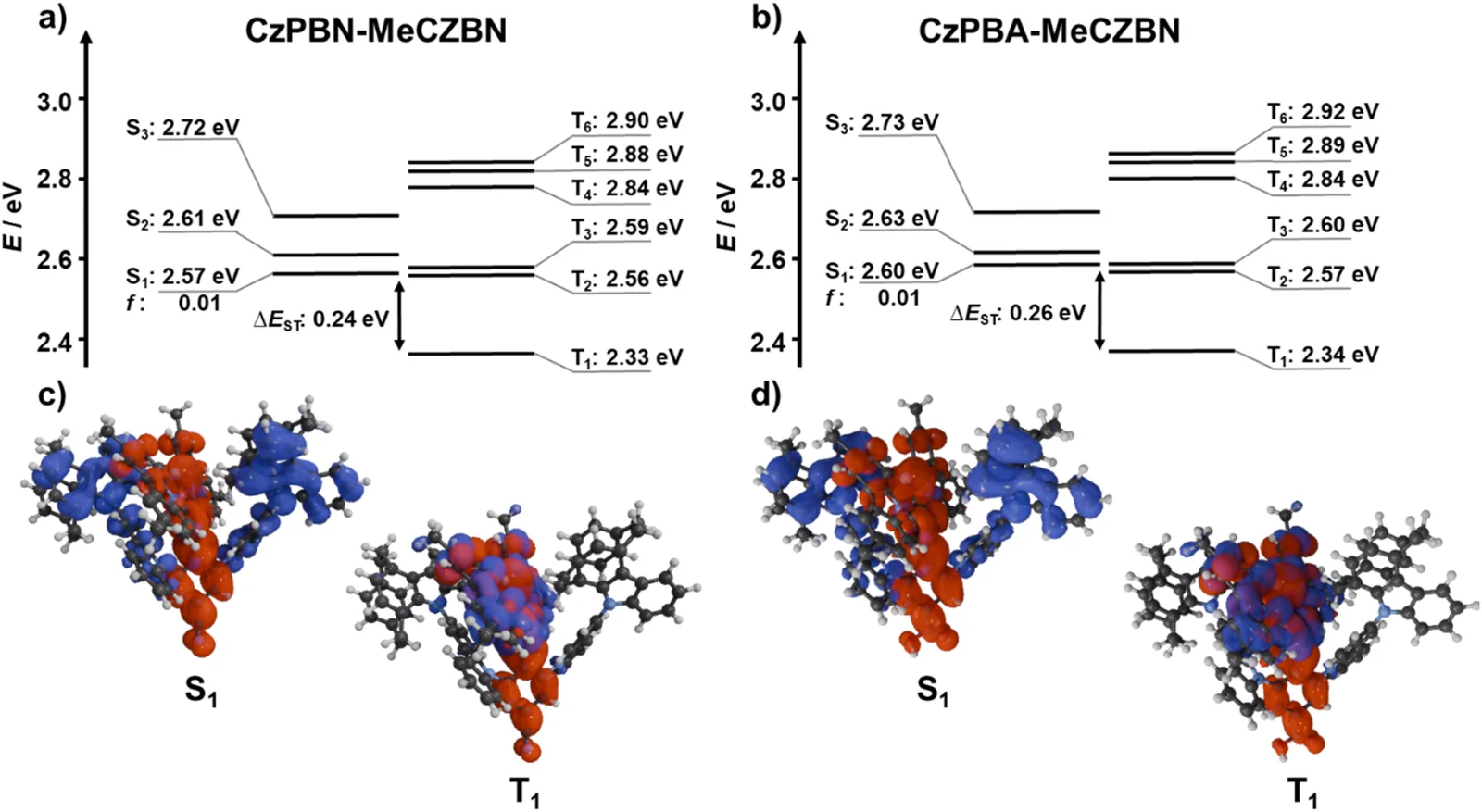
Excited-state manifolds for (a) CzPBN-MeCzBN and (b) CzPBA-MeCzBN. NTO density plots for the S_1_ and T_1_ states of (c) CzPBN-MeCzBN and (d) CzPBA-MeCzBN (red = electron; blue = hole; isovalue = 0.02). Calculated at PBE0/6-31G(d,p) level of theory in the gas phase.

### Photophysical properties

The absorption spectra of CzPBN-CzBN and CzPBA-CzBN in toluene are similar, with a low-energy band at 477 nm, assigned to the SRCT transition of the central MR-TADF core, which is red-shifted compared to that of DtBuCzB (*λ*_abs_ = 468 nm, *ε* = 82× 10^3^ M^−1^ cm^−1^).^17^ The intensity of the SRCT band is diminished, with molar absorptivity coefficients, *ε*, of 35 × 10^3^ and 51 × 10^3^ M^−1^ cm^−1^ for CzPBN-CzBN and CzPBA-CzBN, respectively. These absorption bands are in good agreement with the theoretically predicted vertically excited transitions to the S_1_ state (CzPBN-CzBN = 2.57 eV = 482 nm; CzPBA-CzBN = 2.60 eV = 477 nm). The absorption spectra of both compounds show a band around 390 nm, which is noticeably more intense in CzPBN-CzBN than in CzPBA-CzBN. Comparison with the theoretically calculated absorption spectra and excited-state energy levels of the corresponding fragments, CzPBN (S_1_ = 3.05 eV) and CzPBA (S_1_ = 3.03 eV) (Fig. S4 and S5), indicates that this transition corresponds to a long-range charge transfer (LRCT) localised on the donor-substituted benzonitrile or benzoic acid unit, respectively. The higher intensity of this band in CzPBN-CzBN is consistent with the computational results, which predict a stronger transition for the nitrile-containing derivative.

CzPBN-CzBN and CzPBA-CzBN show narrowband blue-green emission in toluene, with photoluminescence (PL) maxima *λ*_PL_, peaking at 499 and 494 nm and full width at half maxima, FWHM, of 33 and 27 nm, respectively. Their PL quantum yields, *Φ*_PL_, in degassed toluene are similar at 71 and 70%, respectively, for CzPBN-CzBN and CzPBA-CzBN. These decrease to 68 and 61% upon exposure to air. Time-resolved PL (TRPL) measurements of the toluene solutions revealed prompt lifetimes, *τ*_p_, of 6.9 and 6.4 ns for CzPBN-CzBN and CzPBA-CzBN, respectively, and a weak delayed emission (Fig. 4c) that disappears upon exposure to air (Fig. S6); however, this component was too weak for a reliable fit to be derived from the data, and therefore we do not report a delayed lifetime, *τ*_d_.

**Fig. 4:**
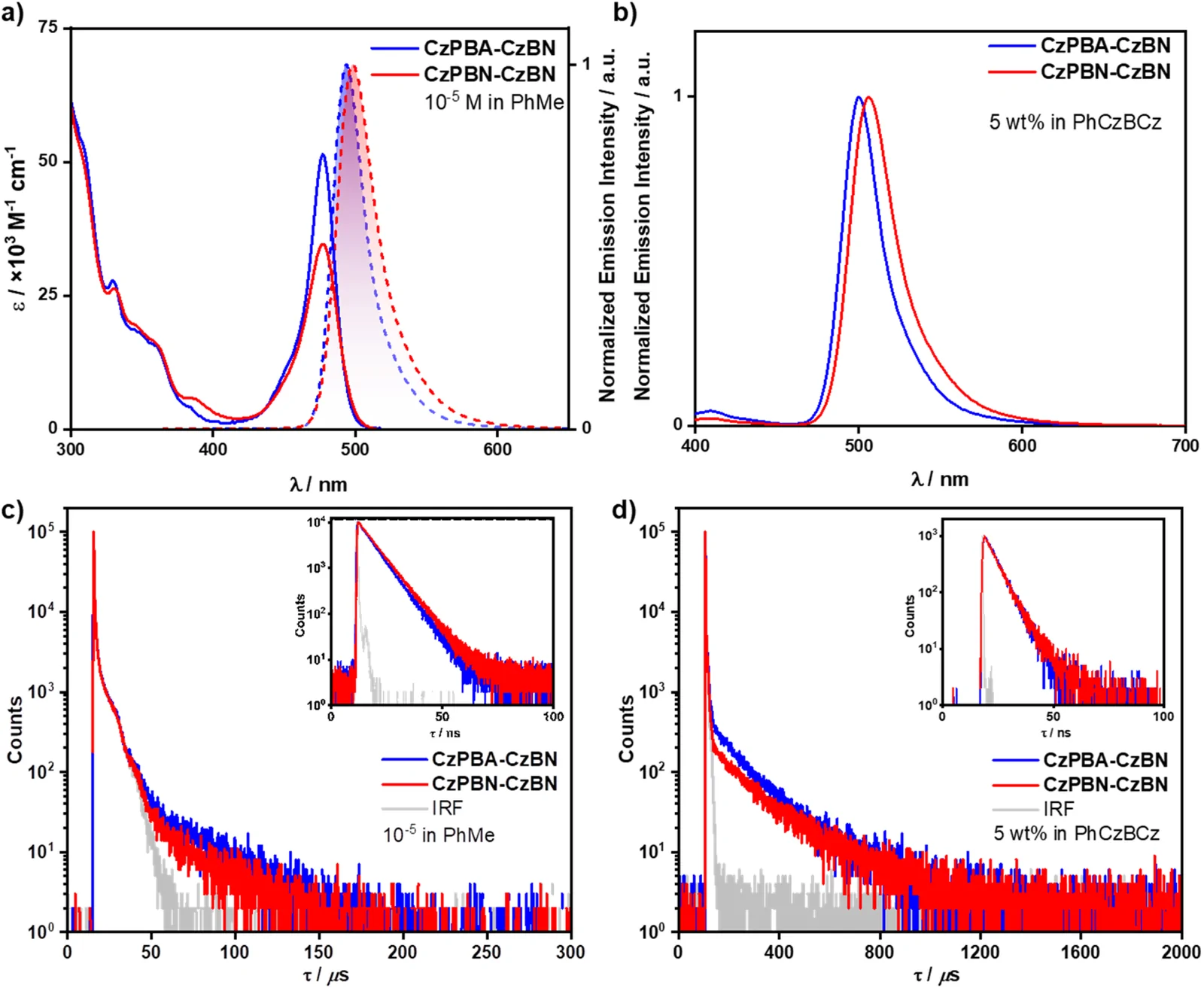
(a) Absorption and normalized PL spectra in dilute toluene solution (10^−5^ M; *λ*_exc_ = 350 nm); (b) normalized PL spectra of 5 wt% doped films in PhCzBCz (*λ*_exc_ = 340 nm); (c) TRPL decays (*λ*_exc_ = 350 nm); Inset: TRPL decay of the prompt fluorescence (*λ*_exc_ = 375 nm) in dilute toluene solution (10^−5^ M) (d) TRPL decays (*λ*_exc_ = 340 nm); Inset: TRPL decay of the prompt fluorescence (*λ*_exc_ = 375 nm) of 5 wt% doped films of CzPBN-CzBN (red) and CzPBA-CzBN (blue) in PhCzBCz.

The S_1_ and T_1_ energies of both emitters were determined from PL measurements in 2-methyltetrahydrofuran (2-MeTHF) glass at 77 K. The maxima of the steady-state PL (SSPL) spectra were used to infer the S_1_ energies. In contrast, the maxima of the time-gated PL spectra (10–85 ms acquisition after excitation) were used to estimate the T_1_ energies. CzPBN-CzBN has comparable S_1_ and T_1_ levels of 2.56 and 2.40 eV to those of CzPBA-CzBN (2.58 and 2.42 eV). The corresponding Δ*E*_ST_ value of 0.16 eV is the same for CzPBN-CzBN and CzPBA-CzBN. There is a small positive solvatochromism shift in the SSPL spectra of both CzPBN-CzBN and CzPBA-CzBN as a function of solvent polarity (Fig. S7 and Table S3), confirming the SRCT character of the emissive singlet state.

Given the attractive set of photophysical properties in toluene, we next conducted thin film measurements to evaluate the photophysical properties of CzPBN-CzBN and CzPBA-CzBN in the solid state. We chose PhCzBCz as a host matrix due to its sufficiently high triplet energy of 2.96 eV ^18^ and prior reports demonstrating high-efficiency OLEDs with this host material.^2,19–23^ A doping ratio of 5 wt% was found to be the best compromise between ensuring an efficient energy transfer from the host to the emitter, as well as maintaining a high *Φ*_PL_ (Fig. S9 and Table S4). Both CzPBN-CzBN and CzPBA-CzBN show narrowband green emission at *λ*_PL_ of 506 and 500 nm and FWHM values of 33 and 28 nm, respectively. These values are modestly red-shifted compared to those in toluene, and there is no broadening of the emission band. As such, the red-shift is ascribed due to changes in the polarity of the medium rather than any measurable aggregate formation. The *Φ*_PL_ values of 76 and 72% for CzPBN-CzBN and CzPBA-CzBN, respectively, under N_2_ are slightly higher than those in solution, while there is a more pronounced drop to 60 and 55% under air. TRPL measurements show similar decay kinetics, with *τ*_p_ of 5.6 and 5.4 ns and *τ*_d_ of 161 and 140 µs. Both emitters show the same delayed fluorescence efficiency, *Φ*_d_, of 12%, while the prompt fluorescence efficiency, *Φ*_p_, of CzPBN-CzBN is slightly higher at 64% compared to 60% for CzPBA-CzBN. Temperature-dependent TRPL measurements reveal the expected temperature dependence of the delayed emission, confirming TADF behaviour for both compounds (Fig. S10). The singlet and triplet energies were determined from the maxima of the steady-state PL and time-gated emission spectra measured at 77 K. S_1_/T_1_ levels of 2.45/2.29 and 2.48/2.32 eV were found for CzPBN-CzBN and CzPBA-CzBN, respectively, resulting in the same Δ*E*_ST_ of 0.16 eV. The excitonic rate constants are compiled in Table 1. CzPBN-CzBN and CzPBA-CzBN have similar *k*_r_ and *k*_ISC_ values of approximately 1.1 × 10^8^ and 3 × 10^7^ s^−1^. In contrast, both *k*_nr_ (4.36 × 10^7^ s^−1^) and *k*_RISC_ (8.50 × 10^3^ s^−1^) are slightly accelerated for the films of CzPBA-CzBN compared to its nitrile counterpart CzPBN-CzBN (*k*_nr_ = 3.60 × 10^7^ s^−1^; *k*_RISC_ = 7.44 × 10^3^ s^−1^). Given the experimentally same Δ*E*_ST_ of 0.16 eV, the relative faster *k*_RISC_ (∼14%) of CzPBA-CzBN compared to CzPBN-CzBN is not attributable to the Δ*E*_ST_, but instead is to the larger spin–orbit coupling. The largest computed asymmetry of SOCME values is at S_2_/T_2_, favouring CzPBN-MeCzBN (0.2648 *vs.* 0.0227 cm^−1^), while S_3_/T_4_ favours CzPBA-MeCzBN (0.1765 *vs.* 0.1193 cm^−1^). As *k*_RISC_ is dependent on the square of the SOCME, these channel-level differences are far larger than the difference in experimental *k*_RISC_, suggesting RISC proceeds through a combination of these channels rather than one dominant pathway.

**Table 1:** Photophysical data of CzPBN-CzBN and CzPBA-CzBN in dilute toluene and 5 wt% doped films in PhCzBCz

Emitter	Medium	*λ*_Abs_ (*ε*)^c^/nm (×10^3^ M^−1^ cm^−1^)	*λ*_PL_ (FHWM)^d^/nm	*E*_S_1_/T_1__ e/eV	Δ*E*_ST_^f^/eV	*Φ*_PL_ g/%	*Φ*_p_/*Φ*_d_^h^/%	*τ*_p_ i/ns	*τ*_d_ i/µs	*k*_ISC_ j/×10^7^ s^−1^	*k*_RISC_ j/×10^3^ s^−1^	*k*_s_r_ j/×10^8^ s^−1^	*k*_s_nr_ j/×10^7^ s^−1^
CzPBN-CzBN	Sol.^a^	477 (35)	499 (33)	2.56/2.40	0.16	71/68	—	6.9	—	—	—	—	—
	film^b^	—	506 (33)	2.45/2.29	0.16	76/60	12/64	5.6	161	2.96	7.44	1.14	3.60
CzPBA-CzBN	Sol.^a^	477 (51)	494 (27)	2.58/2.42	0.16	70/61	—	6.4	—	—	—	—	—
	film^b^	—	500 (28)	2.48/2.38	0.16	72/55	12/60	5.4	140	2.86	8.50	1.12	4.36

a In toluene solutions (10^−5^ M).

b in spin-coated 5 wt% doped films of emitters in PhCzBCz.

c Lowest energy absorbance peak, absorptivity (*ε*) in parentheses (/10^3^ M^−1^ cm^−1^).

d Steady-state emission maximum and full width at half maximum at 300 K (*λ*_exc_ = 350 nm (sol.); *λ*_exc_ = 340 nm (film)).

e S_1_ and T_1_ energies obtained from the maxima of the respective steady-state PL and phosphorescence spectra (delay: 10 ms; gate time: 85 ms) at 77 K. Solution samples were prepared in 2-MeTHF (10^−5^ M) (*λ*_exc_ = 350 nm (sol.); *λ*_exc_ = 340 nm (film)).

f Δ*E*_ST_ = *E*(S_1_) − *E*(T_1_).

g Relative *Φ*_PL_ in solutions were measured by a comparative method using quinine sulfate as a standard (*Φ*_r_ = 54.6% in 1 N H_2_SO_4_),.^24^ In contrast, absolute *Φ*_PL_ of the thin films were measured using an integrating sphere. Solutions were measure in degassed/aerated solvent, while films were measured under N_2_/air atmosphere.

h Prompt, *Φ*_p_ and the delayed *Φ*_d_ portion of emission.

i Prompt and delayed lifetimes were obtained by TCSPC (*λ*_exc_ = 375 nm sol and film) and MCS (*λ*_exc_ = 350 nm (sol.); *λ*_exc_ = 340 nm (film)), respectively.

j Intersystem and reverse intersystem crossing rates were calculated using the steady-state approximation method, as described in literature.^25^

### Chiroptical properties

We next explored the chiroptical properties of enantiomerically pure samples of CzPBN-CzBN and CzPBA-CzBN. Fig. 5a shows the mirror-image circular dichroism (CD) spectra of the enantiomers (*S*_p_)-CzPBA-CzBN and (*R*_p_)-CzPBA-CzBN in toluene. The CD spectrum of (*R*_p_)-CzPBA-CzBN displays three positive bands at 314, 365 and 475 nm of moderate intensities (Δ*ε* ∼ 20 M^−1^ cm^−1^) and a weak negative band at 331 nm (Δ*ε* ∼ −5 M^−1^ cm^−1^). In comparison, the CD spectrum of (*R*_p_)-CzPBN-CzBN displays four positive bands at 314, 343, 356, and 396 nm (Δ*ε* ∼ 16–20 M^−1^ cm^−1^), accompanied by a weaker positive band at 465 nm (Δ*ε* ∼ 5 M^−1^ cm^−1^, Fig. 5b). The different intensities of the lower energy bands of the enantiomers of CzPBA-CzBN (51 × 10^3^ M^−1^ cm^−1^) and CzPBN-CzBN (35 × 10^3^ M^−1^ cm^−1^), as well as the weaker intensity CD bands for CzPBN-CzBN compared to CzPBA-CzBN have a direct consequence on the circular polarised light (CPL) responses measured in toluene. Indeed, the enantiomers of CzPBA-CzBN display clear mirror-image CPL signals at 490 nm (*λ*_exc_ = 350 nm, Fig. 5c), with emission dissymmetry factors, *g*_PL_, of ±4 × 10^−4^ (Fig. 5d). These values are in the same range as those reported for other CzP-functionalized TADF emitters (*g*_PL_ = ±5 × 10^−4^ for Czp-tBuCzB; ±1 × 10^−3^ for CzpPhTrz),^9,10^ underlining the efficient chirality transfer *via* FRET in (*S*_p_)-CzPBA-CzBN and (*R*_p_)-CzPBA-CzBN. The CPL signals for (*R*_p_)-CzPBN-CzBN and (*S*_p_)-CzPBN-CzBN appear to be too weak to be observed, which is based on their weaker CD spectra (especially in the lower-energy region where CD and CPL are correlated). These results demonstrate that exchanging the acceptor unit in the D–A TADF moiety directly modulates the excited-state manifold. In CzPBA-CzBN, this modification produces an energetic alignment that enables efficient energy transfer from a CPL-emissive state (localised on CzPBA-Cz) to the SRCT emissive state (localised on DtBuCzB). By contrast, in CzPBN-CzBN the excited states are arranged such that this energy transfer pathway is not energetically favoured and therefore does not occur efficiently. Due to the low CD response of the CzPBA-CzBN enantiomers measurements of the *g*_PL_ in the solid state did not yield conclusive results and are therefore not presented within this work.

**Fig. 5:**
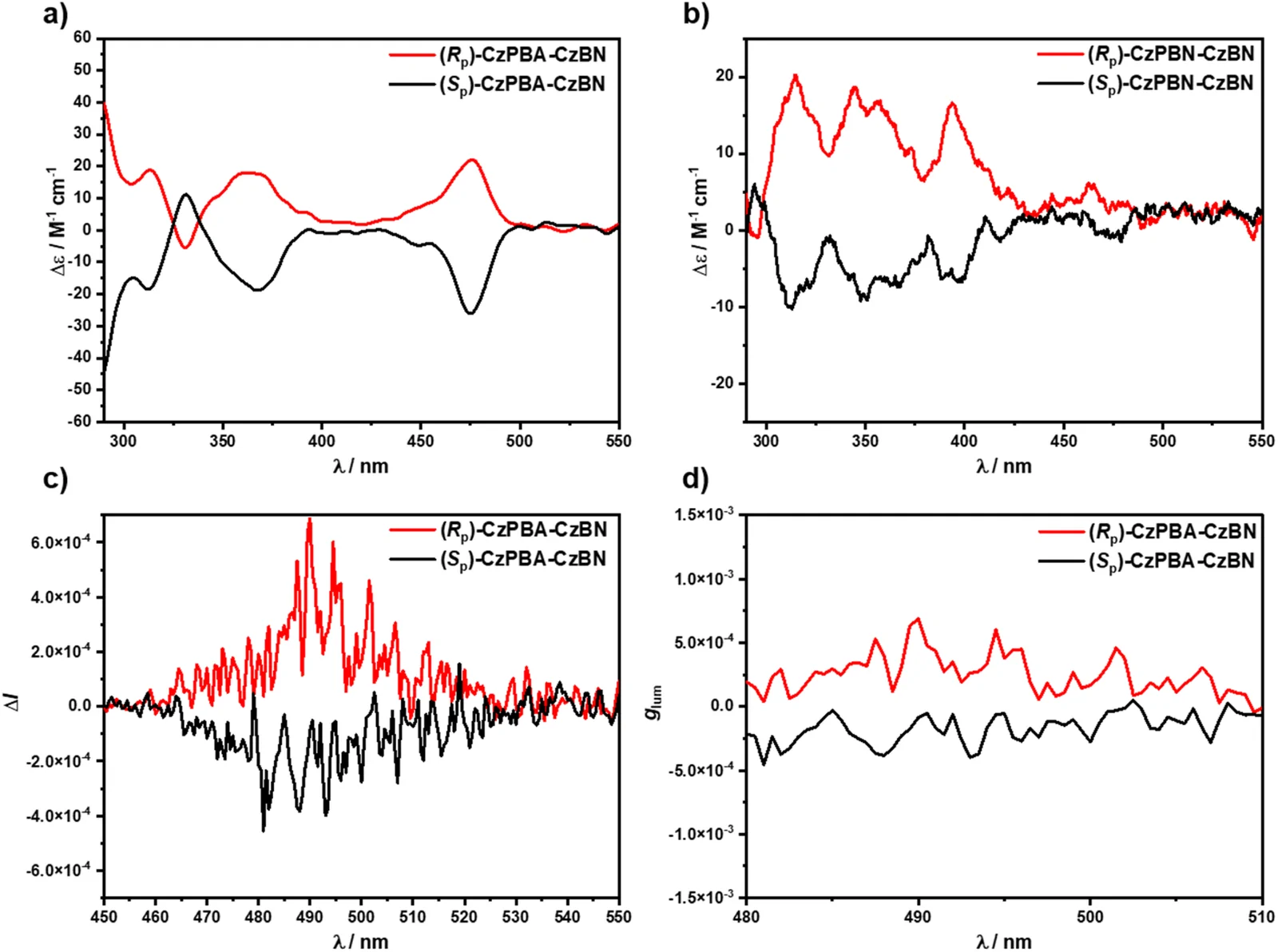
CD spectra of (a) (*R*_p_)-CzPBA-CzBN (red) and (*S*_p_)-CzPBA-CzBN (black) and (b) (*R*_p_)-CzPBN-CzBN (red) and (*S*_p_)-CzPBN-CzBN (black) in dilute toluene solution (10^−6^ M). (c) CPL and (d) *g*_lum_ spectra of (*R*_p_)-CzPBA-CzBN (red) and (*S*_p_)-CzPBA-CzBN (black) in dilute toluene solution (10^−6^ M; *λ*_exc_ = 350 nm).

### Electrochemistry

Given their potential as OLED emitters, we conducted cyclic voltammetry (CV) and different pulse voltammetry (DPV) experiments in dichloromethane (DCM) using [^*n*^Bu_4_N]PF_6_ (0.1 M) as the supporting electrolyte and ferrocene/ferrocenium (Fc/Fc^+^) as an internal standard to infer the HOMO and LUMO energies. The CV measurements showed irreversible reduction waves for CzPBN-CzBN and CzPBA-CzBN at reduction potentials (*E*_red_) of −1.71 and −1.81 V, respectively, *vs.* a standard calomel electrode (SCE). The oxidation waves are quasi-reversible at oxidation potentials, *E*_ox_, of 1.01 and 0.85 V for CzPBN-CzBN and CzPBA-CzBN, respectively (Fig. S14 and Table S6). These values are similar to the ones found for DtBuCzB (*E*_red_ = −1.73 V; *E*_ox_ = 1.09 V; Fig. S14), which resembles the MR-TADF moiety in CzPBN-CzBN and CzPBA-CzBN. For CzPBA-CzBN the oxidation potential is cathodically shifted, indicating oxidation occurs on the carboxylic acid moiety, rather than on the MR-TADF unit. The HOMO/LUMO energy levels, derived from these potentials are −5.35/−2.63 and −5.19/−2.53 eV. These values are in good agreement with the computed values (Fig. 2). They are similar to those of DtBuCzB (−5.39/−2.57 eV) and the structurally related compounds 2GtBuCzCO_2_HDCzB (Fig. 1), (HOMO/LUMO = −5.40/−2.63 eV)^7^ and DtCzBN-CNBT1 (Fig. 1, HOMO/LUMO = −5.45/−2.69 eV).^2^ The resulting HOMO/LUMO energy gaps are 2.72 eV for CzPBN-CzBN and 2.66 eV for CzPBA-CzBN.

### Electroluminescence properties

Given the good solubility and high molecular weight of CzPBN-CzBN and CzPBA-CzBN, we next proceeded to fabricate SP OLEDs using the following device stack: indium tin oxide (ITO)/poly(3,4-ethylenedioxythiophene):polystyrene sulfonate (PEDOT:PSS) (35 nm)/EML (25 nm)/TmPyPB (40 nm)/LiF(1 nm)/Al (100 nm). For guest/host systems, 5 wt% of racemic CzPBN-CzBN or CzPBA-CzBN were doped in PhCzBCz, while for sensitised (HF) devices, 2 wt% of racemic CzPBN-CzBN or CzPBA-CzBN and 10 wt% of the TADF sensitizer 5*t*BuCzTRZ were doped in PhCzBCz. In these devices, ITO and Al were used as the anode and cathode, respectively. PEDOT:PSS, TmPyPB and LiF were used as the hole injection and transporting, electron transporting and electron injection layers, respectively. The EL spectra, current density–voltage–luminance (*J*–*V*–*L*) curves, and EQE–*L* spectra are shown in Fig. 6, and the key device data are summarised in Table 3.

**Fig. 6:**
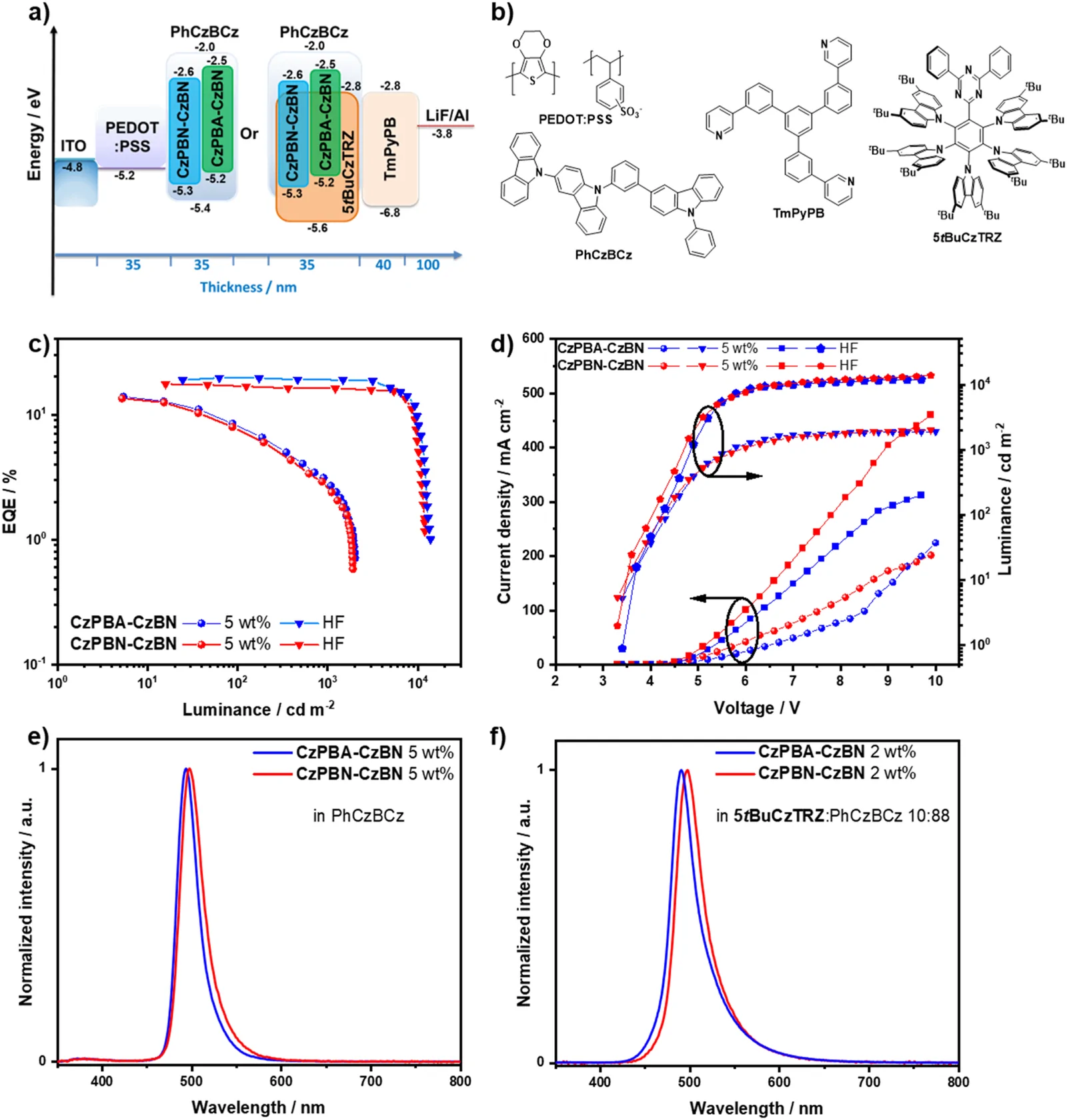
(a) Solution-processed OLED structure for binary and ternary devices. (b) Chemical structures of the materials used. (c) EQE–*L* curves, (d) *J*–*V*–*L* curves, (e) electroluminescence spectra of binary devices, and (f) electroluminescence spectra of ternary devices of CzPBN-CzBN (red) and CzPBA-CzBN (blue).

The EL spectra of CzPBN-CzBN and CzPBA-CzBN align with the corresponding narrowband PL emission spectra of the 5 wt% doped film in PhCzBCz (Fig. 6e and 4b). The devices emit in the green region, with *λ*_EL_ values of 499 and 493 nm and FWHM values of 30 and 27 nm, respectively. The corresponding Commission Internationale de l’éclairage (CIE) coordinates for the devices with CzPBN-CzBN and CzPBA-CzBN are (0.11, 0.51) and (0.10, 0.47), respectively. The devices with CzPBN-CzBN and CzPBA-CzBN exhibited turn-on voltages of 3.5 V and maximum EQE (EQE_max_) of 13.9 and 13.4%, respectively. However, in contrast to previous studies^2,7^ utilizing a similar design principle both devices exhibited a significant efficiency roll-off, as the EQEs at 1000 cd m^−2^ (EQE_1000_) plummeting to 2.7 and 2.4%, respectively. This was attributed to inefficient energy transfer from the host to the emitters at high luminance values (host emission is visible in the EL spectra, Fig. 6e) and the relatively slow *k*_RISC_ of CzPBN-CzBN and CzPBA-CzBN (Table 1). To address these issues and improve the device performance, a donor–acceptor type TADF emitter 5*t*BuCzTRZ was employed as a sensitiser in a ternary device (HF) stack to facilitate the exciton utilisation, in which a terminal intramolecular sensitised emitter still is beneficial^26^ The emission from 5*t*BuCzTRZ is broad, peaking at *λ*_PL_ of 497 nm (FWHM of 90 nm). There is a strong overlap between this emission and the intense SRCT absorption bands of CzPBN-CzBN and CzPBA-CzBN in toluene (Fig. S11), making FRET likely to be efficient. Moreover, 5*t*BuCzTRZ possesses a fast *k*_RISC_ of 5.52 × 10^6^ s^−1^ in 10 wt% doped films in PhCzBCz (Table S5). The ternary films consisting of CzPBN-CzBN or CzPBA-CzBN : 5*t*BuCzTRZ : PhCzBCz (2 : 10 : 88 wt%) have high *Φ*_PL_ values of ∼100% and 81% and very fast *τ*_d_ of 0.50 and 0.52 µs, respectively (Table 2 and Fig. S12).

**Table 2:** Photophysical properties of ternary films

Film^a^	*λ*_PL_ b/nm	FWHM^c^/nm	*Φ*_PL_ N_2_/air^d^/%	*τ*_p_ e/ns	*τ*_d_ e/µs
CzPBN-CzBN HF	503	33	∼100/86	14.7	0.50
CzPBA-CzBN HF	497	30	81/61	15.9	0.53

a Films consisting of emitter : 5*t*BuCzTRZ : PhCzBCz 2 : 10 : 88 wt%.

b Steady-state PL maximum.

c Full width at half maximum.

d Absolute *Φ*_PL_ of the thin films were measured using an integrating sphere under N_2_ and air atmosphere (*λ*_exc_ = 340 nm).

e Prompt and delayed lifetimes were obtained by TCSPC (*λ*_exc_ = 375 nm).

**Table 3:** Electroluminescence data of solution-processed OLEDs of CzPBN-CzBN and CzPBA-CzBN

Device	*V*_on_ a/V	*λ*_EL_ b/nm	FWHM^c^/nm	EQE_max/100/1000_^d^/%	*L*_max_ e/cd m^−2^	CIE^f^/(*x*, *y*)
CzPBN-CzBN 5 wt%	3.5	499	30	13.9/8.5/2.7	2010	(0.11, 0.51)
CzPBA-CzBn 5 wt%	3.5	493	27	13.4/8.0/2.4	1900	(0.10, 0.47)
CzPBN-CzBN HF	3.3	498	35	19.7/19.5/18.9	13 900	(0.15, 0.50)
CzPBA-CzBN HF	3.3	491	34	17.5/16.8/16.2	12 100	(0.15, 0.44)

a Turn-on voltage.

b Electroluminescence peak wavelength.

c Electroluminescence FWHM.

d External quantum efficiency at maximum, 100 cd m^−2^ and 1000 cd m^−2^.

e The maximum luminance.

f Commission Internationale de L’Éclairage coordinates.

Consequently, the HF devices incorporating 10 wt% 5*t*BuCzTRZ delivered higher EQE_max_ values of 19.7 and 17.5%, and significantly suppressed efficiency roll-off, with EQE_1000_ values of 18.9 and 16.2% and EQE_5000_ values of 16.3 and 15.4%, for the devices with CzPBN-CzBN and CzPBA-CzBN, respectively. The relatively higher EQE_max_ values for the HF devices with CzPBN-CzBN can be explained by the superior *Φ*_PL_ of the respective ternary films compared to the one with CzPBA-CzBN. This shows that the difference in HF device efficiency between the two emitters tracks with the differing *Φ*_PL_ of their ternary films, arising from differences in FRET efficiency and/or non-radiative decay pathways introduced by the acceptor modification rather than from differing chiroptical properties of the emitters. The host emission was effectively suppressed in the HF devices, and the turn-on voltage was slightly reduced to 3.3 V. The HF devices with CzPBN-CzBN and CzPBA-CzBN emitted at 498 and 491 nm, with FWHM values of 34 and 33 nm, respectively, yielding CIE coordinates of (0.15, 0.50) and (0.15, 0.50). These results underscore the value of using HF device structures to improve the performance of SP-OLEDs.

## Conclusions

In this work, we presented two novel chiral MR-TADF dendrimers, which, to the best of our knowledge, represent the first examples of this material class. CzPBN-CzBN and CzPBA-CzBN employ an intramolecular FRET design and exhibit similar yet slightly distinct photophysical properties, indicating that the functional groups (nitrile or carboxylic acid) on the pendant D–A TADF moiety indeed manipulate the excited-state manifold ever so slightly. Both emitters show narrowband TADF emission. Interestingly, the biggest differences between CzPBN-CzBN and CzPBA-CzBN are found within their chiroptical properties, where CzPBN-CzBN shows a much weaker CD signal compared to CzPBA-CzBN, resulting in no observable CPL for CzPBN-CzBN, while CzPBA-CzBN shows a CPL response. This difference indicates that the modulation of the acceptor on the D–A TADF emitter indeed has an impact on excited state manifolds and pathways. When comparing the performances of SP OLEDs using both emitters, which are comparable; however, in HF SP-OLEDs, the device with CzPBN-CzBN shows a higher EQE_max_ and lower efficiency roll-off compared to the device with CzPBA-CzBN. The reason for this divergence in performance correlates with the more efficient FRET between the 5*t*BuCzTRZ sensitiser and CzPBN-CzBN, reflected in the higher *Φ*_PL_ of its ternary films. Taken together, these results show that acceptor modification (benzonitrile *vs.* benzoic acid) on the pendant D–A TADF unit is an effective handle for tuning the chiroptical response, the photophysical properties, and the HF device performance of intramolecular sensitised TADF dendrimers. Within this emitter pair we observe an inverse relationship between CPL response and HF device performance; however, as only two emitters were compared, and as the difference in HF device efficiency can be substantially accounted for by the different *Φ*_PL_ of the ternary films, we cannot conclude at this stage that this relationship constitutes a general, intrinsic trade-off. Establishing whether CPL response and HF device efficiency are mechanistically coupled rather than coincidentally correlated in general will require a broader study of emitters and represents an interesting direction for future work.

## Author contributions

M. F.: conception, investigation, methodology, visualisation, writing – original draft, writing – review & editing. Y. X.: conception, investigation, writing – review & editing. D. C.: investigation, visualisation, writing – original draft. J. S. O. O.: investigation, visualisation. P. C.: investigation. J. S.: investigation. S. B.: project management, supervision, resources, writing – review & editing. J. C.: project management, supervision, resources, writing – original draft. X.-H. Z.: project management, supervision, resources, writing – review & editing. E. Z.-C.: project management, supervision, resources, writing – review & editing.

## Conflicts of interest

The authors declare no conflicts of interest.

## Supplementary Material

SC-017-D6SC04906K-s001

SC-017-D6SC04906K-s002

## Data Availability

The research data supporting this publication can be accessed at the https://doi.org/10.17630/d533bb08-fee2-4cda-b6c3-89ebaf52ea78. Data that refers to the chiral separation are submitted to the repository Chemotion (https://www.chemotion-repository.net/). The DOI minted for the data are linked in the supplementary information (SI) and can be gained with the collection DOI https://dx.doi.org/10.14272/collection/JTS_2026-06-08.^27^ Supplementary information: ^1^H NMR and ^13^C NMR spectra, GCMS, HR-MS, gel permeation (GPC) HPLC; supplementary computational data and coordinates; additional chiroptical, photophysical and OLED data. See DOI: https://doi.org/10.1039/d6sc04906k.
