# Supplementary material for: Tuning chiroptical properties and device hyperfluorescence efficiency via acceptor modification in an intramolecularly sensitised TADF dendrimer
Source: Chem Sci. 2026 Sep 3;17(41):19987–97. doi: 10.1039/d6sc04906k (PMC13552525; doi:10.1039/d6sc04906k)
Supplement: Supplementary file 1 [file SC-017-D6SC04906K-s002.pdf]

Supplementary Information (SI) for Chemical Science.
This journal is © The Royal Society of Chemistry 2026

Tuning Chiroptical Properties and Device Hyperfluorescence Efficiency via Acceptor Modification in an Intramolecularly Sensitised TADF Dendrimer

Mahni Fatahi[a], Yan Xu[a], Dongyang Chen[a,b], Praveen Choudhary[a], Jhon Sebastian Oviedo Ortiz[c], Jasmin Seibert[d], Stefan Bräse[d,e], Jeanne Crassous[c], Xiao-Hong Zhang*[b] and Eli Zysman-Colman*[a]*

[a]Organic Semiconductor Centre, EaStCHEM School of Chemistry, University of St Andrews, St Andrews, UK, KY16 9ST. E-mail: eli.zysman-colman@st-andrews.ac.uk

[b]Institute of Functional Nano & Soft Materials (FUNSOM) Joint International Research Laboratory of Carbon-Based Functional Materials and Devices Soochow University Suzhou, Jiangsu 21523, P. R. China. E-mail: xiaohong_zhang@suda.edu.cn

[c]University of Rennes, CNRS, ISCR (Institut des Sciences Chimiques de Rennes) - UMR 6226, F-35000 Rennes, France. E-mail: jeanne.crassous@univ-rennes.fr

[d]Institute of Organic Chemistry (IOC), Karlsruhe Institute of Technology (KIT), Kaiserstraße 12, 76131 Karlsruhe, Germany

[e]Institute of Biological and Chemical Systems (IBCS-FMS), Karlsruhe Institute of Technology (KIT), Kaiserstraße 12, 76131 Karlsruhe, Germany

Table of Contents

General Synthetic Procedures S2

Quantum chemical calculations S8

Photophysical measurements S8

Chiroptical measurements. S10

Electrochemistry measurements S10

Fitting of time-resolved photoluminescence measurements S10

Kinetics rate constant calculations S11

OLED Fabrication and Characterisation S11

Experimental Section

General Synthetic Procedures. The following starting materials were synthesised according to literature procedures: **3-bromo-9-(*tert*-butyldimethylsilyl)-9*H*-carbazole**,[1] **CzP**,[2] and **DCzBDFCN**.[3] All other reagents and solvents were obtained from commercial sources and used as received. Air-sensitive reactions were performed under a nitrogen atmosphere using Schlenk techniques; no special precautions were taken to exclude air or moisture during work-up and crystallisation. Anhydrous toluene and anhydrous THF were obtained from an MBraun SPS5 solvent purification system. Flash column chromatography was carried out using silica gel (Silia-P from Silicycle, 60 Å, 40-63 µm). Flash column chromatography was carried out using a Teledyne ISCO CombiFlash® NextGen 300+ system with RediSep® Normal-Phase Silica columns in sizes, ranging from 24 to 80 g of silica packing. Analytical thin-layer chromatography (TLC) was performed on silica plates with aluminium backings (250 µm, F-254 indicator). TLC visualisation was accomplished using a 254/365 nm UV lamp. HPLC analysis was conducted on a Shimadzu LC-40 HPLC system. GPC-HPLC was performed using a Shim-pack GPC-803 column, and enantiopure CzP was separated by semi-preparative HPLC on the Puriflash™ 4125 system from Interchim. A (YMC Chiral ART Amylose-SA©, 250 × 30 mm I.D., S-10 µm) was used as the stationary phase. A linear gradient of *n*-hexane/10% *i*-propanol, both supplemented with 0.1% diethylamine ($HNEt_2$), at a flow rate of 20 mL/min served as the mobile phase: t_R(S_p-(Czp) = 19-24 min, t_R(R_p-(Czp) = 32-41 min. The enantiomeric excess was analysed using an AGILENT HPLC 1100 series system with a G1322A degasser, a G1211A pump, a G1313A autosampler, a G1316A column oven, and a G1315B diode-array detector. An Amylose-SA column (250 × 4.6 mm, 5 µm particle size) with HPLC-grade n-hexane/10% i-propanol as the mobile phase and a flow rate of 1 mL/min was used. The absolute configuration was assigned following our prior study.[2] ^{1}H and ^{13}C NMR spectra were recorded on a Bruker Advance spectrometer (500 MHz for ^{1}H, 125 MHz for ^{13}C). The following abbreviations have been used for multiplicity assignments: “s” for singlet, “d” for doublet, “dd” for doublet of doublets, “td” for triplet of doublets and “m” for multiplet. ^{1}H and ^{13}C NMR spectra were referenced to residual solvent peaks with respect to TMS (δ = 0 ppm). Melting points were measured using open-ended capillaries on an Electrothermal 1101D Mel-Temp apparatus and are uncorrected. High-resolution mass spectrometry (HRMS) was performed on a MALDI-TOF/TOF (Bruker Daltonics) at the Mass Spectrometry facility of the University of Glasgow. FT-IR spectra were recorded on a Jasco FT/IR-6100 type A spectrometer.

1^9-(9*H*-carbazol-3-yl)-1^9*H*-1(1,4)-carbazola-4(1,4)-benzenacyclohexaphane (**CzP-Cz**)

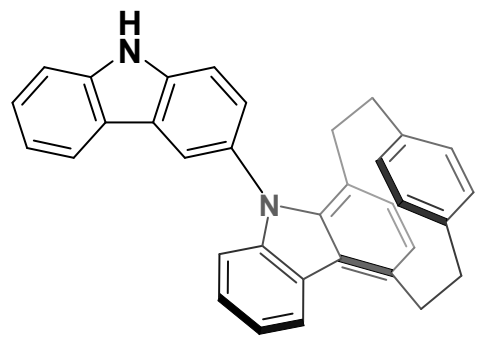

To a Schlenk flask purged with nitrogen 3-bromo-9-(*tert*-butyldimethylsilyl)-9*H*-carbazole (541 mg, 1.50 mmol, 1.0 equiv.), **(*rac*)-** 1^9*H*-1(1,4)-carbazola-4(1,4)-benzenacyclohexaphane (**CzP**) (445 mg, 1.50 mmol, 1.0 equiv.), NaO^{t}Bu (288 mg, 3.00 mmol, 2.0 equiv.), XPhos (107 mg, 0.23 mmol, 0.15 equiv.), and $Pd_2(dba)_3$ (137 mg, 0.15 mmol, 0.10 equiv.) were added, and then three evacuation (30 sec)/ refill (N_2) cycles were performed. Anhydrous toluene (60 mL) was added, and the solution was stirred at 110 °C for 17 h. The mixture was cooled and then filtered through a celite plug, rinsing with toluene (80 mL). The solvent was removed under reduced pressure. The mixture was purified by flash chromatography (EtOAc/PE 1/99; **R_f**: 0.60 (PE on silica gel). The product, 1^9-(9-(*tert*-butyldimethylsilyl)-9*H*-carbazol-3-yl)-1^9*H*-1(1,4)-carbazola-4(1,4)-benzenacyclohexaphane, was then dissolved in anhydrous THF (40 mL) and tetrabutylammonium fluoride (884 mg, 3.38 mmol, 3.0 equiv.) and the solution was stirred at room temperature for 17 h. The reaction mixture was quenched with water (150 mL) and filtered to give a colourless solid. The solid was washed with cold methanol (100 mL) to give the desired product as a colourless solid. **Yield**: 69% (over two steps) (0.47 g). **R_f**: 0.26 (EtOAc/Hex 1:9 on silica gel). **Mp**: 272-275 °C. **^{1}H NMR (500 MHz, DMSO, 100 °C) δ (ppm)**: 10.78 (s, 1H), 7.72 (d, *J* = 7.7 Hz, 3H), 7.27 (d, *J* = 8.1 Hz, 1H), 7.13 (d, *J* = 8.2 Hz, 1H), 7.00 (t, *J* = 7.7 Hz, 1H), 6.87 (tt, *J* = 14.5, 7.1 Hz, 4H), 6.74 (t, *J* = 7.6 Hz, 1H), 6.15 (q, *J* = 7.5 Hz, 2H), 6.06 (dd, *J* = 7.7, 1.9 Hz, 1H), 5.90 (dd, *J* = 7.8, 1.9 Hz, 1H), 5.64 (d, *J* = 7.6 Hz, 1H), 4.97 (dd, *J* = 7.7, 1.9 Hz, 1H), 3.59 (t, *J* = 10.2 Hz, 1H), 2.75 – 2.64 (m, 2H), 2.64 – 2.52 (m, 1H), 2.30 (t, *J* = 11.4 Hz, 1H), 2.14 (ddd, *J* = 13.3, 10.2, 6.6 Hz, 1H), 2.00 (d, *J* = 1.7 Hz, 1H), 1.91 (ddd, *J* = 12.4, 9.7, 6.6 Hz, 1H). **^{13}C NMR (126 MHz, DMSO, 100 °C) δ (ppm)**: 141.29, 141.12, 140.30, 138.42, 137.23, 136.63, 134.64, 132.57, 130.97, 130.73, 129.61, 126.29, 125.56, 125.52, 125.28, 125.12, 124.21, 124.03, 123.26, 122.77, 122.02, 121.42, 120.12, 119.34, 118.32, 118.11, 111.14, 110.70, 109.18, 33.98, 32.46, 32.39, 31.83. **HR-MS (ESI-MS) Calculated**: (**$C_{34}H_{26}N_2Na$**) 485.1988 **Found**: 4485.1987.

CzPBN-CzBN

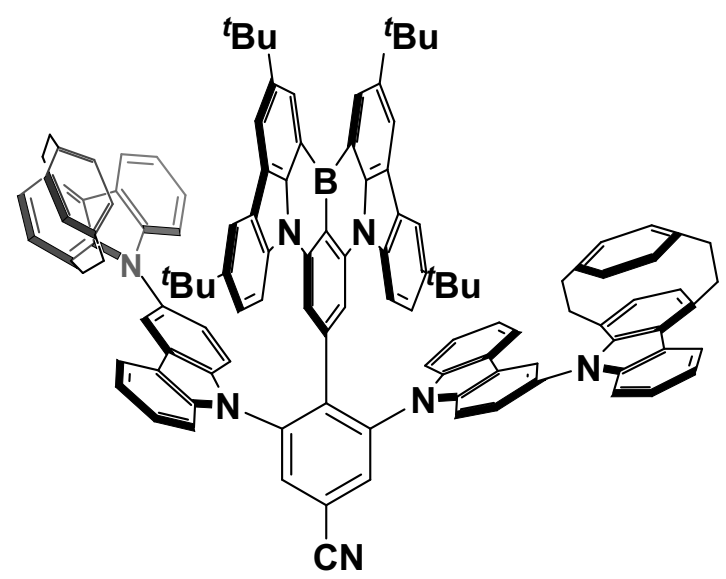

To a Schlenk flask purged with nitrogen, **DCzBDFCN** (250 mg, 0.32 mmol, 1.0 equiv.), **(*rac*)-CzP-Cz** (305 mg, 0.66 mmol, 2.1 equiv.), and Cs_2CO_3 (419 mg, 1.29 mmol, 4.0 equiv.) were added, and then three evacuation (30 sec)/ refill (N_2) cycles were performed. Anhydrous DMF (12 mL) was added, and the mixture was heated to 150 °C and stirred for 17 h. After complete consumption of **DCzBDFCN** (monitored by TLC $\mathbf{R_f}$ (**DCzBDFCN**): 0.5 DCM/PE 1:2), the reaction was quenched with water (50 mL) and extracted with DCM (3 × 50 mL). The combined organic layers were dried over $MgSO_4$, and the solvent was removed under reduced pressure. The crude mixture was purified by flash chromatography (EtOAc/PE 15/85), followed by gel permeation chromatography (Bio-beads S-X3 as the stationary phase) using THF as the eluent. The desired compound was obtained as a yellow solid. **Yield**: 28% (150 mg). $\mathbf{R_f}$: 0.51 (EtOAc/PE 20:80 on silica gel). **Mp**: decomposition above 400 °C. **^{1}H NMR (500 MHz, DMF, 100 °C) δ (ppm)**: 9.33 – 8.89 (m, 3H), 8.92 – 8.73 (m, 5H), 8.47 (s, 2H), 8.39 (s, 2H), 8.31 (dd, *J* = 13.9, 8.2 Hz, 2H), 7.95 (dt, *J* = 26.6, 7.6 Hz, 6H), 7.84 (s, 2H), 7.70 (s, 2H), 7.54 (s, 2H), 7.42 – 7.29 (m, 6H), 7.21 – 6.97 (m, 6H), 6.30 (s, 4H), 5.83 (s, 2H), 5.60 (d, *J* = 7.7 Hz, 2H), 5.16 (d, *J* = 7.5 Hz, 2H), 3.88 (t, *J* = 10.2 Hz, 2H), 1.82 (s, 4H), 1.73 (s, 10H), 1.57 (s, 9H), 1.51 (d, *J* = 13.0 Hz, 18H), 1.41 (s, 9H). Due to significant steric congestion and the presence of multiple conformers in solution, the ^{13}C NMR spectrum exhibited signal overlap and weak signal-to-noise, precluding unambiguous spectral interpretation and peak assignment. **HR-MS (MALDI-MS) Calculated**: ($\mathbf{C_{121}H_{101}BN_7}$) 1663.82 **Found**: 1664.24. **FT-IR:** $\tilde{\nu}$ **=** 2245 cm^{-1} (C≡N, benzonitrile). **HPLC-GPC:** 99.9% pure, retention time 10.916 min in 100% THF.

CzPBA-CzBN

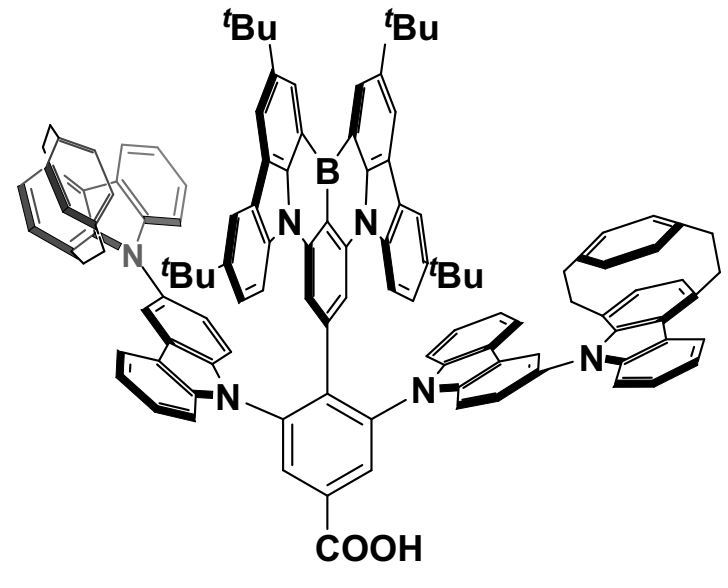

The product was obtained as a byproduct following the same procedure as for **CzPN-CzBN**. The crude reaction was purified by flash chromatography (EtOAc/DCM 2/98), followed by gel permeation chromatography (Bio-beads S-X3 as the stationary phase) using THF as the eluent. The desired compound was obtained as a yellow solid. **Yield**: 14% (75 mg). $\mathbf{R_f}$: 0.61

(EtOAc/DCM 2:98 on silica gel). **Mp**: decomposition above 330 °C. **^{1}H NMR (500 MHz, DMF, 100 °C) δ (ppm)**: 9.14 (dd, *J* = 50.8, 14.0 Hz, 3H), 8.91 (d, *J* = 4.8 Hz, 2H), 8.80 (d, *J* = 43.3 Hz, 2H), 8.58 – 8.35 (m, 3H), 8.27 – 8.12 (m, 6H), 7.93 – 7.72 (m, 6H), 7.66 (s, 2H), 7.48 – 7.26 (m, 8H), 7.10 (s, 4H), 6.33 (d, *J* = 36.1 Hz, 6H), 5.84 (s, 2H), 5.60 (d, *J* = 7.6 Hz, 2H), 5.16 (d, *J* = 7.5 Hz, 2H), 3.88 (t, *J* = 10.5 Hz, 2H), 1.80 (s, 6H), 1.72 (s, 8H), 1.56 (s, 9H), 1.49 (d, *J* = 12.9 Hz, 18H), 1.40 (s, 9H). Due to significant steric congestion and the presence of multiple conformers in solution, the ^{13}C NMR spectrum exhibited signal overlap and weak signal-to-noise, precluding unambiguous spectral interpretation and peak assignment. **HR-MS (ESI-MS) Calculated**: $[M+Cl]^-$ (**$C_{121}H_{101}BClN_6O_2^-$**) 1716.7814 **Found**: 1716.7791; **Calculated**: $[M+CHOO]^-$ (**$C_{122}H_{102}BN_6O_4^-$**) 1726.8104 **Found**: 1726.8185; **Calculated**: $[M+CH_3COO]^-$ (**$C_{121}H_{101}BClN_6O_2^-$**) 1740.8260 **Found**: 1740.8101. **FT-IR**: $\tilde{\nu}$ **=** 1669 cm^{-1} (C=O, benzoic acid). **HPLC-GPC:** 99.4% pure, retention time 11.119 min in 100% THF.

(*R*$_p$)-CzP-Cz

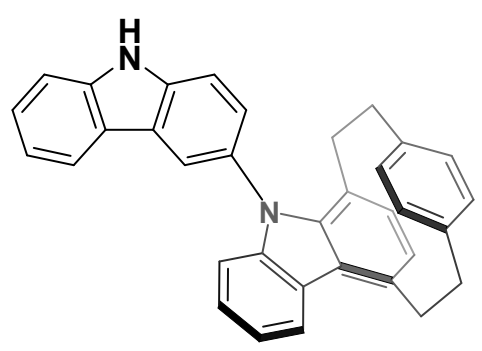

The same experimental procedure as for **Czp-Cz** was followed, except that **(*R*$_p$)-CzP** (100 mg, 0.34 mmol, 1.0 equiv.) was used as the enantiopure starting material. The experimental data match those for **CzP-Cz**. **Yield**: 64% (over both steps) (0.10 g). **R*$_f$***: 0.26 (EtOAc/Hex 10/90 on silica gel). **Mp**: 273-279 °C. **^{1}H NMR (500 MHz, DMSO, 100 °C) δ (ppm)**: 10.81 (s, 1H), 7.73 (d, *J* = 7.8 Hz, 3H), 7.28 (d, *J* = 5.1 Hz, 1H), 7.14 (d, *J* = 8.2 Hz, 1H), 7.01 (t, *J* = 7.8 Hz, 1H), 6.88 (tt, *J* = 14.7, 7.4 Hz, 4H), 6.75 (t, *J* = 7.5 Hz, 1H), 6.16 (q, *J* = 7.5 Hz, 2H), 6.08 (d, *J* = 7.7 Hz, 1H), 5.91 (d, *J* = 7.7 Hz, 1H), 5.65 (d, *J* = 7.6 Hz, 1H), 4.98 (d, *J* = 7.6 Hz, 1H), 3.61 (t, *J* = 10.2 Hz, 1H), 2.69 (t, *J* = 10.0 Hz, 2H), 2.58 (q, *J* = 10.4, 9.4 Hz, 1H), 2.32 (t, *J* = 11.5 Hz, 1H), 2.21 – 2.10 (m, 1H), 2.02 (s, 1H), 1.93 (q, *J* = 9.9 Hz, 1H). **^{13}C NMR (126 MHz, DMSO, 100 °C)) δ (ppm)**: 141.28, 141.11, 140.29, 138.41, 137.22, 136.61, 134.62, 132.56, 130.95, 130.72, 129.59, 126.28, 125.55, 125.50, 125.27, 125.11, 124.20, 124.02, 123.24, 122.75, 122.00, 121.40, 120.10, 119.32, 118.29, 118.09, 111.12, 110.68, 109.17, 33.97, 32.45, 32.38, 31.81.

(*S*$_p$)-CzP-Cz

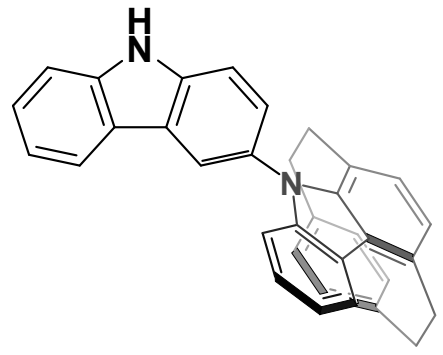

The same experimental procedure as for **Czp-Cz** was followed, except that **(*S*$_p$)-CzP** (100 mg, 0.34 mmol, 1.0 equiv.) was used as the enantiopure starting material. The experimental data match the one for **CzP-Cz**. **Yield**: 62% (over both steps) (0.08 g). **R*$_f$***: 0.26 (EtOAc/Hex 10/90 on silica gel). **Mp**: 273-279 °C. **^{1}H NMR (500 MHz, DMSO, 100 °C) δ (ppm)**: 0.79 (s, 1H), 7.73 (d, *J* = 7.7 Hz, 3H),

7.28 (d, J = 6.1 Hz, 1H), 7.14 (d, J = 8.2 Hz, 1H), 7.01 (t, J = 7.6 Hz, 1H), 6.97 – 6.79 (m, 4H), 6.75 (t, J = 7.6 Hz, 1H), 6.17 (q, J = 7.5 Hz, 2H), 6.08 (dd, J = 7.7, 1.9 Hz, 1H), 5.91 (dd, J = 7.7, 1.9 Hz, 1H), 5.65 (d, J = 7.6 Hz, 1H), 4.98 (dd, J = 7.7, 1.9 Hz, 1H), 3.61 (t, J = 10.3 Hz, 1H), 2.72 (dd, J = 10.5, 8.7 Hz, 2H), 2.61 – 2.53 (m, 1H), 2.32 (t, J = 11.4 Hz, 1H), 2.16 (ddd, J = 13.4, 10.2, 6.7 Hz, 1H), 2.01 (d, J = 1.5 Hz, 1H), 1.92 (ddd, J = 12.7, 9.7, 6.7 Hz, 1H). **^{13}C NMR (126 MHz, DMSO, 100 °C)) δ (ppm)**: 141.27, 141.10, 140.29, 138.40, 137.23, 136.62, 134.64, 132.57, 130.96, 130.73, 129.59, 126.29, 125.54, 125.28, 125.12, 124.21, 124.02, 123.26, 122.76, 122.01, 121.42, 120.13, 119.34, 118.31, 118.12, 111.14, 110.69, 109.17, 40.02, 39.85, 39.69, 39.52, 39.35, 39.19, 39.02, 33.98, 32.45, 32.39, 31.83.

(R_p)-CzPBN-CzBN

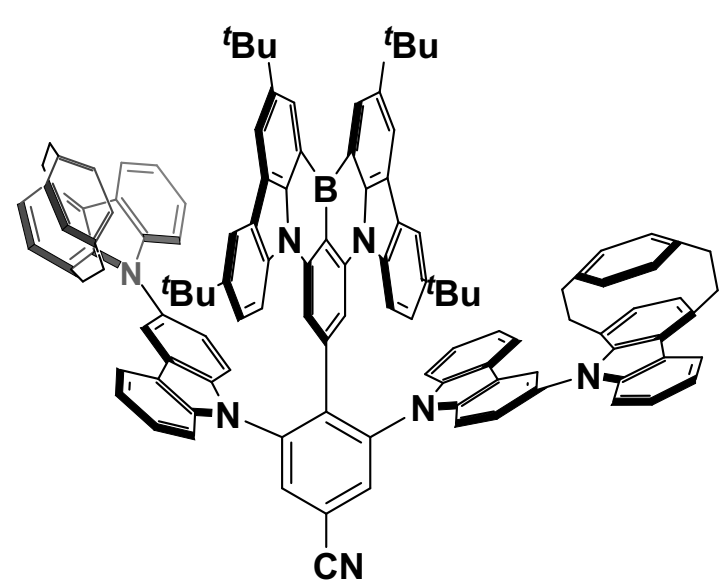

The same experimental procedure as for **CzPBN-CzBN** was followed, except that **(R_p)-CzP-Cz** (67 mg, 0.15 mmol, 2.05 equiv.) was used as the enantiopure starting material. The experimental data match those for **CzPBN-CzBN**. **Yield**: 25% (30 mg). **R_f**: 0.50 (EtOAc/PE 20:80 on silica gel). **Mp**: decomposition above 400 °C. **FT-IR**: $\tilde{\nu}$ = 2231 cm^{-1} (C≡N, benzonitrile). **^{1}H NMR (500 MHz, DMSO, 100 °C) δ (ppm)**: 9.24 – 9.04 (m, 3H), 8.95 – 8.76 (m, 5H), 8.47 (s, 2H), 8.40 (s, 2H), 8.31 (dd, J = 13.9, 8.3 Hz, 2H), 8.02 – 7.95 (m, 6H), 7.92 (t, J = 7.6 Hz, 2H), 7.84 (s, 2H), 7.70 (s, 2H), 7.55 (s, 2H), 7.42 – 7.31 (m, 6H), 7.21 – 6.98 (m, 6H), 6.30 (s, 4H), 5.83 (s, 2H), 5.60 (d, J = 7.7 Hz, 2H), 5.16 (d, J = 7.7 Hz, 2H), 3.88 (t, J = 10.3 Hz, 2H), 1.82 (s, 4H), 1.73 (s, 10H), 1.57 (s, 9H), 1.51 (d, J = 13.0 Hz, 18H), 1.41 (s, 9H). **HPLC-GPC:** 98.5% pure, retention time 10.357 min in 100% THF.

(S_p)-CzPBN-CzBN

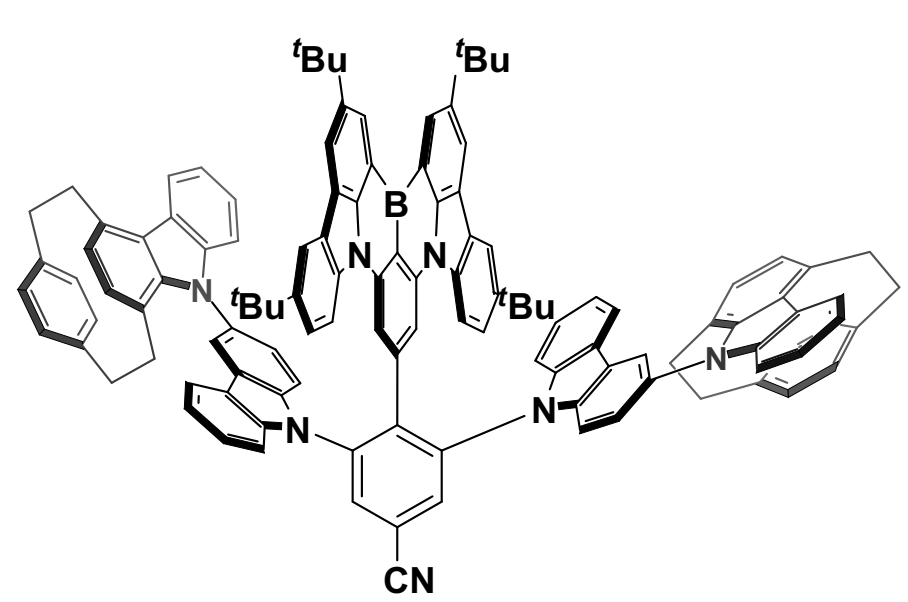

The same experimental procedure as for **CzPBN-CzBN** was followed, except that **(S_p)-CzP-Cz** (67 mg, 0.15 mmol, 2.05 equiv.) was used as the enantiopure starting material. The experimental data match those for **CzPBN-CzBN**. **Yield**: 15% (18 mg). **R_f**: 0.51 (EtOAc/PE 20:80 on silica gel). **Mp**: decomposition above 400 °C. **FT-IR**: $\tilde{\nu}$ = 2240 cm^{-1} (C≡N, benzonitrile). **^{1}H NMR (500 MHz, DMSO, 100 °C) δ (ppm)**: 9.12 (t, J = 30.7 Hz, 3H), 8.95 – 8.57 (m, 2H), 8.47 (d, J = 8.4 Hz, 2H), 8.39 (s, 2H), 8.23 (s, 2H), 7.84 (s, 3H), 7.69 (s, 2H), 7.54 (d, J = 8.5 Hz, 2H), 7.47 (s, 2H),

7.38 – 7.23 (m, 6H), 7.16 – 6.94 (m, 6H), 6.33 (s, 4H), 5.88 (d, *J* = 50.4 Hz, 2H), 5.72 – 5.52 (m, 2H), 5.18 (dd, *J* = 20.0, 7.7 Hz, 2H), 3.90 (d, *J* = 12.7 Hz, 1H), 1.79 – 1.72 (m, 7H), 1.68 (s, 4H), 1.49 (s, 7H), 1.41 (s, 6H), 1.34 (s, 11H). **HPLC-GPC:** 97.4% pure, retention time 10.636 min in 100% THF.

(*R*p)-CzPBA-CzBN

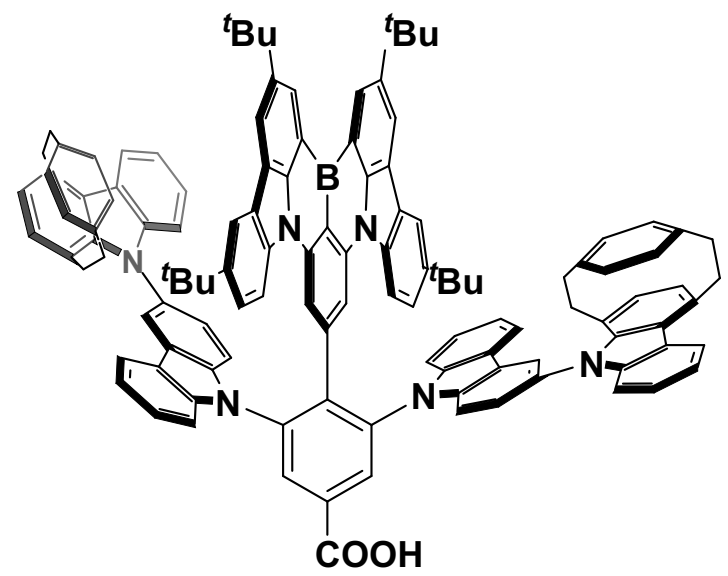

The same experimental procedure as for **CzPBA-CzBN** was followed, except that **(*R*p)-CzP-Cz** (67 mg, 0.15 mmol, 2.05 equiv.) was used as the enantiopure starting material. The experimental data match those for **CzPBA-CzBN**. **Yield**: 8% (10 mg). **R*f***: 0.61 (EtOAc/DCM 2:98 on silica gel). **Mp**: decomposition above 330 °C. **FT-IR**: $\tilde{\nu}$ **=** 1667 cm^{-1} (C=O, benzoic acid).1667 **^{1}H NMR (500 MHz, DMSO, 100 °C) δ (ppm)**: 9.15 (d, *J* = 36.5 Hz, 3H), 8.96 – 8.75 (m, 6H), 8.59 (s, 2H), 8.39 (s, 2H), 8.27 (d, *J* = 8.3 Hz, 3H), 7.98 (s, 6H), 7.84 (s, 2H), 7.69 (s, 2H), 7.49 (d, *J* = 7.6 Hz, 2H), 7.44 – 7.29 (m, 6H), 7.20 – 6.98 (m, 6H), 6.30 (s, 4H), 5.81 (d, *J* = 17.5 Hz, 2H), 5.61 (d, *J* = 7.5 Hz, 2H), 5.17 (d, *J* = 7.6 Hz, 2H), 3.89 (t, *J* = 10.4 Hz, 3H), 1.78 – 1.71 (m, 10H), 1.68 (s, 4H), 1.46 (d, *J* = 38.3 Hz, 18H), 1.35 (s, 18H). **HPLC-GPC:** 98.6% pure, retention time 10.172 min in 100% THF.

(*S*p)-CzPBA-CzBN

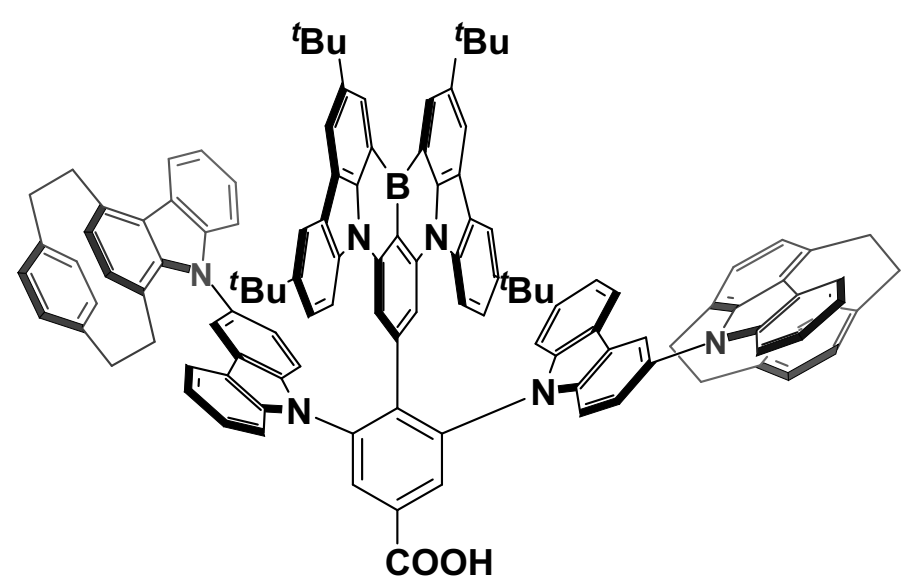

The same experimental procedure as for **CzPBA-CzBN** was followed, except that **(*S*p)-CzP-Cz** (67 mg, 0.15 mmol, 2.05 equiv.) was used as the enantiopure starting material. The experimental data match those for **CzPBA-CzBN**. **Yield**: 21% (25 mg). **R*f***: 0.61 (EtOAc/DCM 2:98 on silica gel). **Mp**: decomposition above 330 °C. **FT-IR**: $\tilde{\nu}$ **=** 1697 cm^{-1} (C=O, benzoic acid). **^{1}H NMR (500 MHz, DMSO, 100 °C) δ (ppm)**: 9.16 (d, *J* = 32.0 Hz, 3H), 8.93 (s, 2H), 8.75 (s, 2H), 8.56 (s, 1H), 8.37 (s, 2H), 8.27 (d, *J* = 8.2 Hz, 2H), 8.21 (s, 2H), 7.99 (s, 6H), 7.90 (d, *J* = 6.4 Hz, 2H), 7.78 (s, 2H), 7.62 (s, 2H), 7.47 – 7.35 (m, 6H), 7.14 (s, 6H), 6.29 (s, 4H), 5.82 (d, *J* = 17.4 Hz, 2H), 5.57 (d, *J* = 7.6 Hz, 2H), 5.16 (s, 2H), 3.87 (s, 2H), 1.73 (s, 10H), 1.68 (s, 4H), 1.50 (s, 18H), 1.43 (s, 9H), 1.34 (s, 9H). . **HPLC-GPC:** 99.5% pure, retention time 10.424 min in 100% THF.

Quantum chemical calculations. Density functional theory (DFT) and TD(A)-DFT calculations were performed using the Gaussian 16 revision C.01 package.[4] The ground-state optimisation was carried out using DFT using the PBE0[5] functional, the 6-31G(d,p) basis set [6] and in conjunction with the D3(BJ)[7] dispersion correction scheme in the gas phase, starting from a structure drawn and optimised using Chem3D.[8] The vertical excited-state calculations were performed using Time-Dependent DFT within the Tamm-Dancoff approximation (TDA-DFT),[9] with the same functional and basis set as those used for the ground-state geometry optimisation in the gas phase. Spin-orbit coupling matrix elements, SOCME, were calculated based on the optimised ground-state geometry using PySOC.[10] The calculations employed the standard implementation of the PySOC methodology, i.e., the Breit–Pauli spin–orbit Hamiltonian within the effective nuclear charge approximation, as described in the original publication. Electronic structure information was obtained from Gaussian 16 TDA-DFT calculations performed at the PBE0/6-31G(d,p) level of theory, and the corresponding Gaussian log and rwf files were used as input for the PySOC calculations. The official PySOC implementation available was used without modification. The molecular orbital distributions were visualised with Gaussview 6.0.[8] Calculations were submitted and processed using the Digichem software package (version 7),[11-12] which incorporates several publicly available software libraries, including: cclib[13] for parsing of result files, VMD[14]/Tachyon[15] for 3D rendering, Matplotlib for drawing of graphs[16], Open Babel[17]/Pybe[18] file interconversion.

Photophysical measurements. Optically dilute solutions at concentrations on the order of 10^{-5} or 10^{-6} M were prepared in spectroscopic-grade toluene for absorption and photoluminescence (PL) spectral analysis. Absorption spectra were recorded at room temperature using a Shimadzu UV-2600 double-beam spectrophotometer with a 1-cm quartz cuvette. Molar absorptivity determination was verified by linear regression analysis of values obtained from at least four independent solutions at concentrations ranging from 3.0×10^{-6} to 1.0×10^{-5} M with absorbance ranging from 0.025 to 0.100.

For emission studies, aerated solutions, steady-state PL and excitation spectra and time-resolved PL measurements were recorded at room temperature using an Edinburgh Instruments FS5 fluorimeter. Samples were excited at 350 nm for steady-state PL measurements and 375 nm for time-resolved PL decays. Degassed solutions were prepared via three freeze-pump-thaw cycles, and spectra were measured using a home-made Schlenk quartz cuvette. Photoluminescence quantum yields (Φ_{PL}) for solutions were determined using the optically dilute method[19] in which four sample solutions with absorbances of ca. 0.10, 0.095, 0.080 and 0.06 at 350 nm were used.

The Beer-Lambert law was found to remain linear over the solution concentrations. For each sample, linearity between absorption and emission intensity was verified through linear regression analysis, with the Pearson regression factor (R^2) for the linear fit of the data set surpassing 0.9. Individual relative quantum yield values were calculated for each solution, and the reported values represent the slope obtained from the linear fit of these results. The Φ_{PL} was determined using the equation $\Phi_{PL} = (\Phi_r * \frac{A_r}{A_s} * \frac{I_s}{I_r} * \frac{n_s^2}{n_r^2})$, where A stands for the absorbance at the excitation wavelength (λ_{exc}: 350 nm), I is the integrated area under the corrected emission curve and n is the refractive index of the solvent with the subscripts "s" and "r" representing sample and reference respectively. Φ_r is the absolute PL quantum yield of the external reference, quinine sulfate (Φ_r = 54.6% in 1 *N* H_2SO_4).[20] The experimental uncertainty in the Φ_{PL} is conservatively estimated at 10%, though we have found that, statistically, we can reproduce Φ_{PL} values to within 3% relative error.

For solid-state film measurements, the 5 wt% doped films of emitters in a host matrix were prepared from solutions, which were prepared as follows: 9.5 mg of host was dissolved in 475 μL of chlorobenzene and to this, 0.5 mg emitter dissolved in 25 μL of chlorobenzene was added. Thin films were then spin-coated onto a sapphire substrate using a spin speed of 1500 rpm for 60 s and annealed at 50 °C for 60 s to obtain a thickness of ~80 nm. An integrating sphere (Edinburgh Instruments FS5, SC30 module) was employed for photoluminescence quantum yield measurements of thin-film samples. The Φ_{PL} of the films was then measured in air and in N_2 by purging the integrating sphere with N_2 for 2 min. The photophysical properties of the film samples were measured using an Edinburgh Instruments FS5 fluorimeter. Time-resolved PL measurements of the thin films were carried out using the multi-channel scaling (MCS) and time-correlated single-photon counting (TCSPC) technique. The samples were excited at 375 nm by a pulsed laser and kept in a vacuum of $<1 \times 10^{-4}$ mbar.

The singlet and triplet state energies in 2-MeTHF glass and in doped film were determined from the peak values of the steady-state PL (SS PL) and delayed emission spectra at 77 K. The singlet-triplet energy gap (ΔE_{ST}) was estimated from the difference in energy of the peaks of these spectra. For SS PL, samples were excited by a xenon lamp emitting at 365 nm (2-MeTHF) and 340 nm (5 wt% in PhCzBCz). For phosphorescence spectra, samples were excited by a xenon flashlamp emitting at 365 nm (2-MeTHF) and 310 nm (20 wt% in PPF) (EI FS5, SC-70). Phosphorescence spectra were measured with a time-gated window of 10-85 ms (2-MeTHF) and 1-8.5 ms (20 wt% in PPF) with a xenon flashlamp operating at 10 Hz (2-MeTHF) and 100 Hz (20 wt% in PPF).

Chiroptical measurements.

ECD spectra of 10^{-6} M solutions of the compounds in toluene were measured with a Jasco J-815 instrument. CPL measurements were performed using a home-built CPL spectrofluoropolarimeter (constructed with the help of the JASCO Company). The samples were excited using a 90° geometry with a 150 W LS-Xenon ozone-free lamp. The concentrations of the samples were measured precisely at ca. 10^{-6} M in toluene with 12 accumulations. Excitation of the samples was performed at 350 nm.

Electrochemistry measurements. Cyclic Voltammetry (CV) analysis was performed using a CH Instruments Electrochemical Analyser potentiostat model 620E at a scan rate of 100 mV/s. Differential pulse voltammetry (DPV) was conducted with an increment potential of 0.004 V and a pulse amplitude, width, and period of 50 mV, 0.05, and 0.5 s, respectively. Samples were prepared as dichloromethane (DCM) solutions and degassed by sparging with DCM-saturated nitrogen gas for 5 minutes before measurements. All measurements were performed using a 0.1 M DCM solution of tetra-*n*-butylammonium hexafluorophosphate ($[^{n}Bu_4N]PF_6$). An Ag/Ag^+ electrode was used as the reference electrode, while a platinum electrode and a platinum wire were used as the working electrode and counter electrode, respectively. The redox potentials are reported relative to a saturated calomel electrode (SCE), with the ferrocenium/ferrocene (Fc/Fc+) couple as the internal standard (0.46 V vs SCE).[21]

Fitting of time-resolved photoluminescence measurements: Time-resolved PL measurements were fitted to a sum of exponentials decay model, with chi-squared (χ^2) values between 1 and 2, using the Fluoracle software. Each component of the decay is assigned a weight, (w_i), which is the contribution of the emission from each component to the total emission.

The average lifetime was then calculated using the following:

- Two exponential decay model:

$$\tau_{AVG} = \tau_1 w_1 + \tau_2 w_2$$

with weights defined as $w_1 = \frac{A1\tau_1}{A1\tau_1 + A2\tau_2}$ and $w_2 = \frac{A2\tau_2}{A1\tau_1 + A2\tau_2}$ where A1 and A2 are the preexponential-factors of each component.

- Three exponential decay model:

$$\tau_{AVG} = \tau_1 w_1 + \tau_2 w_2 + \tau_3 w_3$$

with weights defined as $w_1 = \frac{A1\tau_1}{A1\tau_1 + A2\tau_2 + A3\tau_3}$, $w_2 = \frac{A2\tau_2}{A1\tau_1 + A2\tau_2 + A3\tau_3}$ and $w_3 = \frac{A3\tau_3}{A1\tau_1 + A2\tau_2 + A3\tau_3}$ where A1, A2 and A3 are the preexponential-factors of each component.

Kinetics rate constant calculations

The kinetic constants in this paper are calculated following the literature:[22]

$k_p = 1/\tau_p$, $k_d = 1/\tau_d$

$$k_r^S = k_p \Phi_{PF}$$

$$k_{nr}^S = k_p \frac{\Phi_{PF}}{\Phi_{PLQY}} (1 - \Phi_{PLQY})$$

$$k_{nr}^T = k_d (1 - \frac{\Phi_{DF}}{1 - \Phi_{PF}})$$

$$k_{ISC} = \{[k_p(1 - \Phi_{PF}) - k_d \Phi_{PF}]\Phi_{PLQY} + k_p \Phi_{PF} \Phi_{DF} + [k_p \Phi_{PF}^2 (1 - \Phi_{PLQY}) + k_d \Phi_{DF} \Phi_{PLQY}]\} /(2\Phi_{PF}\Phi_{PLQY})$$

$$k_{RISC} = \frac{\Phi_{PLQY}}{2} \times \frac{\Phi_{PLQY}(1 - \Phi_{PF}) + \Phi_{DF} + \Phi_{PF}(1 - \Phi_{PLQY})}{\Phi_{PF}(1 - \Phi_{PF})}$$

$$K_{eq} = \frac{3k_{RISC} + k_r^T + k_{nr}^T}{3(k_r^S + k_{nr}^S) + k_{ISC}}$$

For a TADF emitter with photoluminescence quantum yield near unity and no phosphorescence contribution,

$$k_r^S K_{eq} = \frac{4k_r^S k_{RISC}}{3k_r^S + 4k_{ISC}}$$

OLED Fabrication and Characterisation

OLEDs were fabricated on multiple-layer indium-tin oxide (ITO)- coated transparent glass substrates. The ITO glass substrates have a thickness of ca. 100 nm and a sheet resistance of ca. 30 Ω per square and were cleaned with optical detergent, deionised water, acetone, and isopropanol successively and then dried in an oven. For solution-processed OLEDs, the ITO substrates were first exposed to UV ozone for 15 minutes. Then, the PEDOT:PSS layer was prepared by spin-coating onto the prepared ITO glass substrates and annealed at 120 °C for 20 min under air. Then,

these glass substrates were transferred into a nitrogen-filled glovebox to prepare the emissive materials layers. The host, TADF sensitiser, and guest were dissolved in chlorobenzene solvent (10 mg/mL), and the mixture was stirred at 50 °C before the spin-coating. The remaining layers were then sequentially fabricated via vacuum evaporation. The EL performance of the devices was measured at room temperature using a PHOTO RESEARCH Spectra Scan PR 655 PHOTOMETER and a KEITHLEY 2400 Source Meter as a constant-current source.

Table **S1**. SOCME values for **CzPBN-MeCzBN** and **CzPBA-MeCzBN**. Calculated at PBE0/6-31G(d,p) level of theory in the gas phase at the optimised ground state geometry.

Singlet state	Triplet state	SOCME (H_{SO} Root sum square) / cm^{-1}	
		**CzPBN-MeCzBN**	**CzPBA-MeCzBN**
S_1	T_1	0.0167	0.0381
S_1	T_2	0.0221	0.0776
S_1	T_3	0.2013	0.1480
S_1	T_4	0.1281	0.1234
S_1	T_5	0.1899	0.1244
S_1	T_6	0.1599	0.1921
S_2	T_1	0.1179	0.0507
S_2	T_2	0.2648	0.02273
S_2	T_3	0.0482	0.1012
S_2	T_4	0.1292	0.1395
S_2	T_5	0.0761	0.0752
S_2	T_6	0.1217	0.1910
S_3	T_1	0.1659	0.1610
S_3	T_2	0.1016	0.0876
S_3	T_3	0.0831	0.0767
S_3	T_4	0.1193	0.1765
S_3	T_5	0.5172	0.5279
S_3	T_6	0.2867	0.3209

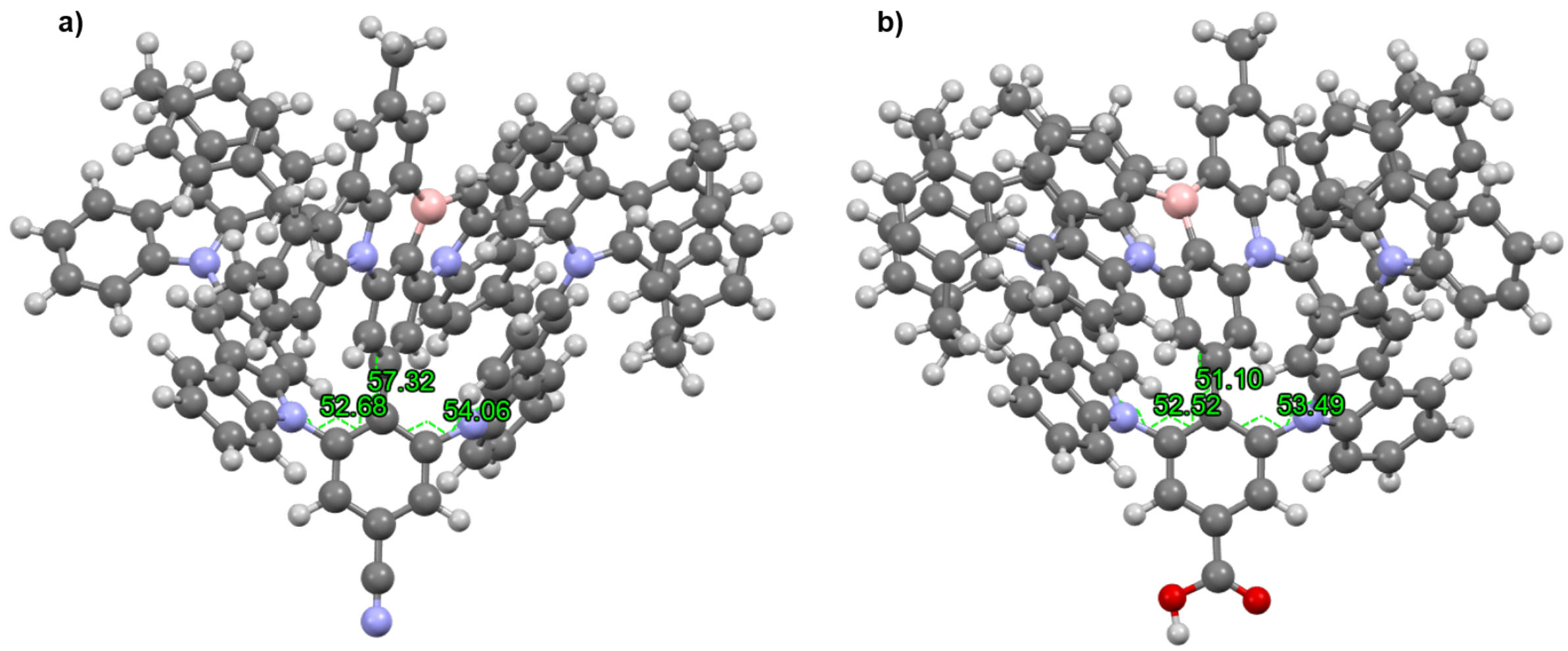

Figure **S1**. Ground-state optimised geometries with key torsion angles ∡ Ph − Cz and ∡ Ph − MeCzBN of a) **CzPBN-MeCzBN** and b) **CzPBA-MeCzBN** calculated at the PBE0/6-31G(d,p) level of theory in the gas phase.

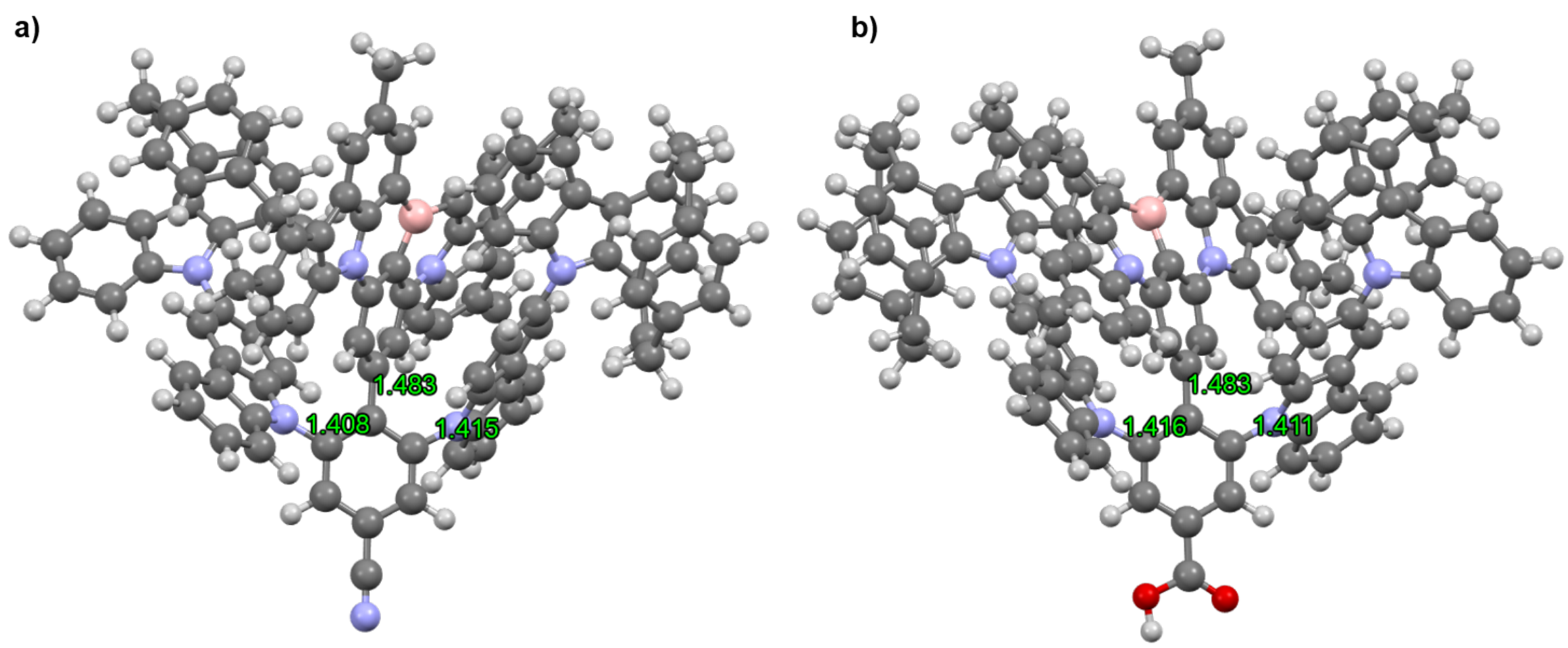

Figure **S2**. Ground-state optimised geometries with key donor-acceptor bond length d-Ph-Cz and d-Ph-MeCzBN of a) **CzPBN-MeCzBN** and b) **CzPBA-MeCzBN** calculated at the PBE0/6-31G(d,p) level of theory in the gas phase.

Table **S2**. Key bond length and torsion angles of ground state optimised structures of CzPBN-MeCzBN and CzPBA-MeCzBN calculated at PBE0/6-31G(d,p) level of theory in the gas phase.

	**CzPBN-MeCzBN**	**CzPBA-MeCzBN**
d-Ph-Cz[a]	1.408/1.415 Å	1.416/1.411 Å
d-Ph-MeCzBN[b]	1.483 Å	1.483 Å
$\measuredangle$ Ph − Cz[c]	52.68°/54.06°	52.52°/53.49°
$\measuredangle$ Ph − MeCzBN[d]	57.32°	51.10°

[a] bond length between central phenyl ring and carbazole nitrogen of the donor dendrons. [b] bond length between central phenyl ring and MeCzBN unit. [c] torsion angle between central phenyl ring and donor dendrons. [d] torsion angle between central phenyl ring and MeCzBN unit.

The calculated electric (μ) and magnetic (m) transition dipole moments between the ground state and the S_1 excited state are 1.17/1.23 D and 0.17/0.16 au for **CzPBN-MeCzBN/CzPBA-MeCzBN**, respectively. The corresponding vector components (x,y,z) are μ = -0.70, 0.94, 0.05 D/ 0.87, 0.14, 0.86 D and m = -0.07, -0.16, -0.01 au/ 0.00, -0.03, -0.15 au for **CzPBN-MeCzBN/CzPBA-MeCzBN**, respectively. In comparison to the molecular geometry, the angle between each dipole moment and the longest axis of the molecule (the x-axis) is $\theta_{\mu,x}$ = 53.51°/44.99° and $\theta_{m,x}$ = 66.82°/89.93°, while the angle between each dipole moment and the xy-plane is $\theta_{\mu,xy}$ = 2.69°/44.27° and $\theta_{m,xy}$ = 1.76°/79.82° for **CzPBN-MeCzBN/CzPBA-MeCzBN**, respectively. In Gaussian-CGS units, in which the magnetic and electric transition dipole moments can be directly compared, the magnitude of each dipole moment is μ = 1.17/1.23 × 10^{-18} esu·cm and m = 1.60/1.44 × 10^{-21} erg·G^{-1}, while the angle between the two dipole moments is $\theta_{\mu,m}$ = 59.68°/45.09° for **CzPBN-MeCzBN/CzPBA-MeCzBN**, respectively. Correspondingly, the cosines of the angles are $\cos(\theta_{\mu,m})$ = 0.50 and 0.71 for **CzPBN-MeCzBN** and **CzPBA-MeCzBN**, respectively, and the dissymmetry factors of the excited state transition are g_{PL} = 0.0027 and 0.0033 for **CzPBN-MeCzBN** and **CzPBA-MeCzBN**, respectively.

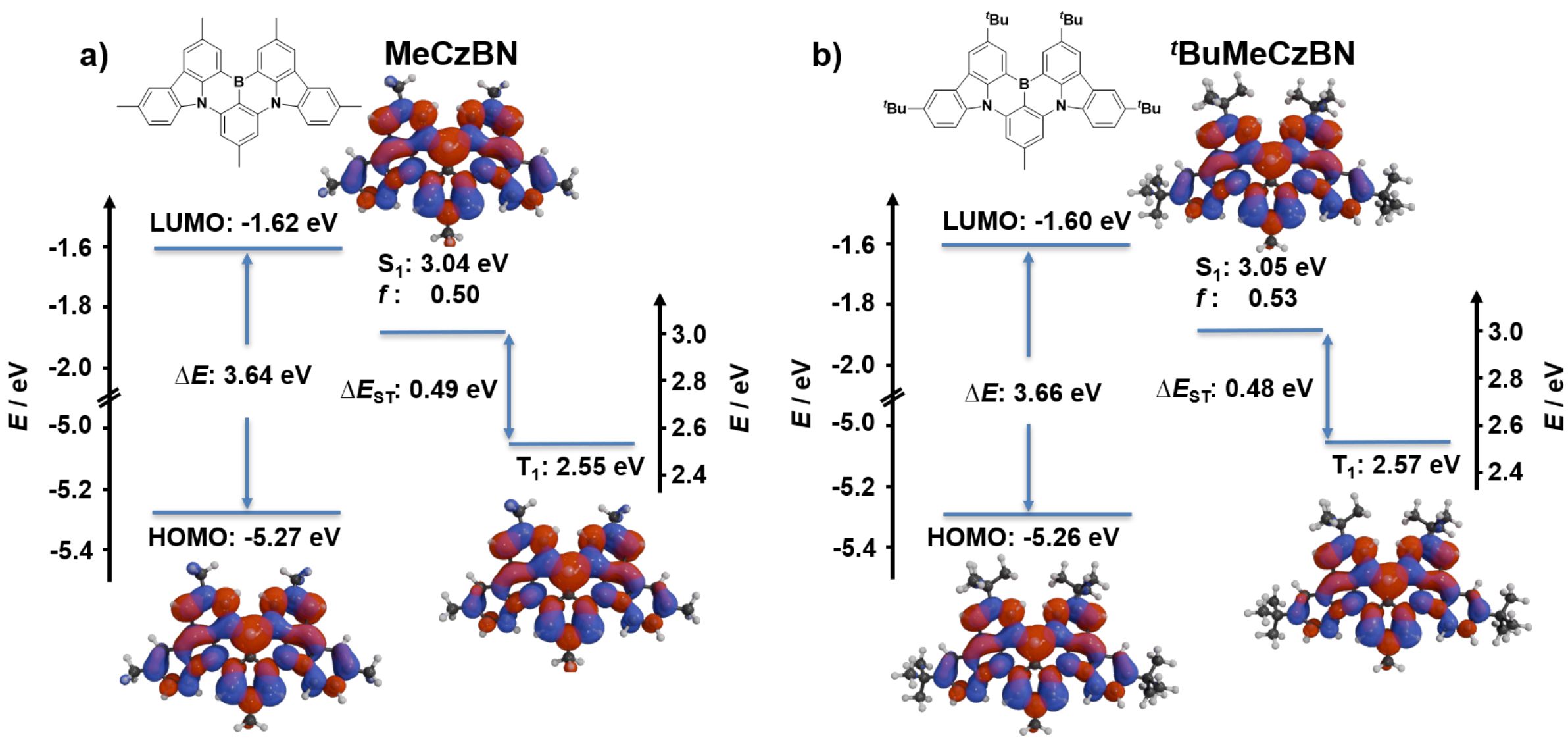

Figure **S3**. Frontier molecular orbitals energies and orbital density distributions (red= LUMO; blue= HOMO; Isovalue = 0.02) and excited-state energies and NTO density plots (red = electron; blue = hole; Isovalue = 0.02) for the S_1 and T_1 states for a) **MeCzBN** and b) ***t*BuMeCzBN**. Calculated at PBE0/6-31G(d,p) level of theory in the gas phase.

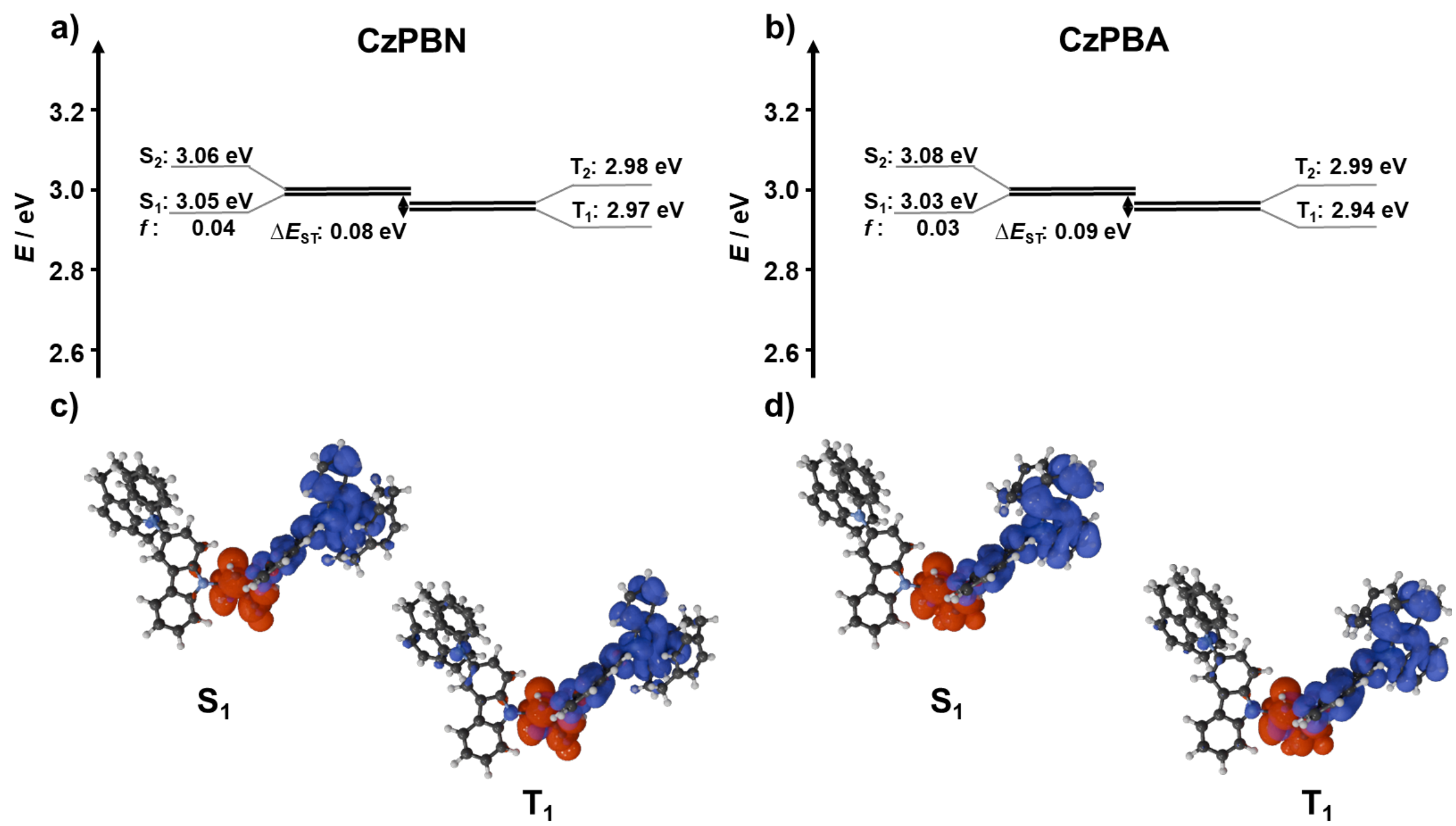

Figure **S4**. Excited-state manifolds for a) **CzPBN** and b) **CzPBA**. NTO density plots for the S_1 and T_1 states of c) **CzPBN-MeCzBN** and d) **CzPBA-MeCzBN** (red = electron; blue = hole; isovalue = 0.02). Calculated at PBE0/6-31G(d,p) level of theory in the gas phase.

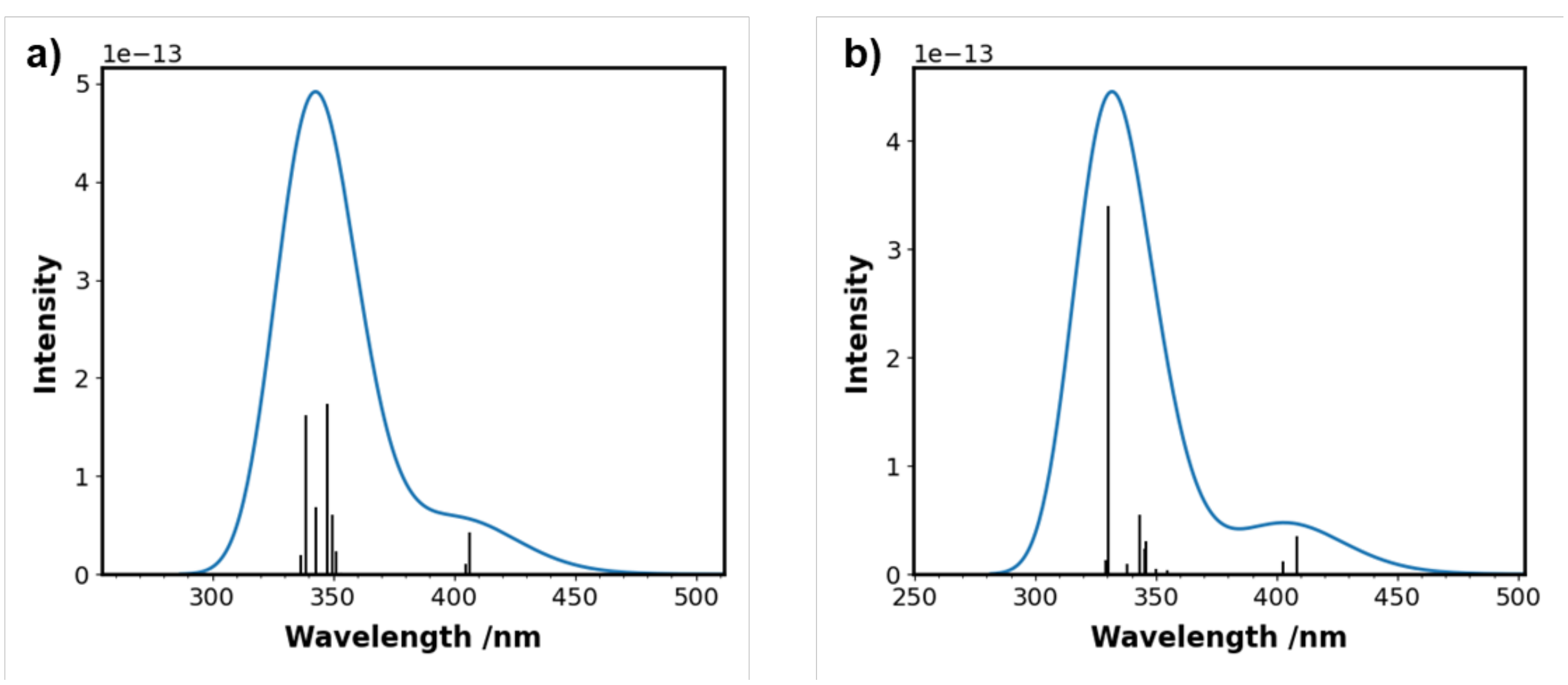

Figure **S5**. Simulated absorption spectrum for a) **CzPBN** and b) **CzPBA**. Calculated at PBE0/6-31G(d,p) level of theory in the gas phase.

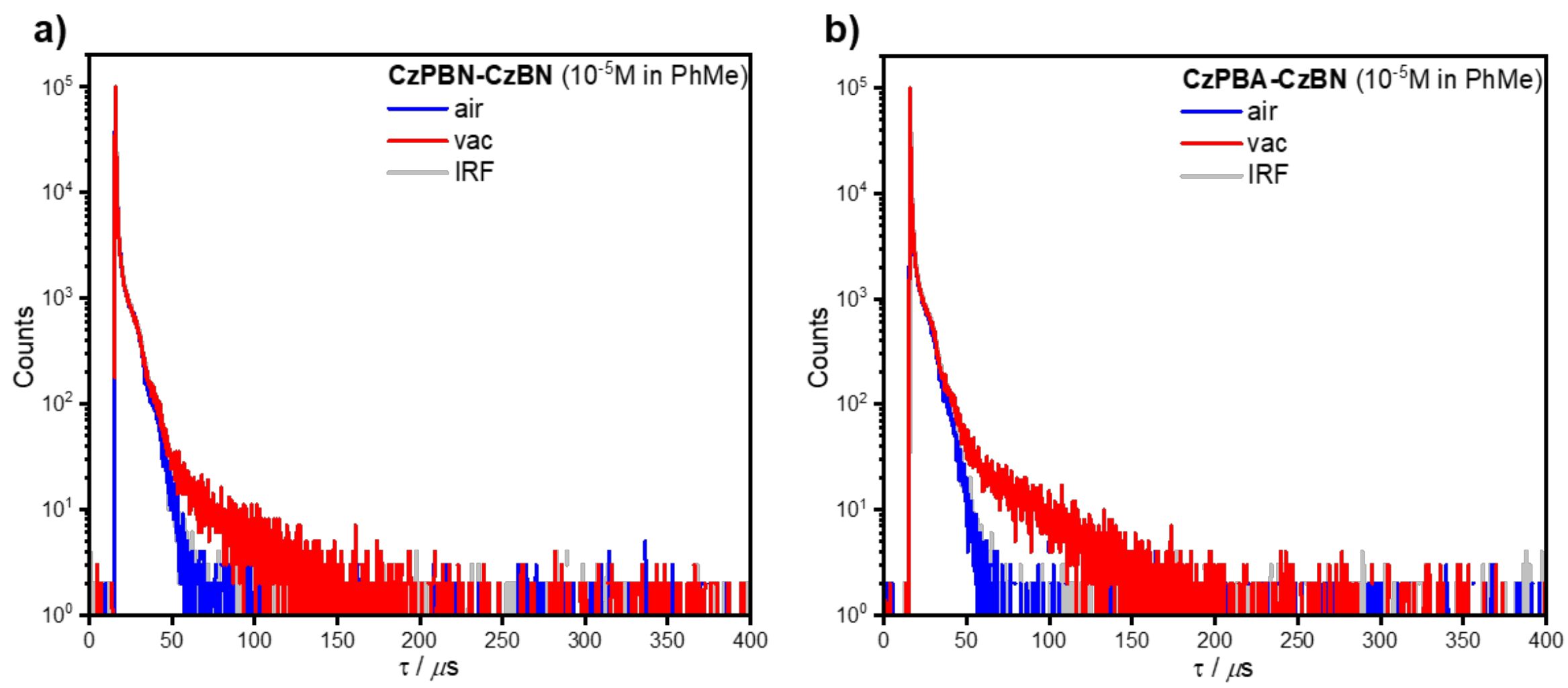

Figure **S6**. TRPL decay for delayed emission in degassed (red) and aerated (blue) toluene solution (10^{-5} M) for a) **CzPBN-CzBN** and b) **CzPBA-CzBN**. (λ_{exc} = 350 nm)

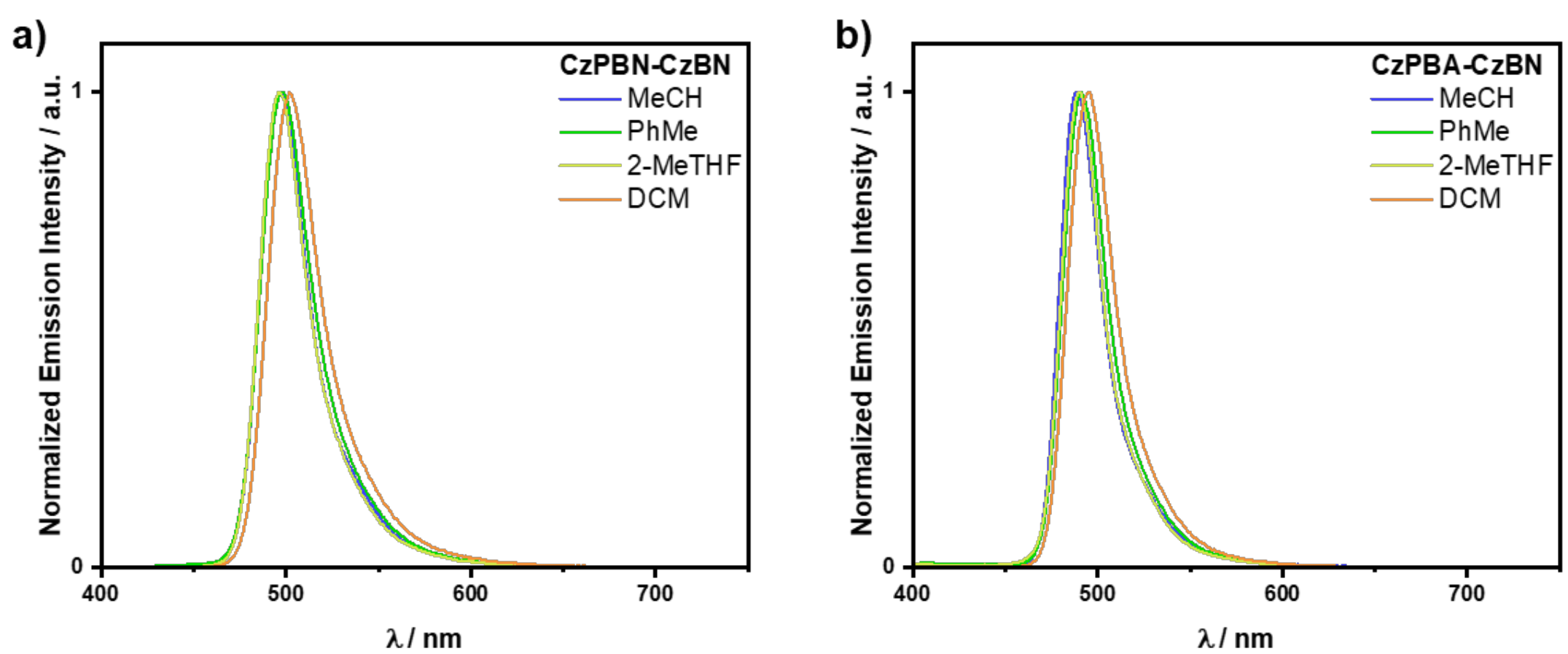

Figure **S7**. Solvatochromism PL study of dilute solution of a) **CzPBN-CzBN** and b) **CZPBA-CzBN**. (λ_{exc} = 350 nm)

Table **S3**. Photophysical data of solvatochromism PL study of **CzPBN-CzBN** and **CzPBA-CzBN**. (λ_{exc} = 350 nm)

Emitter	Solvent	λ_{PL}[e] / nm	FWHM[f] / nm
**CzPBN-CzBN**	MeCH[a]	497	31
	PhMe[b]	499	33
	2-MeTHF[c]	496	31
	DCM[d]	502	34
**CzPBA-CzBN**	MeCH[a]	490	26
	PhMe[b]	491	27
	2-MeTHF[c]	490	26
	DCM[d]	495	30

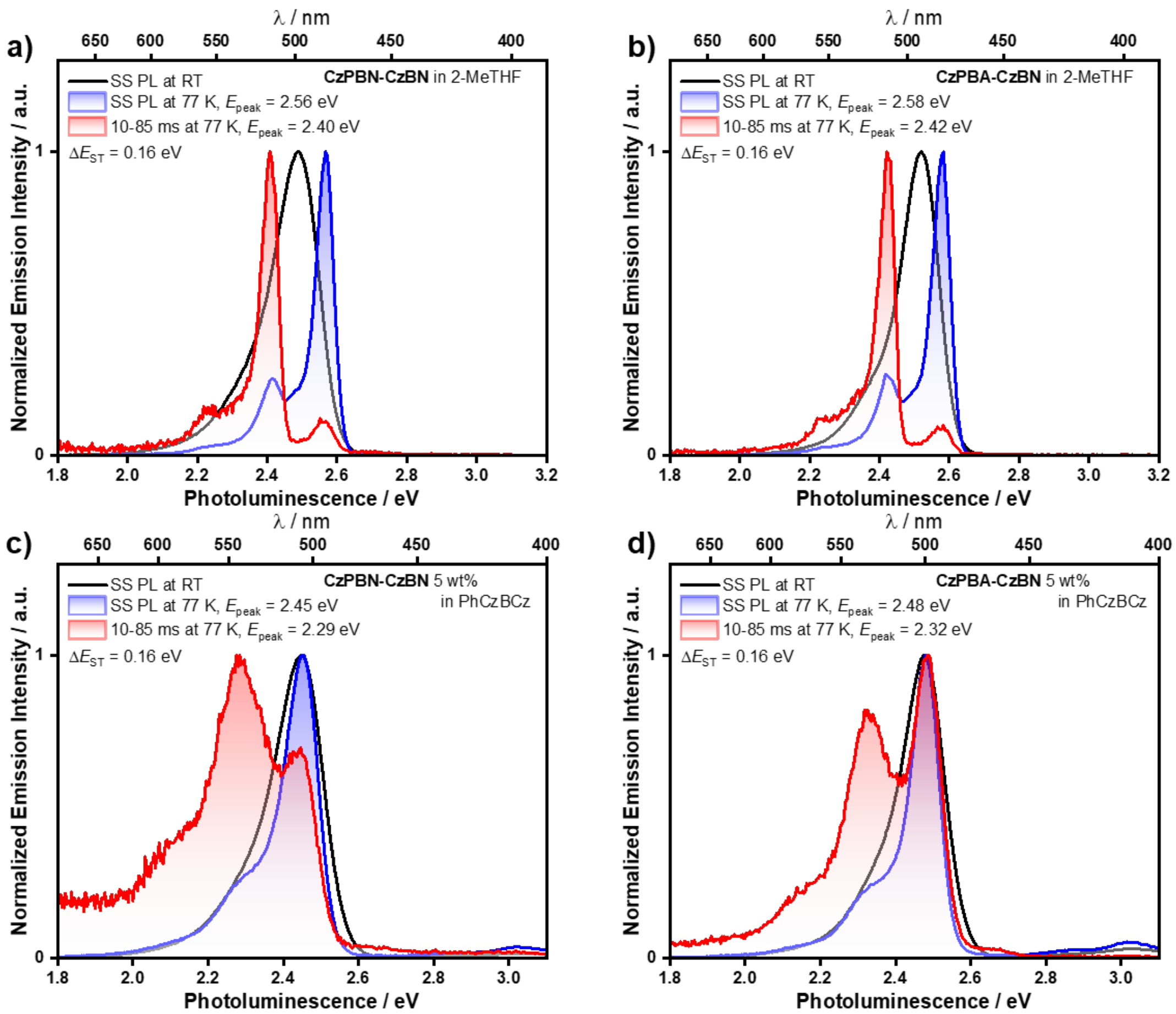

Figure **S8**. SS-PL (black), SS-PL at 77 K (blue) and time-gated emission (10-85 ms) (red) of diluted solutions of a) **CzPBN-CzBN** and b) **CzpBA-CzBN** in 2-MeTHF (λ_{exc} = 350 nm), and of 5 wt% doped films of c) **CzPBN-CzBN** and d) **CzpBA-CzBN** PhCzBCz (λ_{exc} = 340 nm).

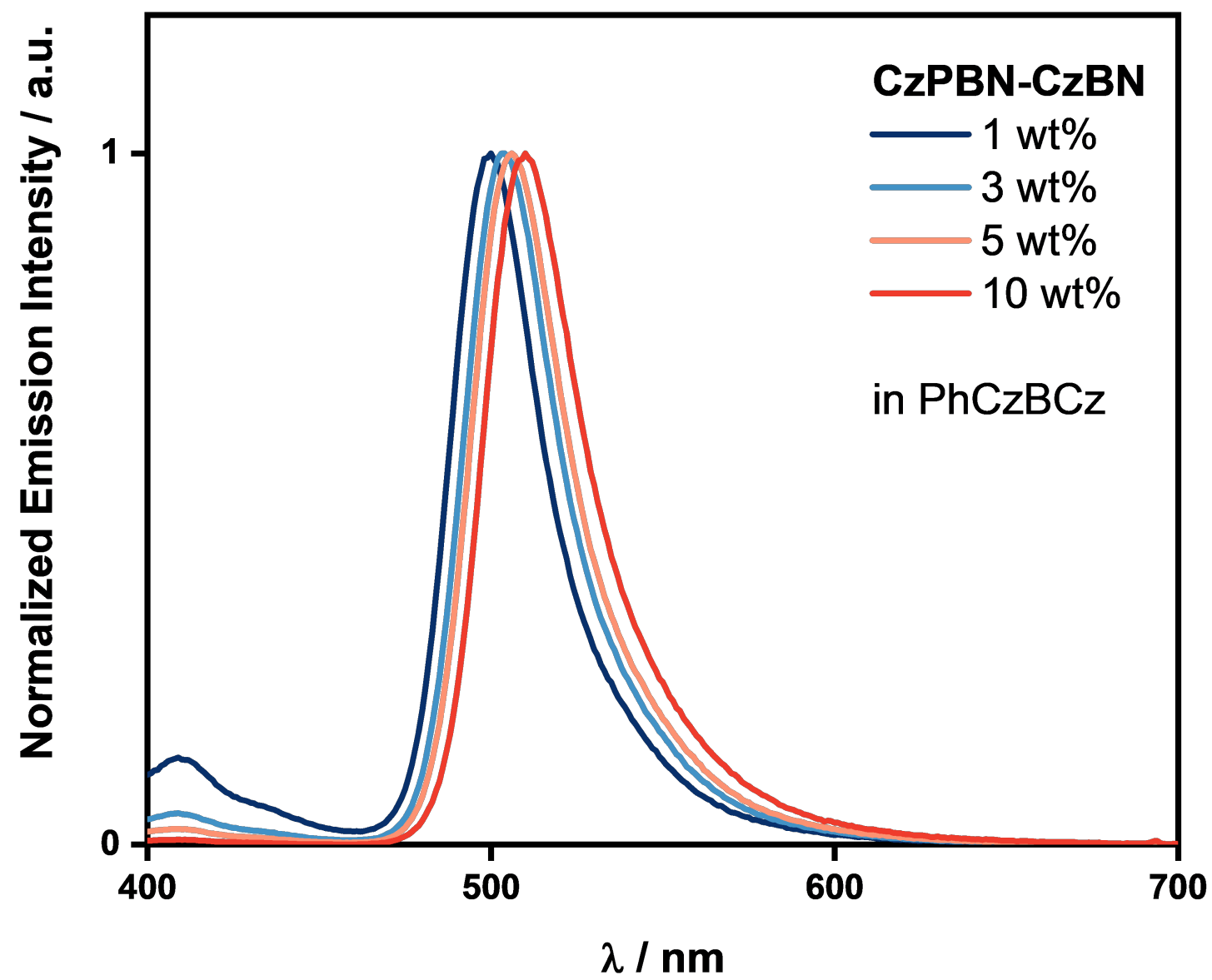

Figure **S9**. SS-PL spectra of doped films of **CzPBN-CzBN** in PhCzBCz at different doping concentrations (λ_{exc} = 340 nm).

Table **S4**. Photophysical data of doped films of **CzPBN-CzBN** in PhCzBCz at different doping concentrations.

**Doping concentration**[a]	**λ_{PL}**[b] **/ nm**	**FWHM**[c] **/ nm**	**Φ_{PL} N_2/air**[d] **/%**
1 wt%	500	32	84/60
3 wt%	504	33	77/60
5 wt%	506	33	76/60
10 wt%	510	35	54/43

[a] maximum of the SS-PL spectrum. [b] full-width at half-maximum. [c] Absolute Φ_{PL} of the thin films were measured using an integrating sphere. Films were measured under an N_2/air atmosphere. (λ_{exc} = 340 nm)

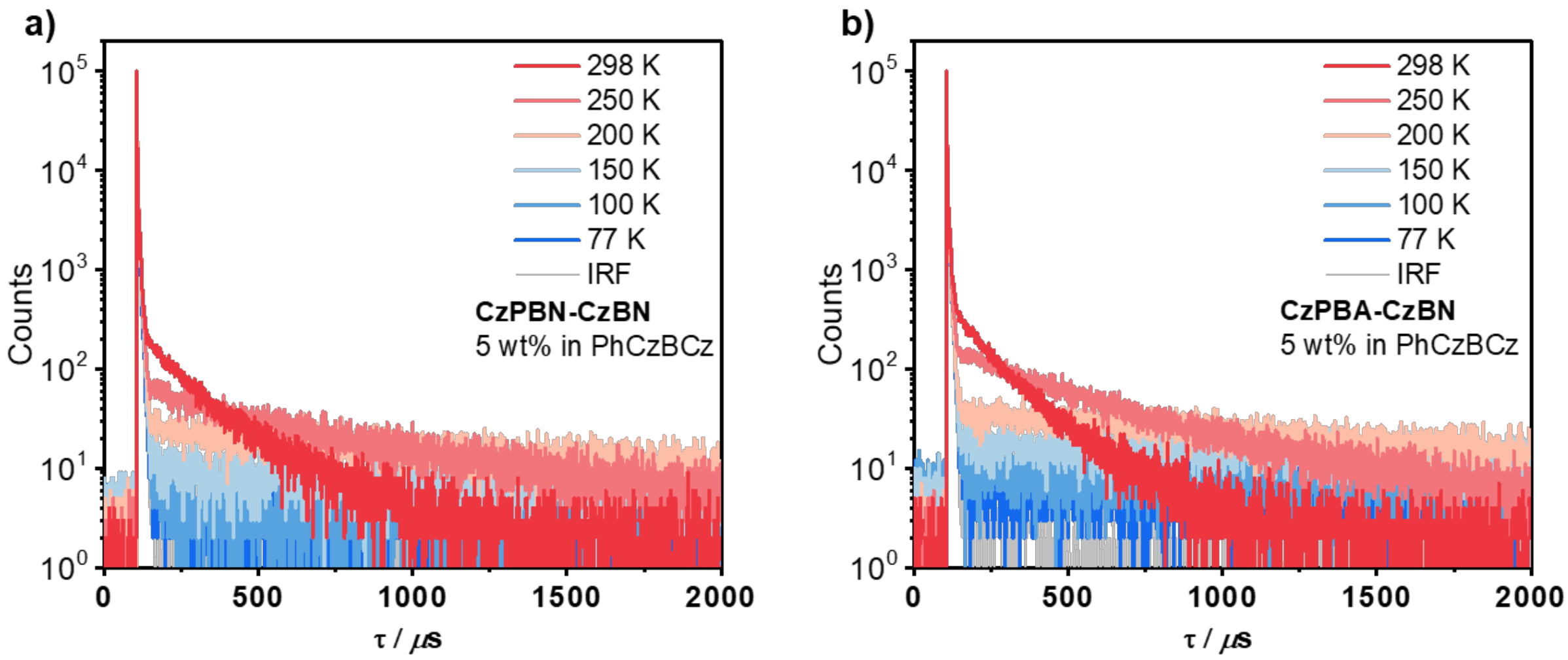

Figure **S10**. Temperature-dependent TRPL decays of the delayed emission for 5 wt% doped films of a) **CzPBN-CzBN** and b) **CzPBA-CzBN** in PhCzBCz (λ_{exc} = 340 nm).

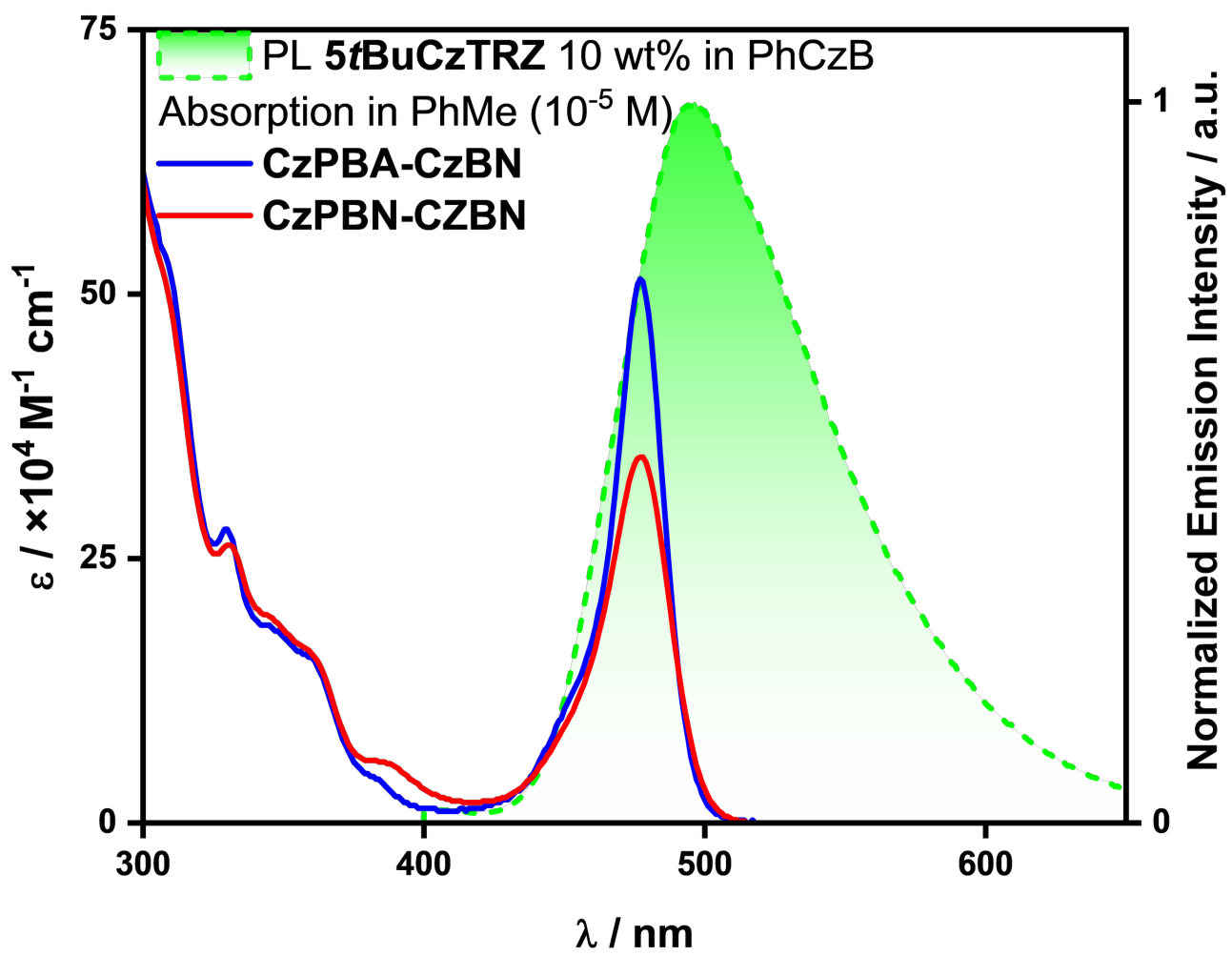

Figure **S11**. Absorption spectra of dilute solutions of **CzPBN-CzBN** and **CzPBA-CzBN** in toluene (10^{-5} M) and SS-PL spectrum of 10 wt% doped film of **5*t*BuCzTRZ** in PhCzBCz (λ_{exc} = 340 nm).

Table **S5**. Photophysical data of a 10 wt% doped film of **5*t*BuCzTRZ** in PhCzBCz.

Emitter	λ_{PL} (FHWM) / nm	Φ_{PL}/ %	τ_p / ns	τ_d / µs	$k_{ISC}{}^{j}$ / ×10^8 s^{-1}	$k_{RISC}{}^{j}$ / ×10^6 s^{-1}	$k_{s\_r}{}^{j}$/ ×10^7 s^{-1}	$k_{s\_nr}{}^{j}$ / s^{-1}

**5*t*BuCzTRZ**	497 (90)	~100 /81	6.1	1.54	1.39	5.52	1.92	--

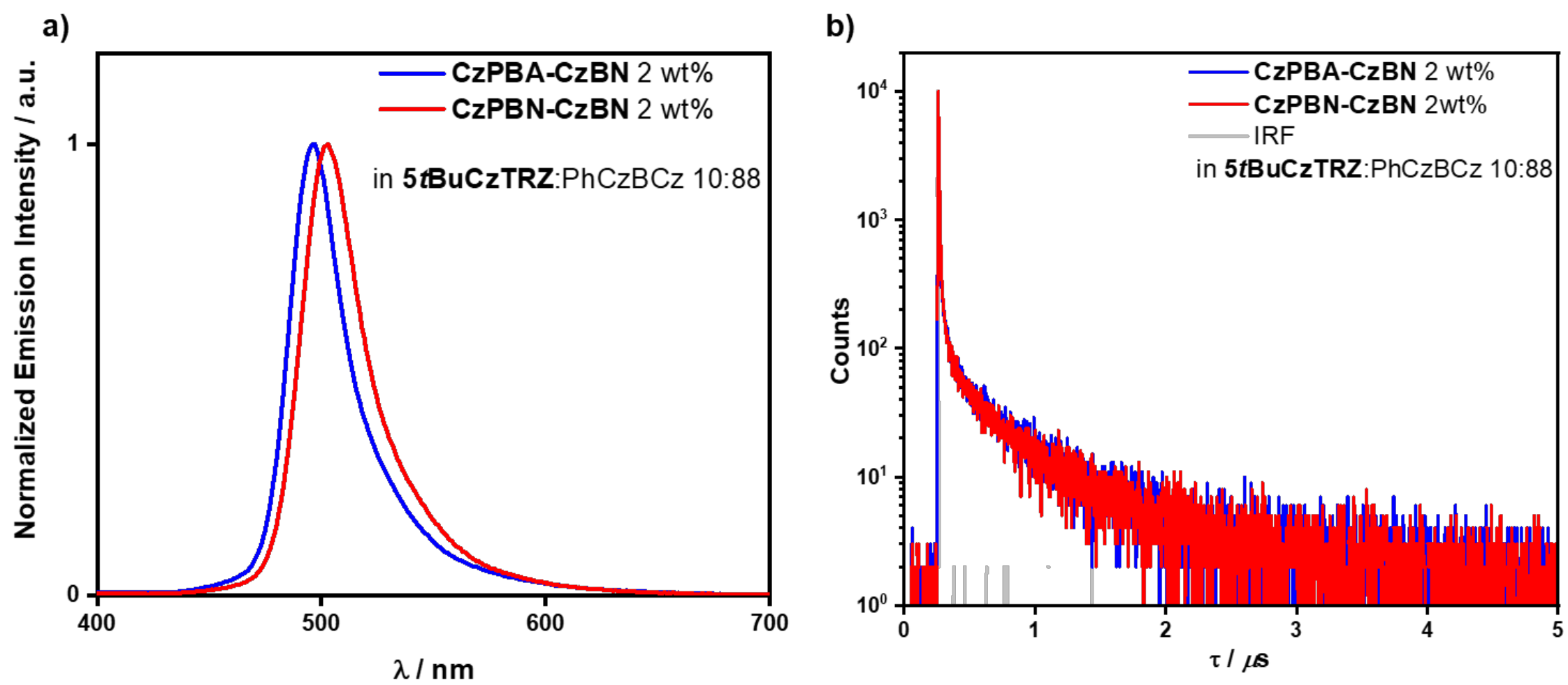

Figure **S12**. a) SS-PL spectra of 2 wt% doped films in **5*t*BuCzTRZ**: PhCzBCz 10:88 of **CzPBN-CzBN** (red) and **CzPBA-CzBN** (blue) (λ_{exc} = 340 nm). b) TRPL decays of 2 wt% doped films in **5*t*BuCzTRZ**:PhCzBCz 10:88 of **CzPBN-CzBN** (red) and **CzPBA-CzBN** (blue) (λ_{exc} = 340 nm).

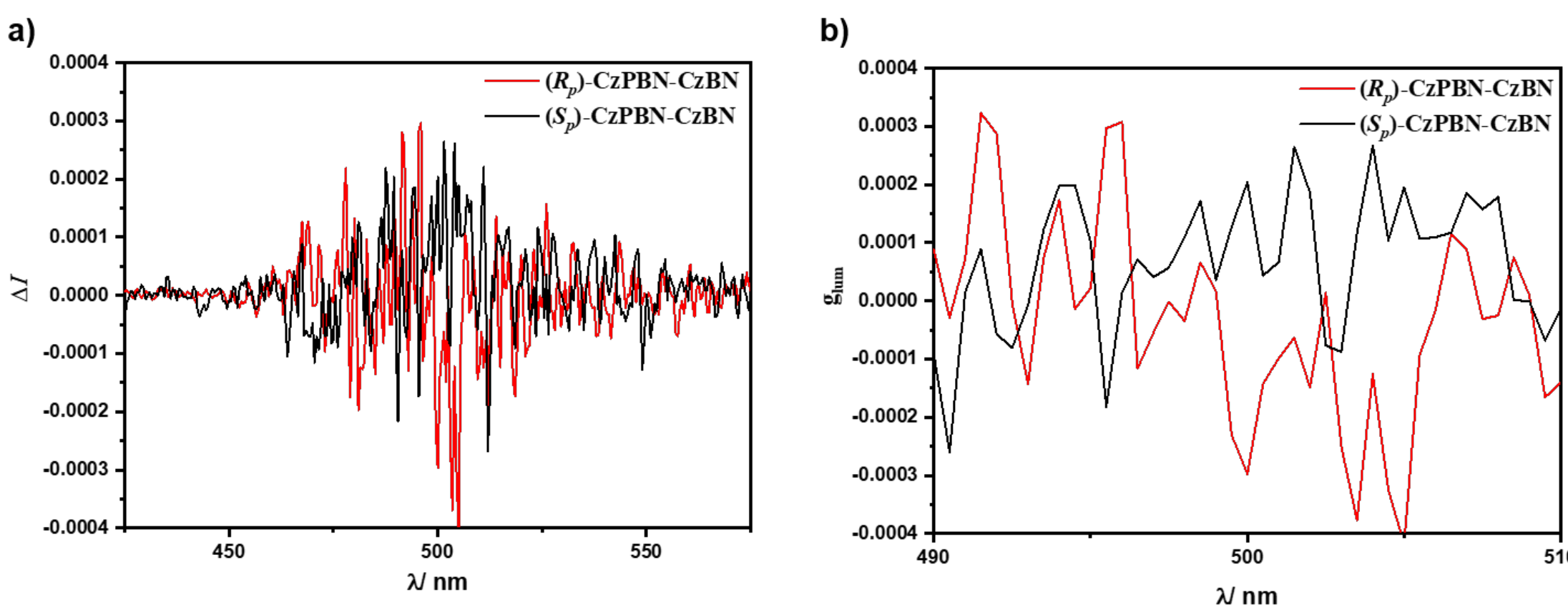

Figure **S13**. CPL and d) g_{lum} spectra of **(*R*p)-CzPBN-CzBN** (red) and **(*S*p)-CzPBN-CzBN** (black) in dilute toluene solution (10^{-6} M; λ_{exc} = 350 nm).

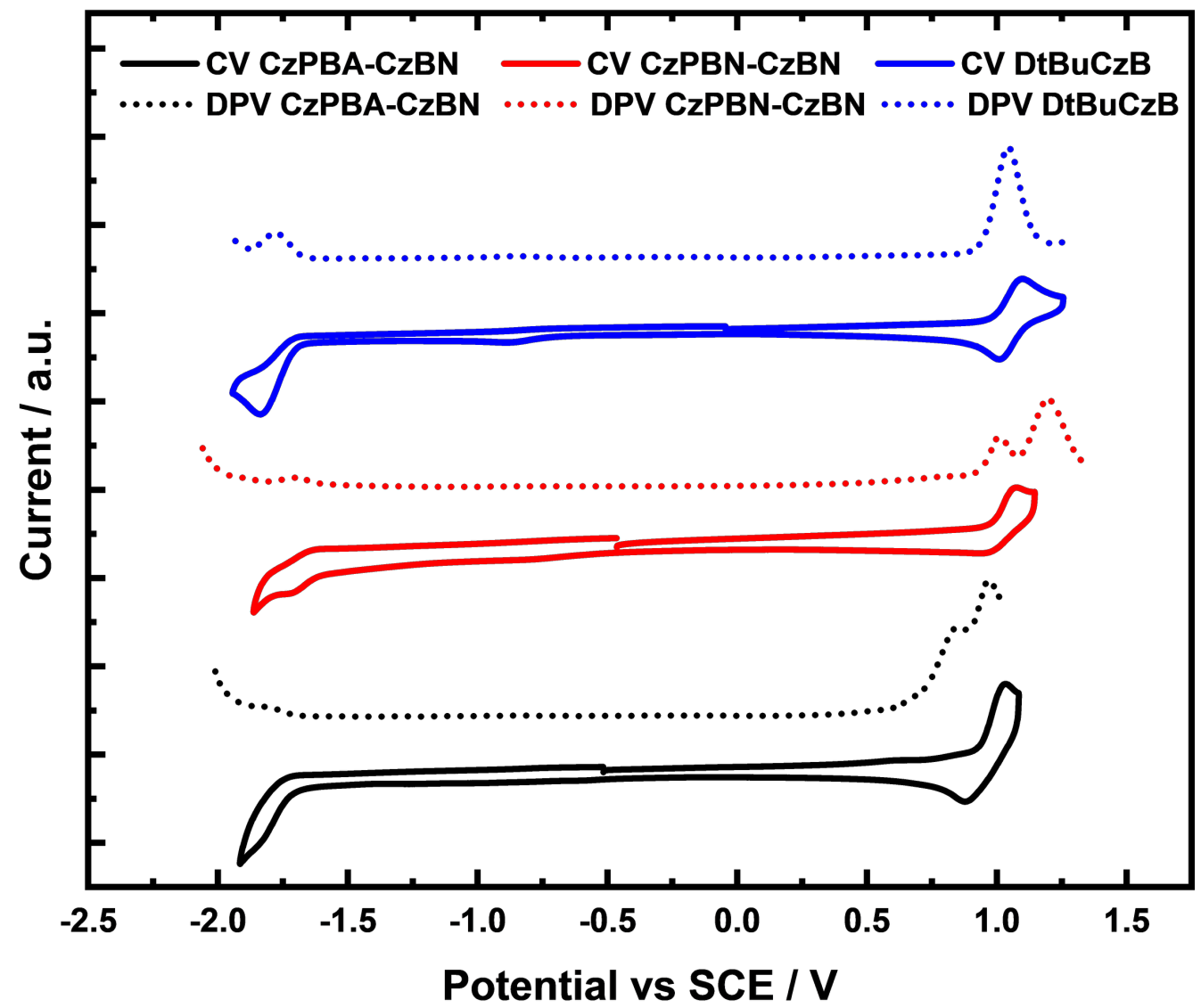

Figure **S14**. CV (solid) and DPV (dashed) of **CzPBA-CzBN** (black), **CzPBN-CzBN** (red), and **DtBuCzB** (blue). CV and DPV performed in DCM solution (0.1 M [^{n}Bu$_4$N]PF$_6$) with a scan rate of 0.1 V/s and referenced to SCE ($E_{ox/red}$ [V *vs.* SCE] = $E_{ox/red}$ [V *vs* Fc/Fc$^+$] + 0.46).

Table **S6**. Oxidation and reduction potentials and calculated HOMO and LUMO energy levels for **CzPBN-CzBN** and **CzPBA-CzBN**.

	$E_{ox}{}^{a}$ / V	$E_{red}{}^{a}$ / V	$E_{HOMO}{}^{b}$ / eV	$E_{LUMO}{}^{c}$ / eV	$\Delta E_{HOMO-LUMO}{}^{d}$ / eV
**CzPBN-CzBN**	1.01	-1.71	-5.35	-2.63	2.72
**CzPBA-CzBN**	0.85	-1.81	-5.19	-2.53	2.66
**DtBuCzB**	1.09	-1.73	-5.39	-2.57	2.82

[a] Oxidation and reduction potential derived from peaks from the DPV performed in DCM solution (0.1 M [^{n}Bu$_4$N]PF$_6$) with a scan rate of 0.1 V/s and referenced to SCE. [b] HOMO energy calculated from -(E_{ox}+4.8). [c] LUMO energy calculated from -(E_{red}+4.8). [d] $\Delta E_{HOMO/LUMO}$ = | E_{HOMO} - E_{LUMO}|.

Separation of Enantiomers

Additional information on the characterization of the target compound is available via the repository Chemotion: https://dx.doi.org/10.14272/DDPABGAGYTYRQK-UHFFFAOYSA-N.3

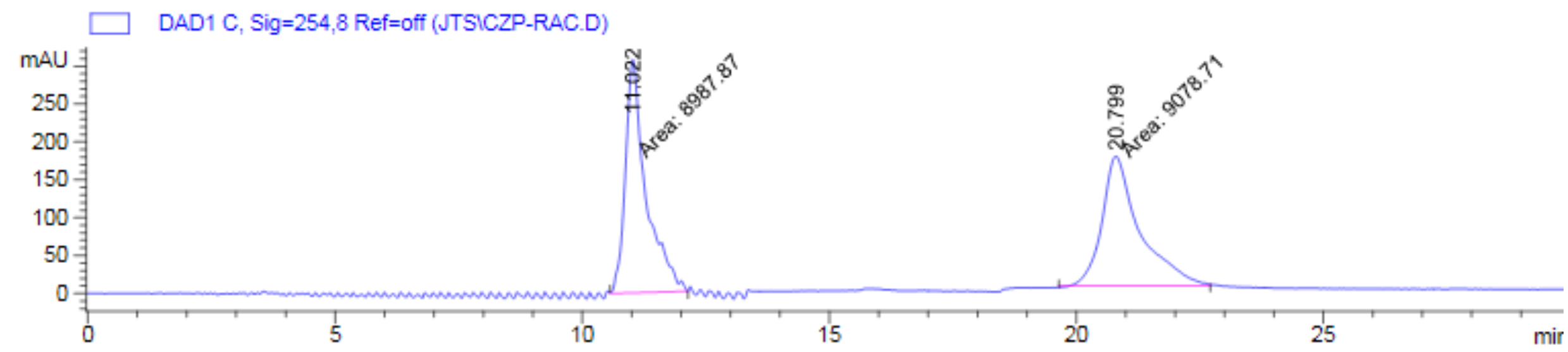

Signal 3: DAD1 C, Sig=254,8 Ref=off

Peak #	RetTime [min]	Type	Width [min]	Area [mAU*s]	Height [mAU]	Area %
1	11.022	MM	0.4861	8987.86621	308.13406	49.7486
2	20.799	MM	0.8864	9078.70605	170.70435	50.2514
Totals :				1.80666e4	478.83841	

Figure **S15**. Chiral HPLC trace of racemic **CzP**.

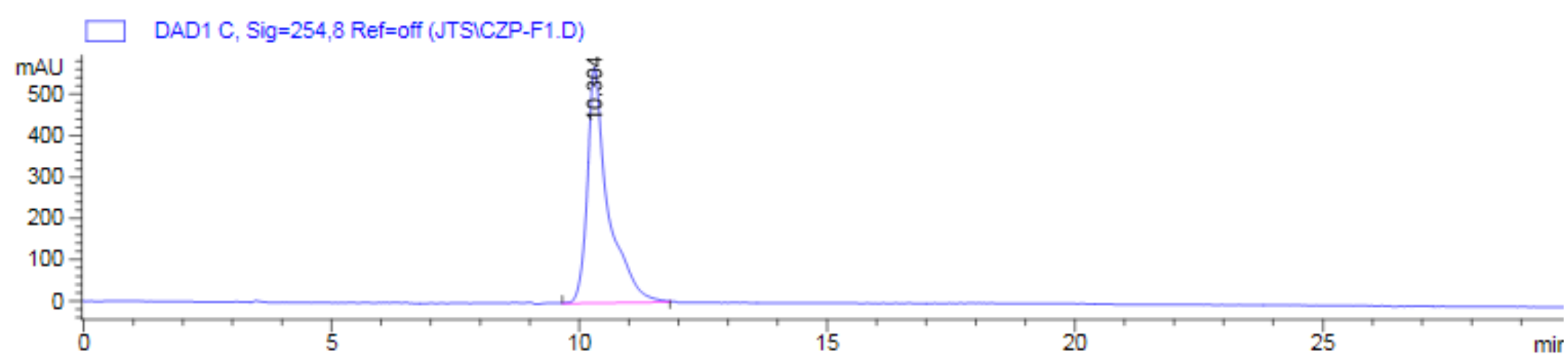

Signal 3: DAD1 C, Sig=254,8 Ref=off

Peak #	RetTime [min]	Type	Width [min]	Area [mAU*s]	Height [mAU]	Area %
1	10.304	VB	0.4063	1.66669e4	571.48022	100.0000
Totals :				1.66669e4	571.48022	

Figure **S16**. Chiral HPLC trace of enantiopure **(*S*$_p$)-CzP**.

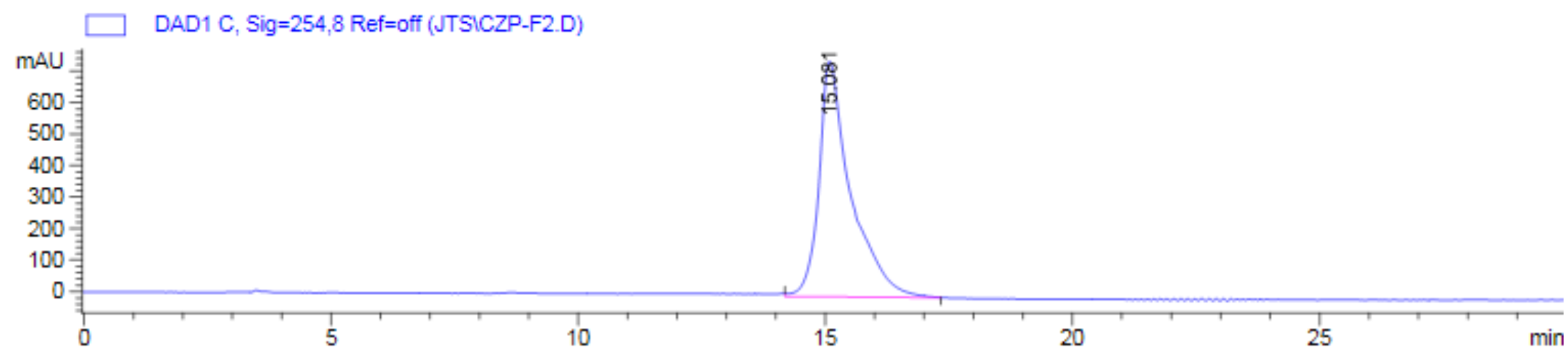

Signal 3: DAD1 C, Sig=254,8 Ref=off

Peak #	RetTime [min]	Type	Width [min]	Area [mAU*s]	Height [mAU]	Area %
1	15.081	VB	0.6098	3.35816e4	748.02728	100.0000
Totals :				3.35816e4	748.02728	

Figure **S17**. Chiral HPLC trace of enantiopure ***(R*~p~)-CzP**.

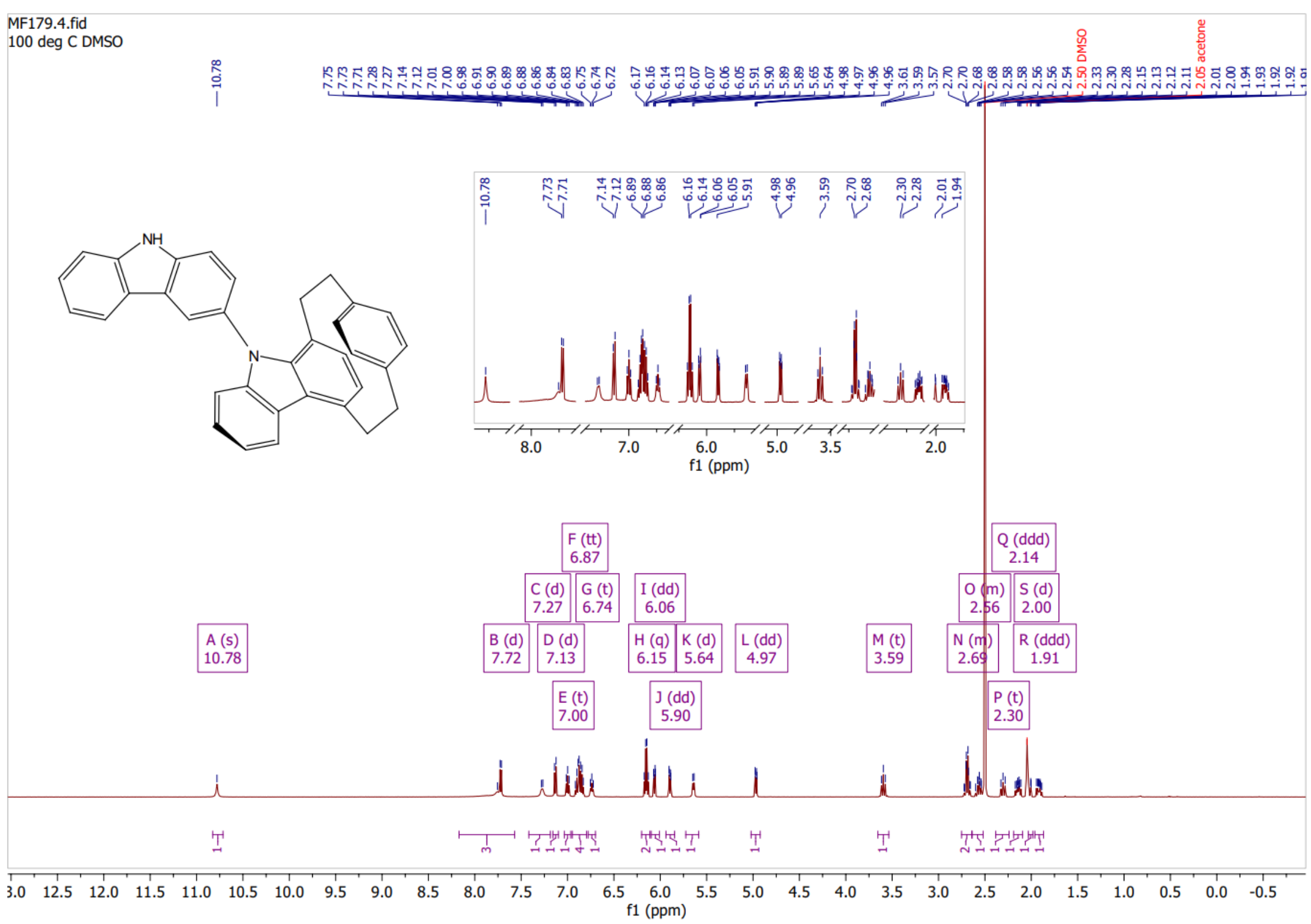

Figure **S18**. ^{1}H-NMR spectrum of **CzP-Cz** in DMSO-d_6 at 100 °C.

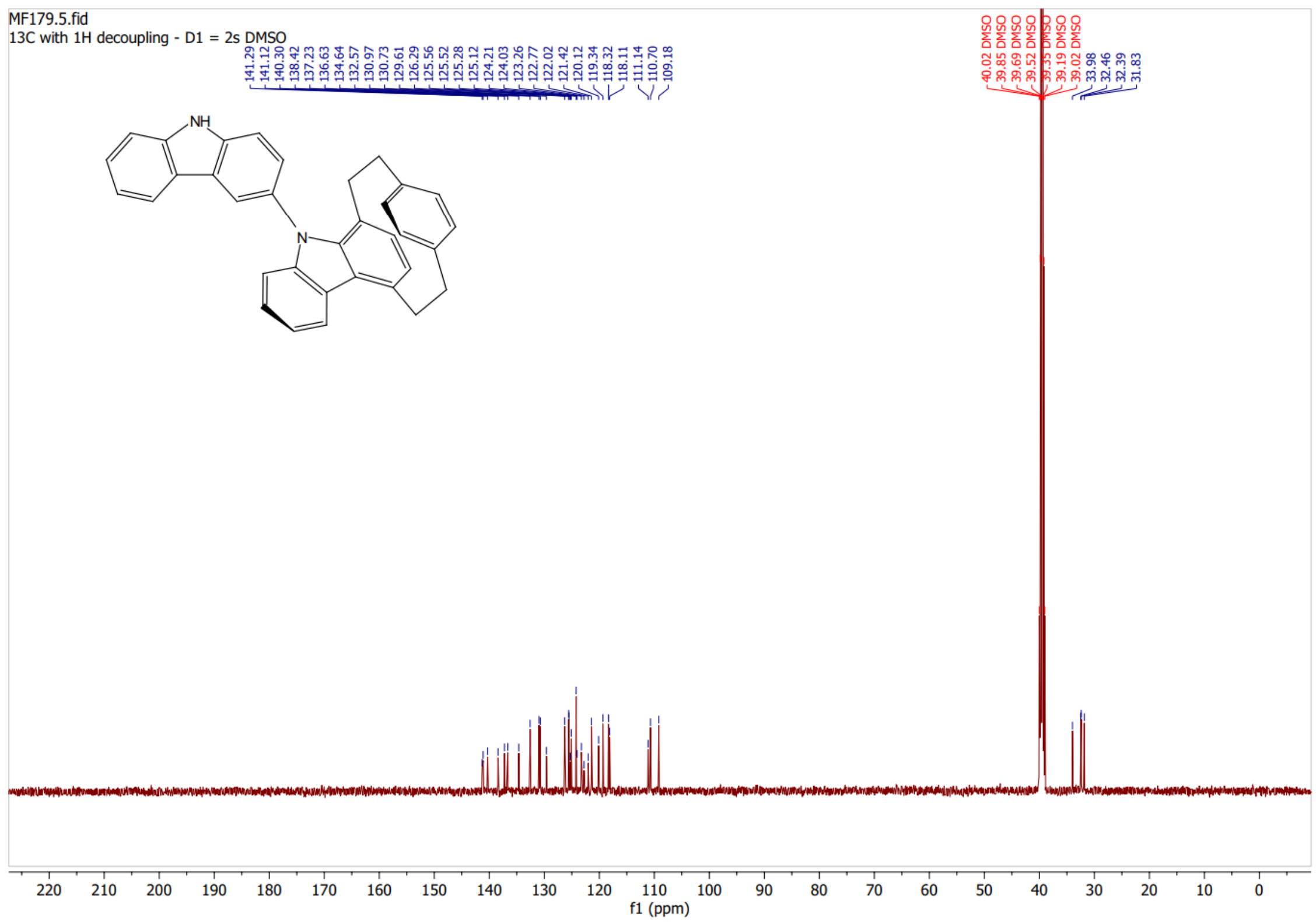

Figure **S19**. ^{13}C-NMR spectrum of **CzP-Cz** in DMSO-d_6 at 100 °C.

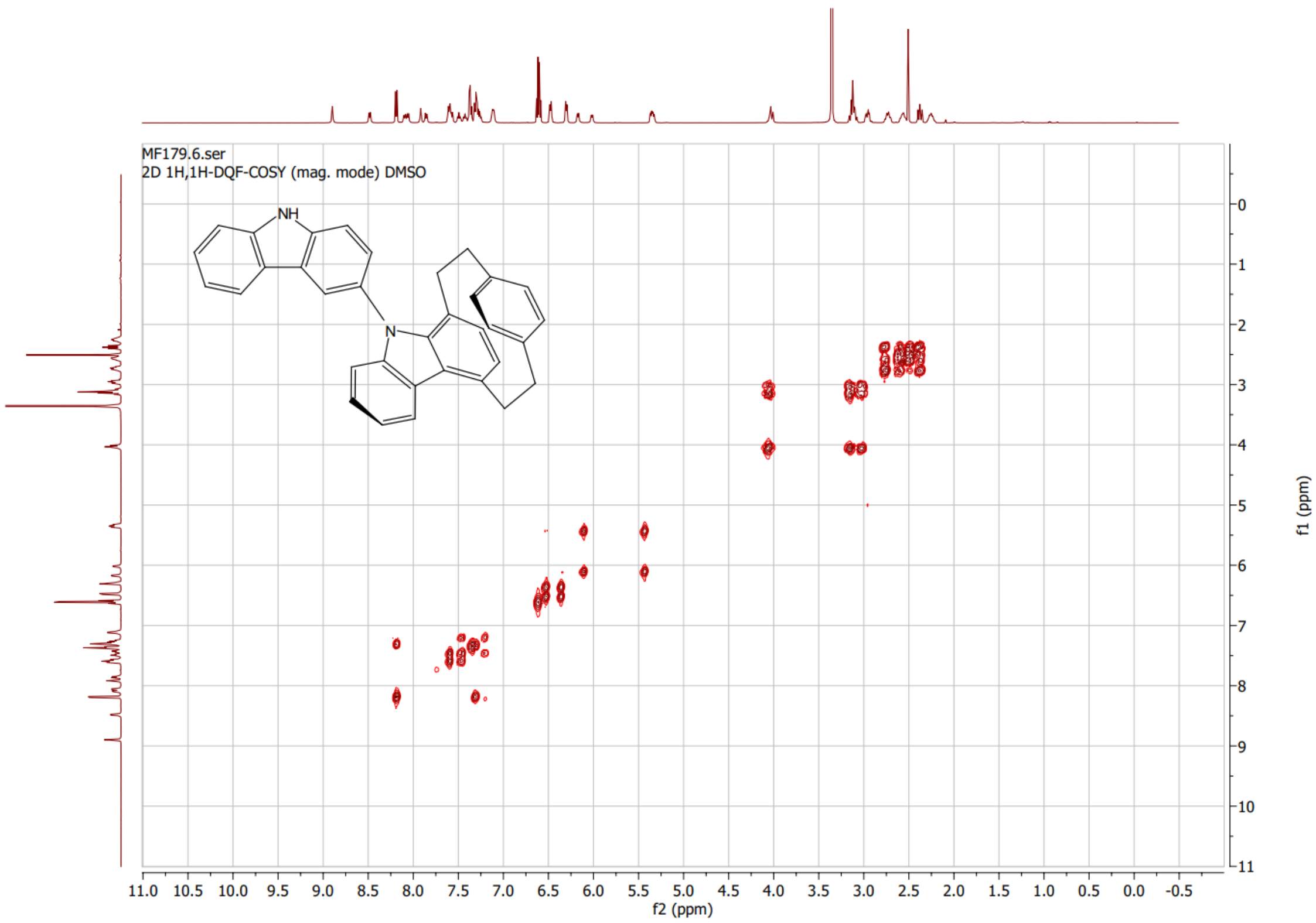

Figure **S20**. ^{1}H-^{1}H COSY NMR spectrum of **CzP-Cz** in DMSO-d_6 at 100 °C.

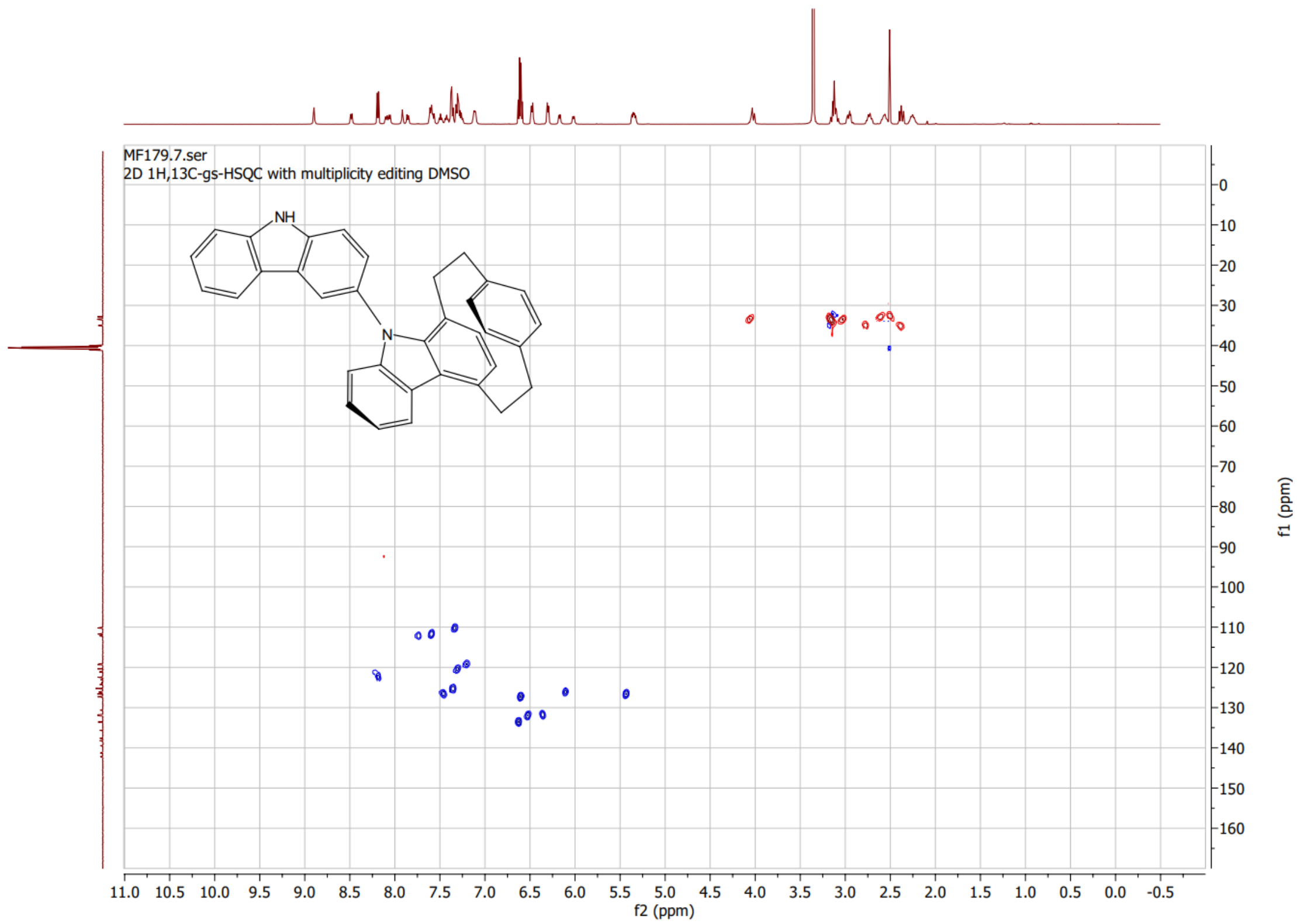

Figure **S21**. ^{1}H-^{13}C-HSQC NMR spectrum of **CzP-Cz** in DMSO-d_6 at 100 °C.

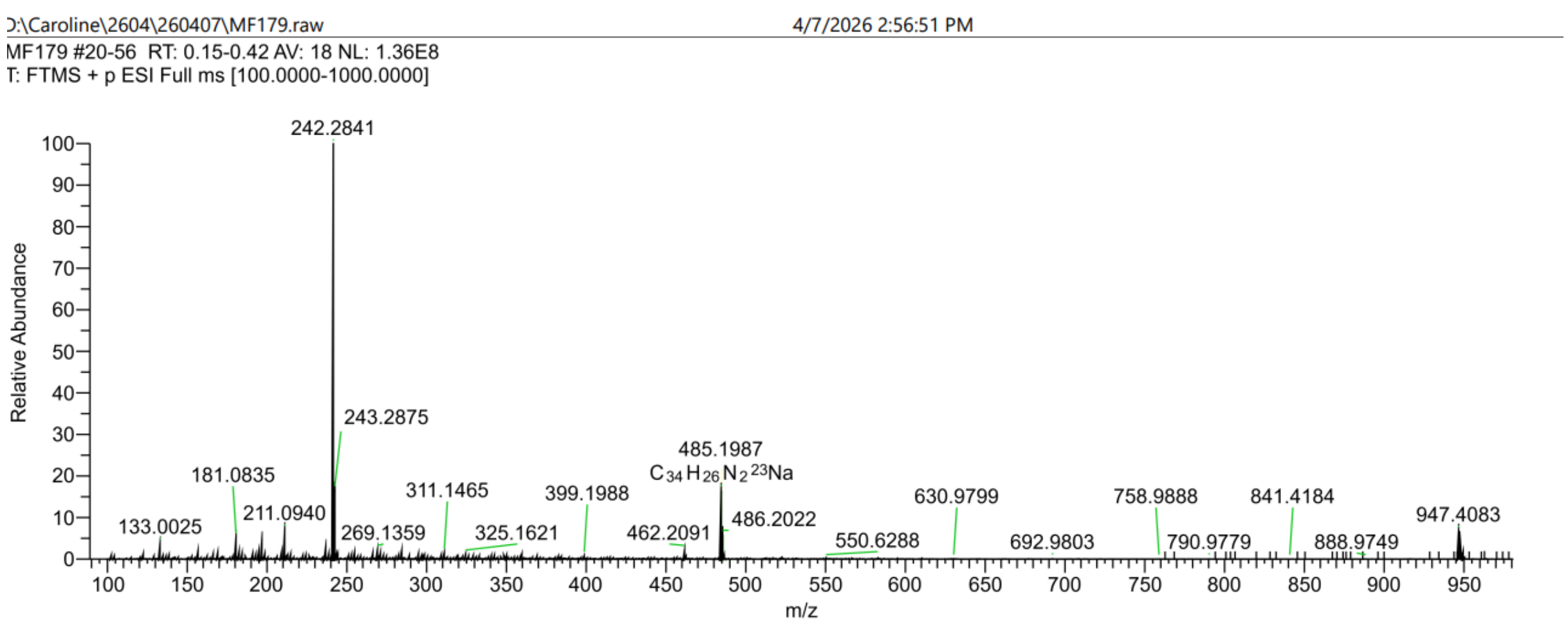

Peak Mass	Display Formula	S Fit	RDB	Delta [ppm]	Theo. mass	Rank	MSMS Matched Frag.
485.1987	$C_{34}H_{26}N_2{}^{23}Na$	90.8887087623133	22.50	-0.23	485.19882	1	(Collection)
485.1987	$C_{32}H_{27}N_2{}^{23}Na_2$	7.17645049193995	19.50	4.72	485.19641	2	(Collection)

Figure **S22**. HRMS (ESI-MS) spectrum of **1[9]-(9*H*-carbazol-3-yl)-1[9]*H*-1(1,4)-carbazola-4(1,4)-benzenacyclohexaphane** (**CzP-Cz**)

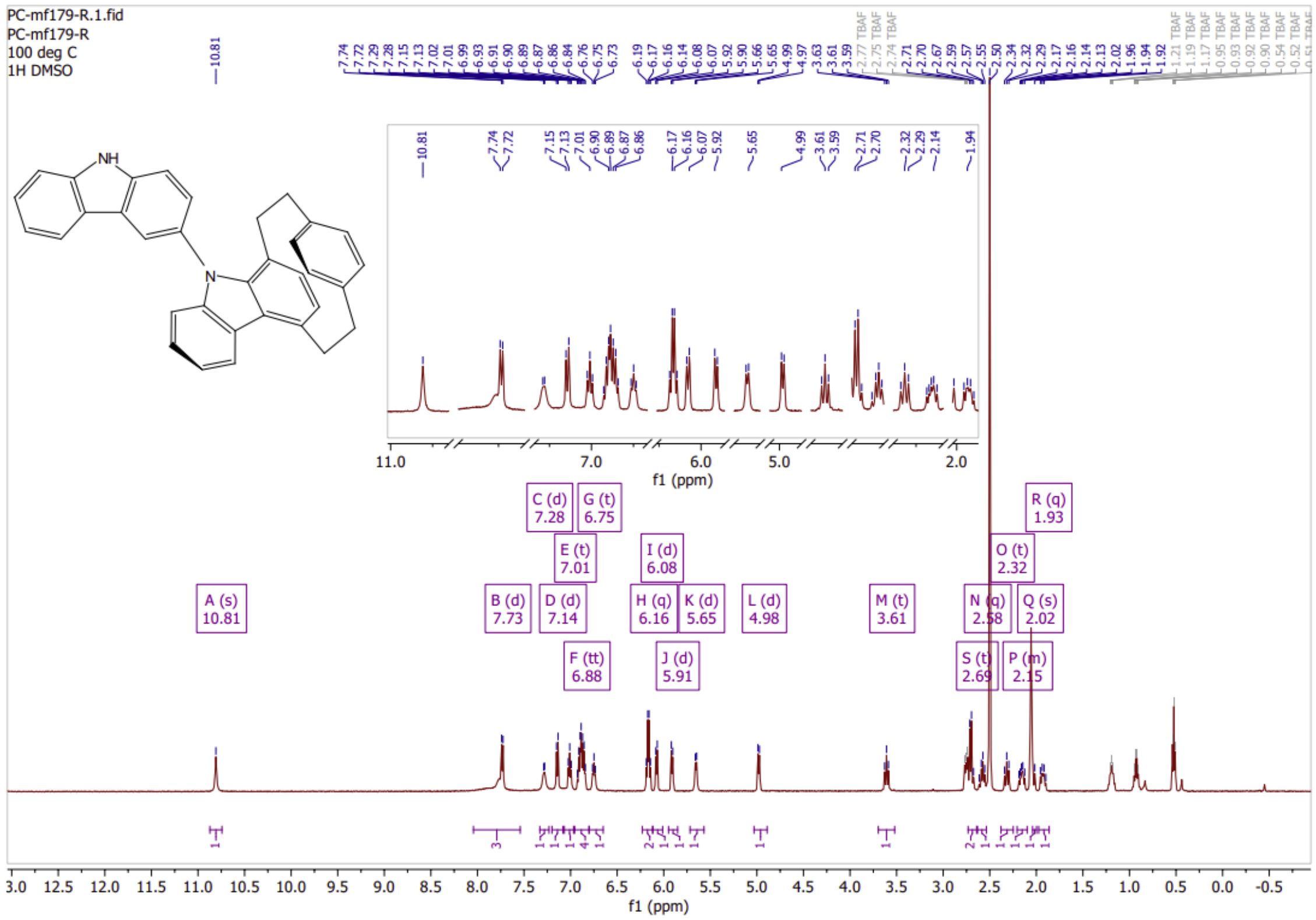

Figure **S23**. ^{1}H-NMR spectrum of ***(R*$_p$)-CzP-Cz** in DMSO-d_6 at 100 °C.

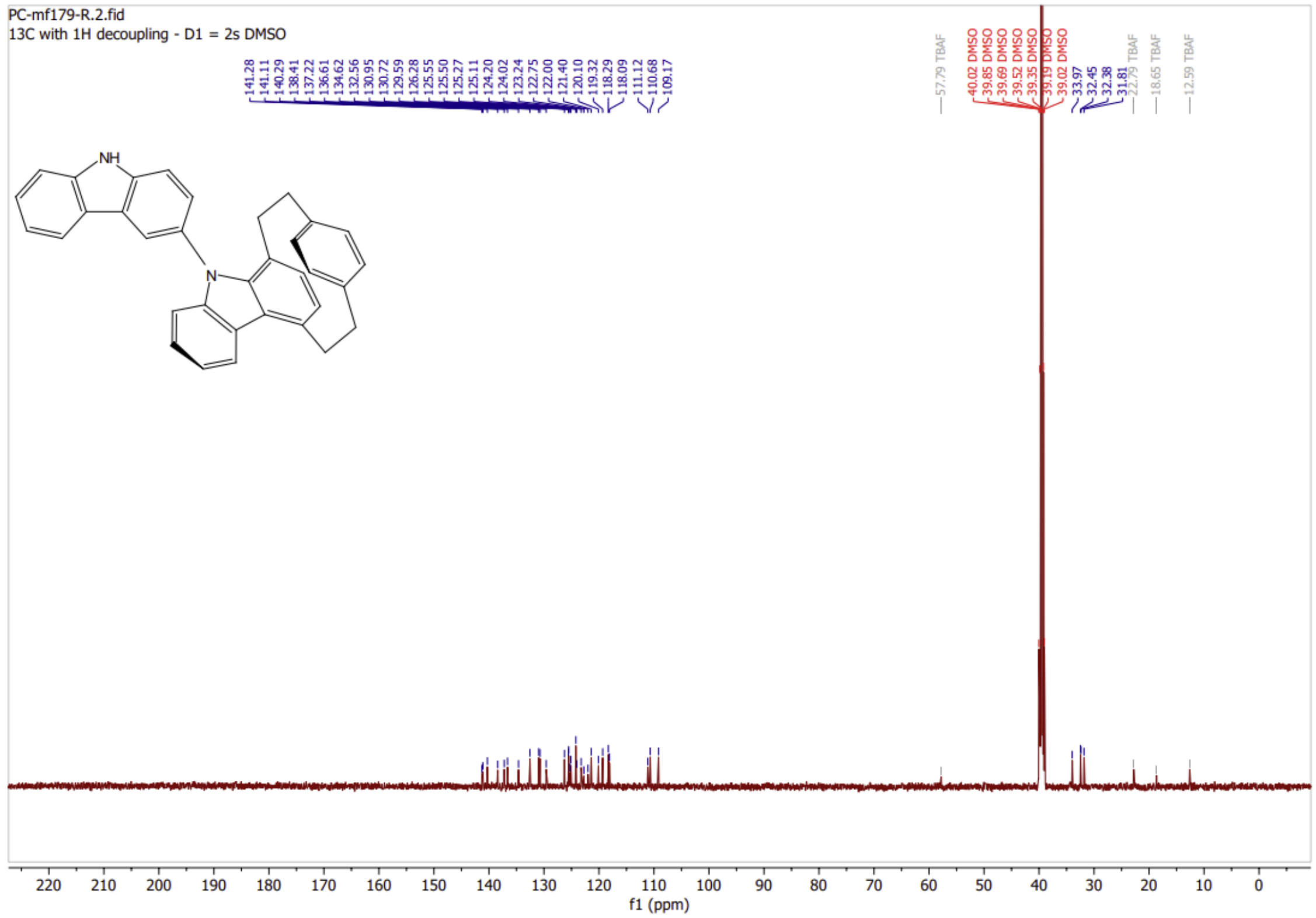

Figure **S24**. ^{13}C-NMR spectrum of ***(R*$_p$*)*-CzP-Cz** in DMSO-d_6 at 100 °C.

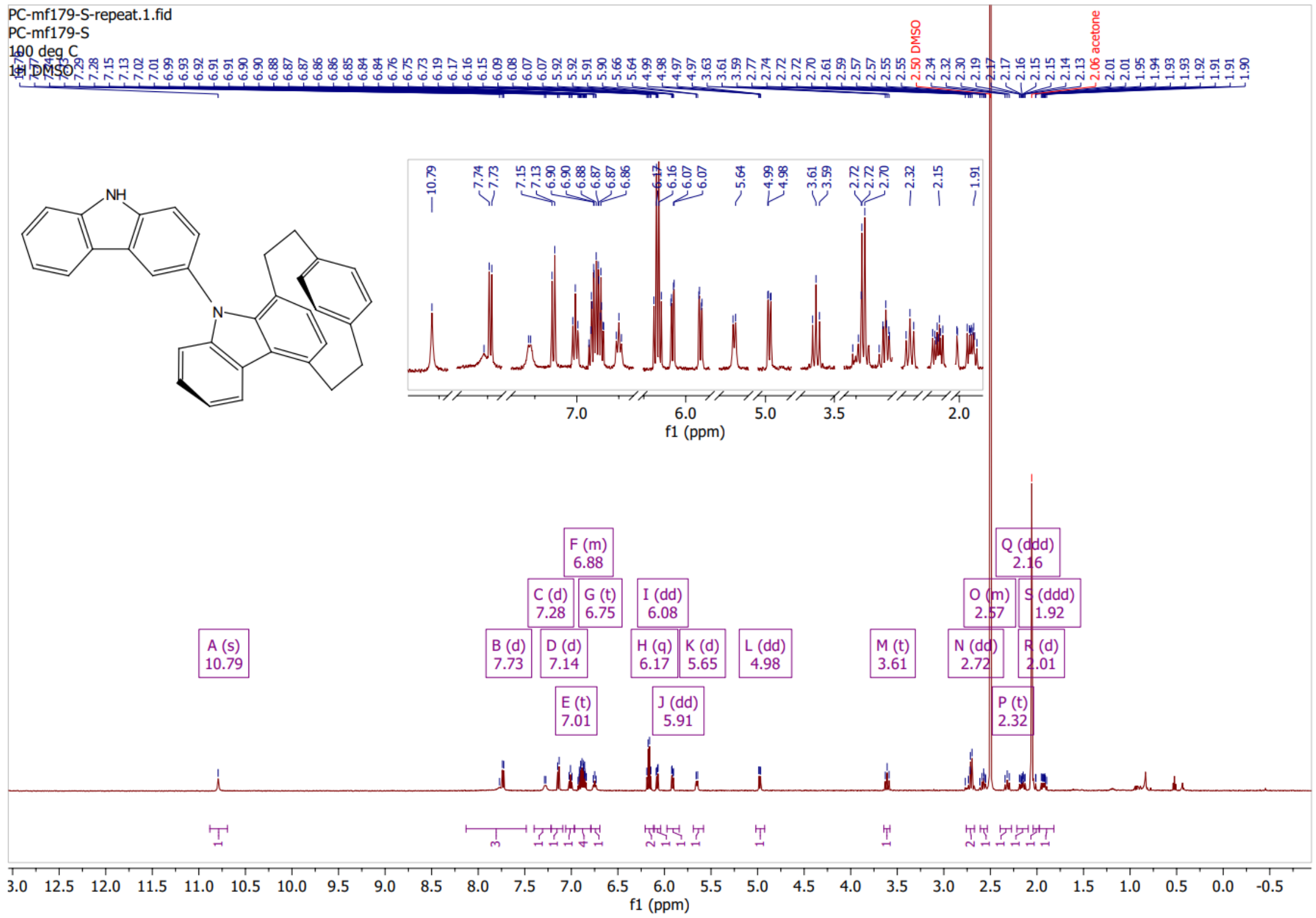

Figure **S25**. ^{1}H-NMR spectrum of **(*S*p)-CzP-Cz** in DMSO-d_6 at 100 °C.

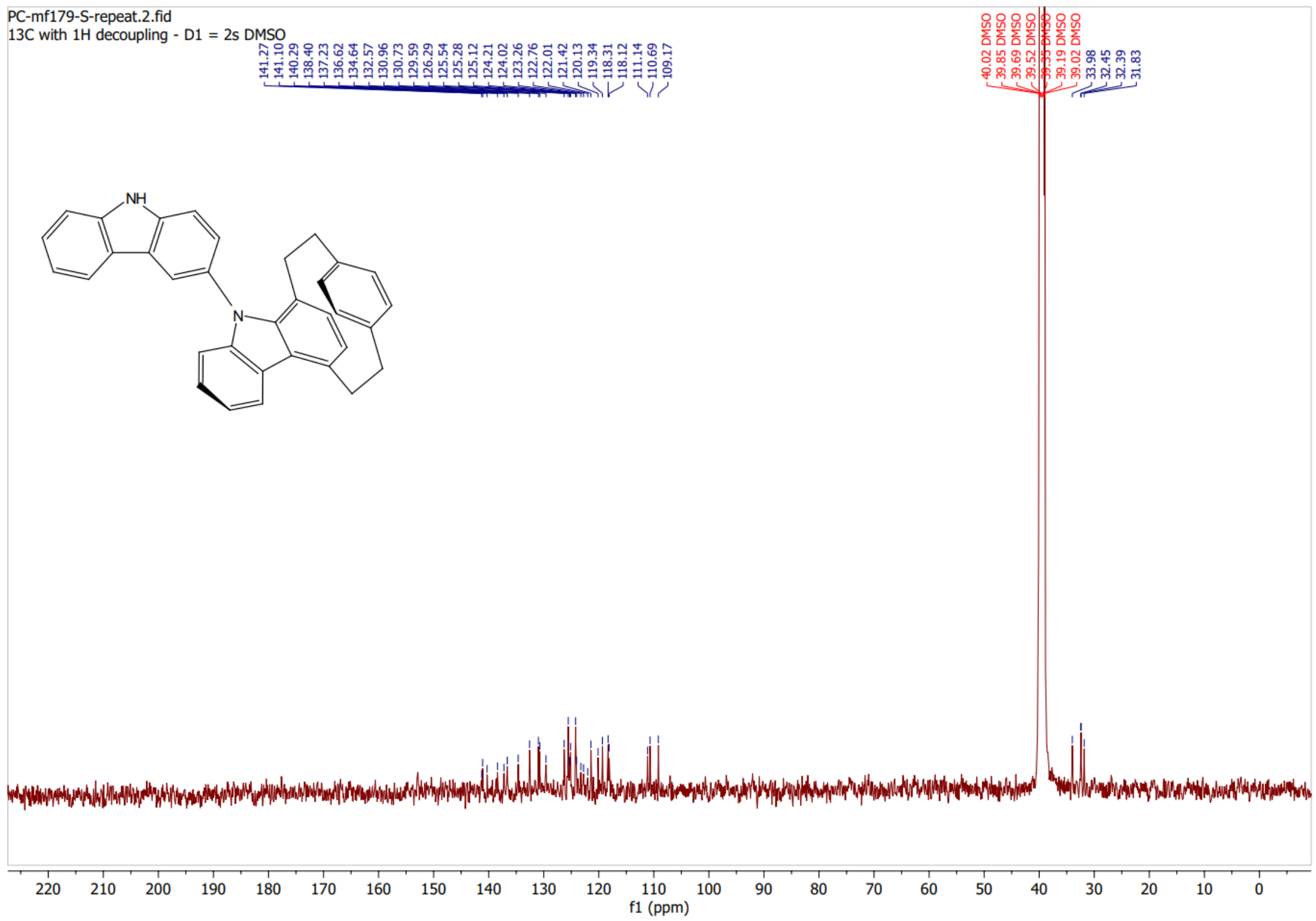

Figure **S26**. ^{13}C-NMR spectrum of ***(S*$_p$)-CzP-Cz** in DMSO-d_6 at 100 °C.

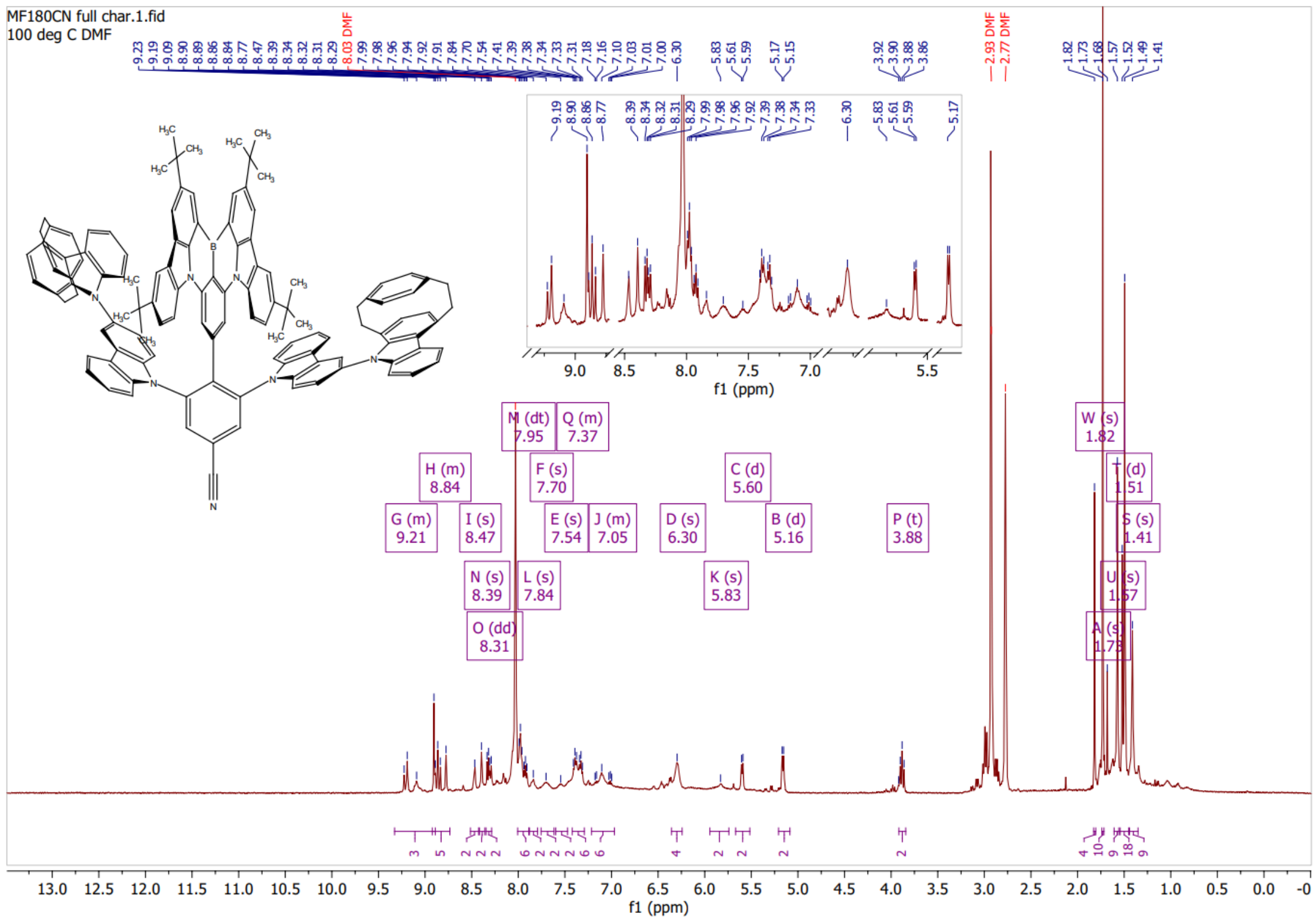

Figure **S27**. ^{1}H-NMR spectrum of **CzPBN-CzBN** in DMF-d_7 at 100 °C.

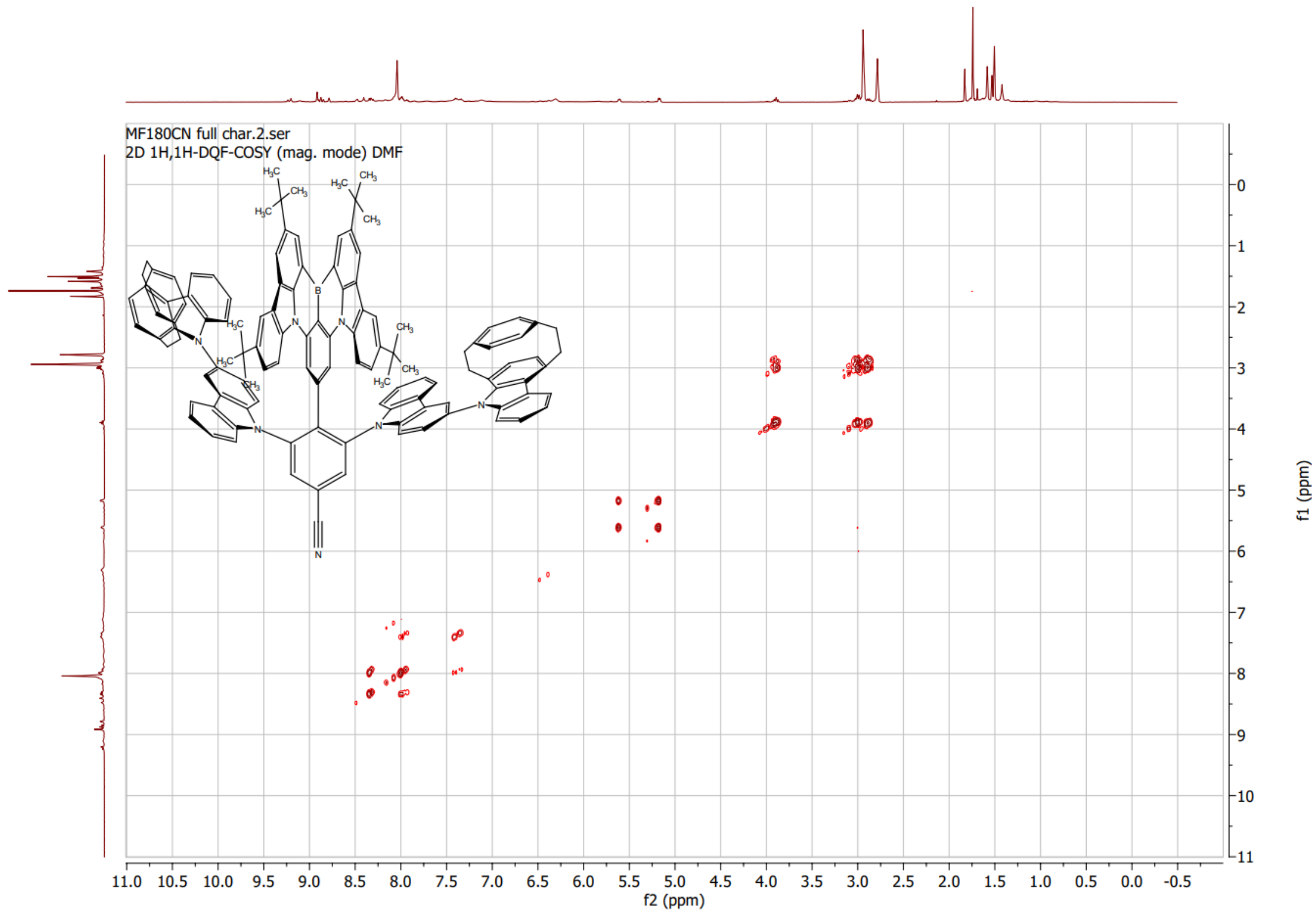

Figure **S28**. ^{1}H-^{1}H COSY NMR spectrum of **CzPBN-CzBN** in DMF-d_7 at 100 °C.

a)

b)

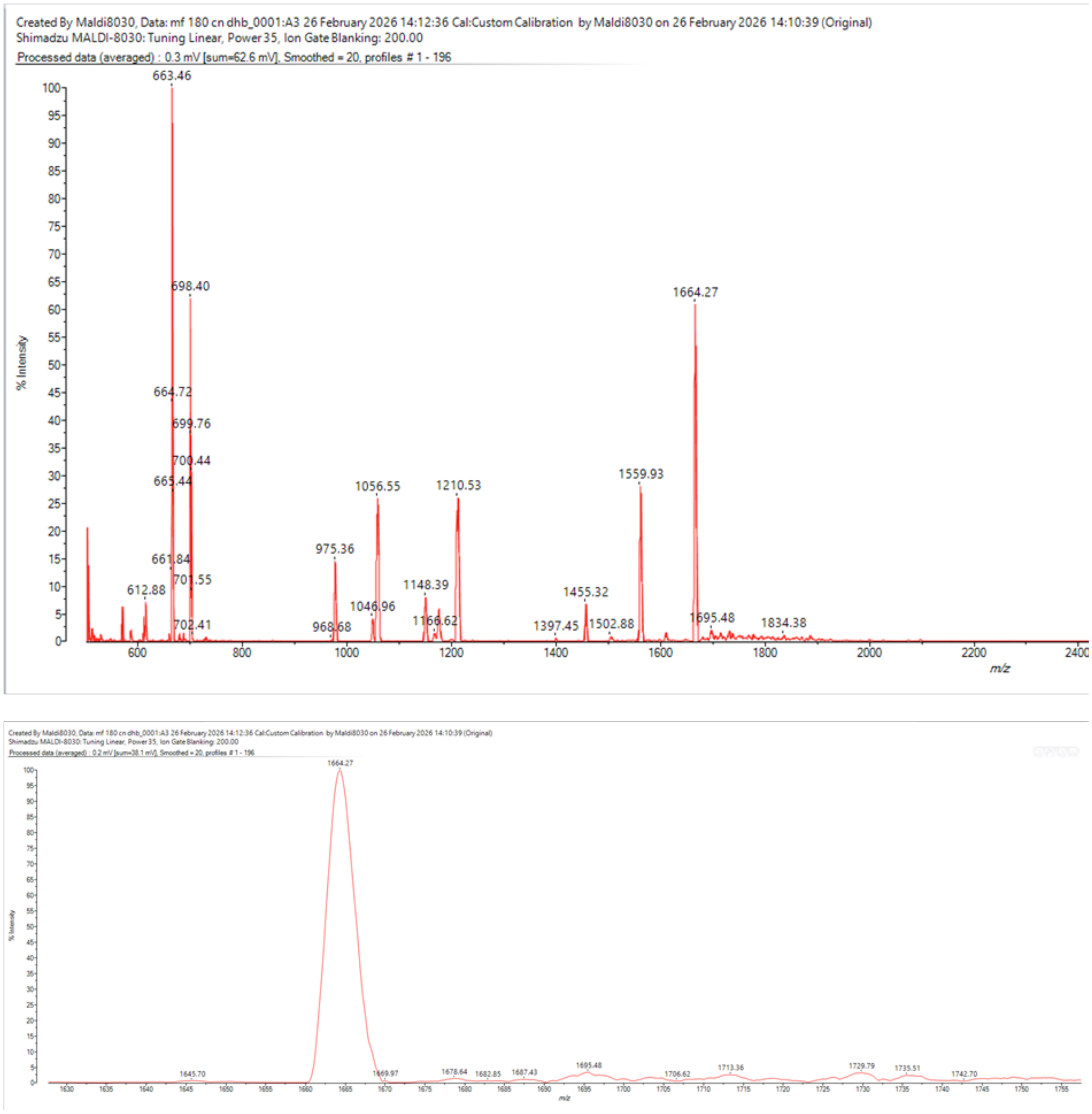

Figure **S29**. HRMS (MALDI-MS) spectrum of **CzPBN-CzBN** b) Zoomed spectrum of corresponding compound peak.

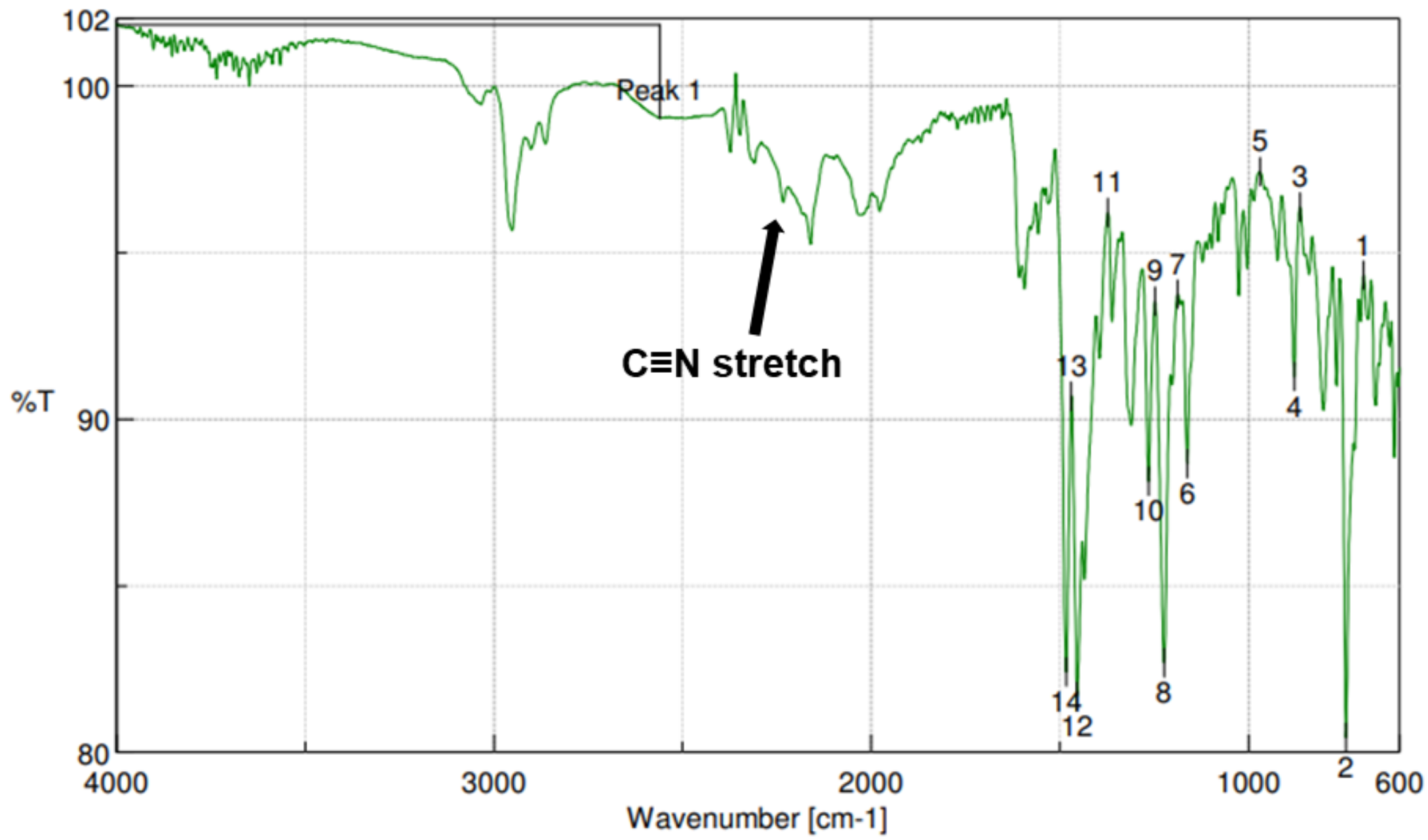

Results of Peak Height

	Peak	Base 1	Base 2	Height
Peak 1	2560	4000	-----	-2.80605

Results of Peak Find

No.	Position	Intensity	No.	Position	Intensity
1	695.212	94.2989	2	741.496	80.4515
3	863.953	96.351	4	878.417	91.2707
5	970.019	97.3945	6	1162.87	88.6555
7	1187.94	93.7376	8	1223.61	82.6752
9	1247.72	93.5288	10	1265.07	88.1287
11	1372.1	96.1767	12	1455.03	81.6697
13	1469.49	90.6822	14	1482.99	82.4048

[Comments]

Sample name	
Comment	
User	
Division	
Company	MCS Limited

[Measurement Information]

Model Name	FT/IR-6100typeA
Serial Number	A055661020
Measurement Date	05/02/2026 11:52
Light Source	Standard
Detector	TGS
Accumulation	64
Resolution	4 cm-1
Zero Filling	On
Apodization	Cosine
Gain	Auto (4)
Aperture	Auto (7.1 mm)
Scanning Speed	Auto (2 mm/sec)
Filter	Auto (10000 Hz)

Figure **S30**. FT-IR spectrum of **CzPBN-CzBN**.

03/12/2025 10:30:28 Page 1 / 1

HPLC Trace Report03Dec2025

<Sample Information>

Sample Name	: mf180.CN_1		
Sample ID	:		
Method Filename	: 100% THF 20 mins 280nm - new-please use.lcm		
Batch Filename	: 02122025.lcb		
Vial #	: 1-3	Sample Type	: Unknown
Injection Volume	: 10 uL		
Date Acquired	: 02/12/2025 17:04:02	Acquired by	: System Administrator
Date Processed	: 02/12/2025 17:24:05	Processed by	: System Administrator

<Chromatogram>

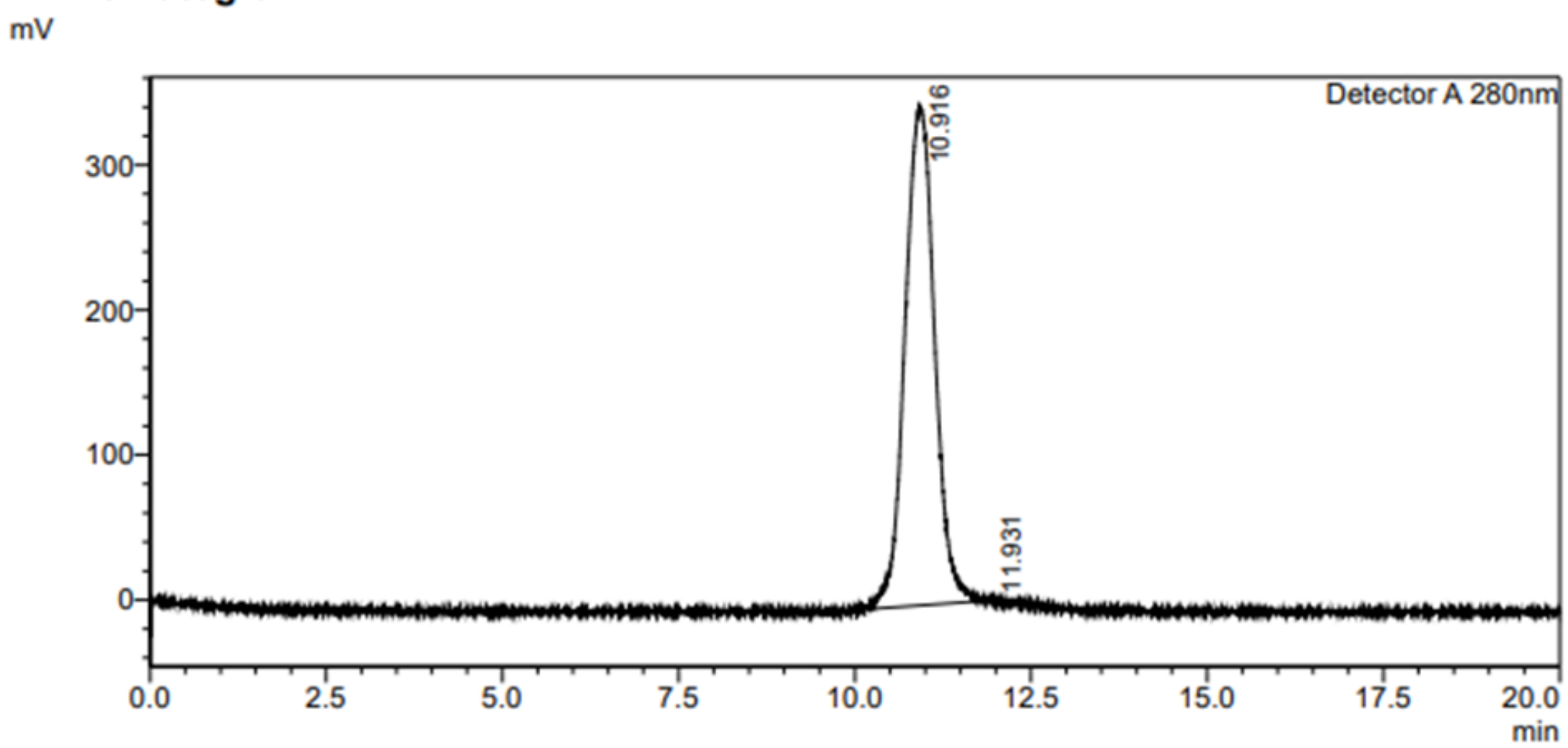

<Peak Table>

Detector A 280nm

Peak#	Ret. Time	Area	Height	Area%	Area/Height	Width at 5% Height
1	10.916	10109842	343315	99.902	29.448	0.994
2	11.931	9947	406	0.098	24.521	--
Total		10119789	343721	100.000		

Figure **S31**. HPLC trace report for **CzPBN-CzBN**.

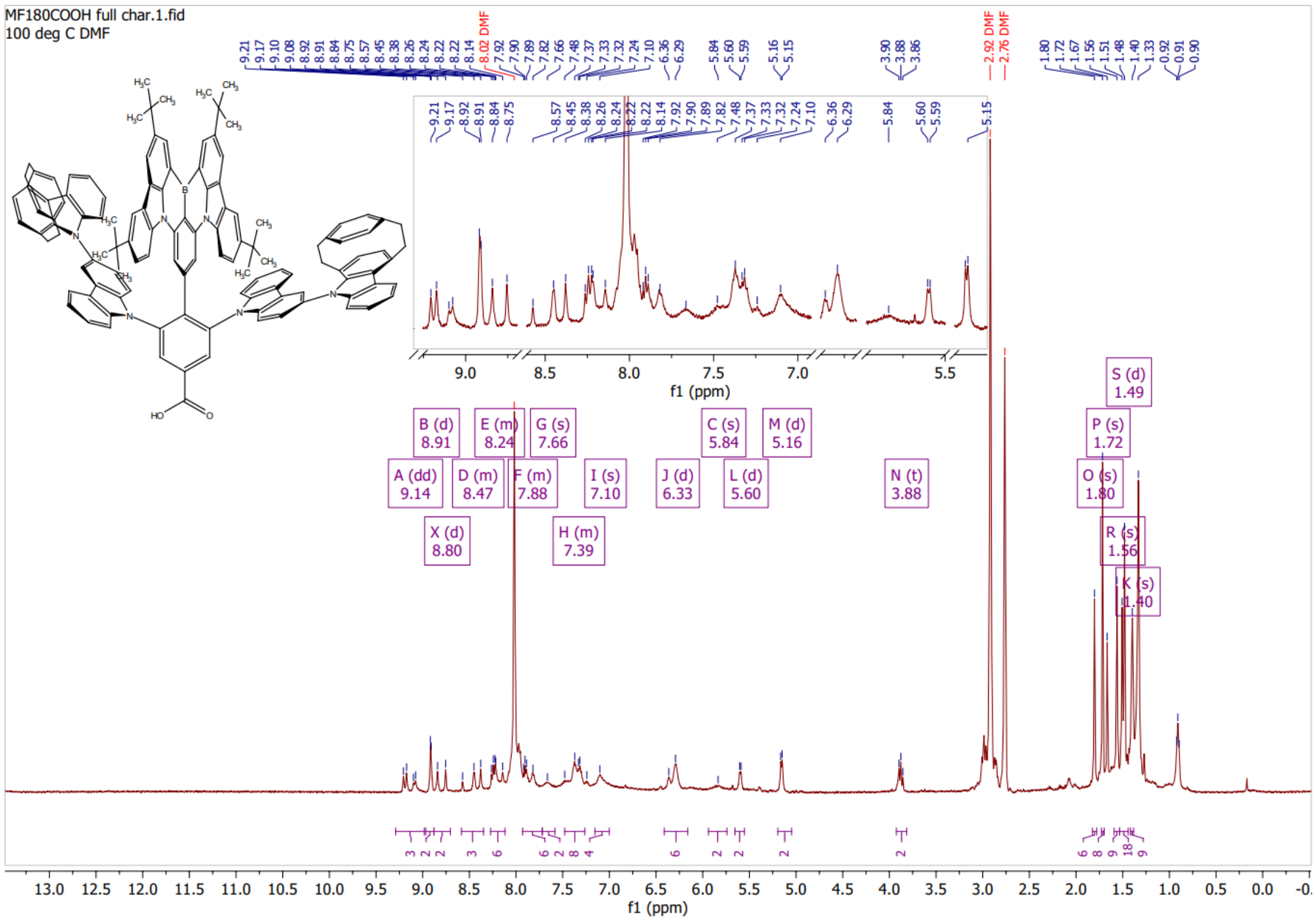

Figure **S32**. ^{1}H-NMR spectrum of **CzPBA-CzBN** in DMF-d_7 at 100 °C.

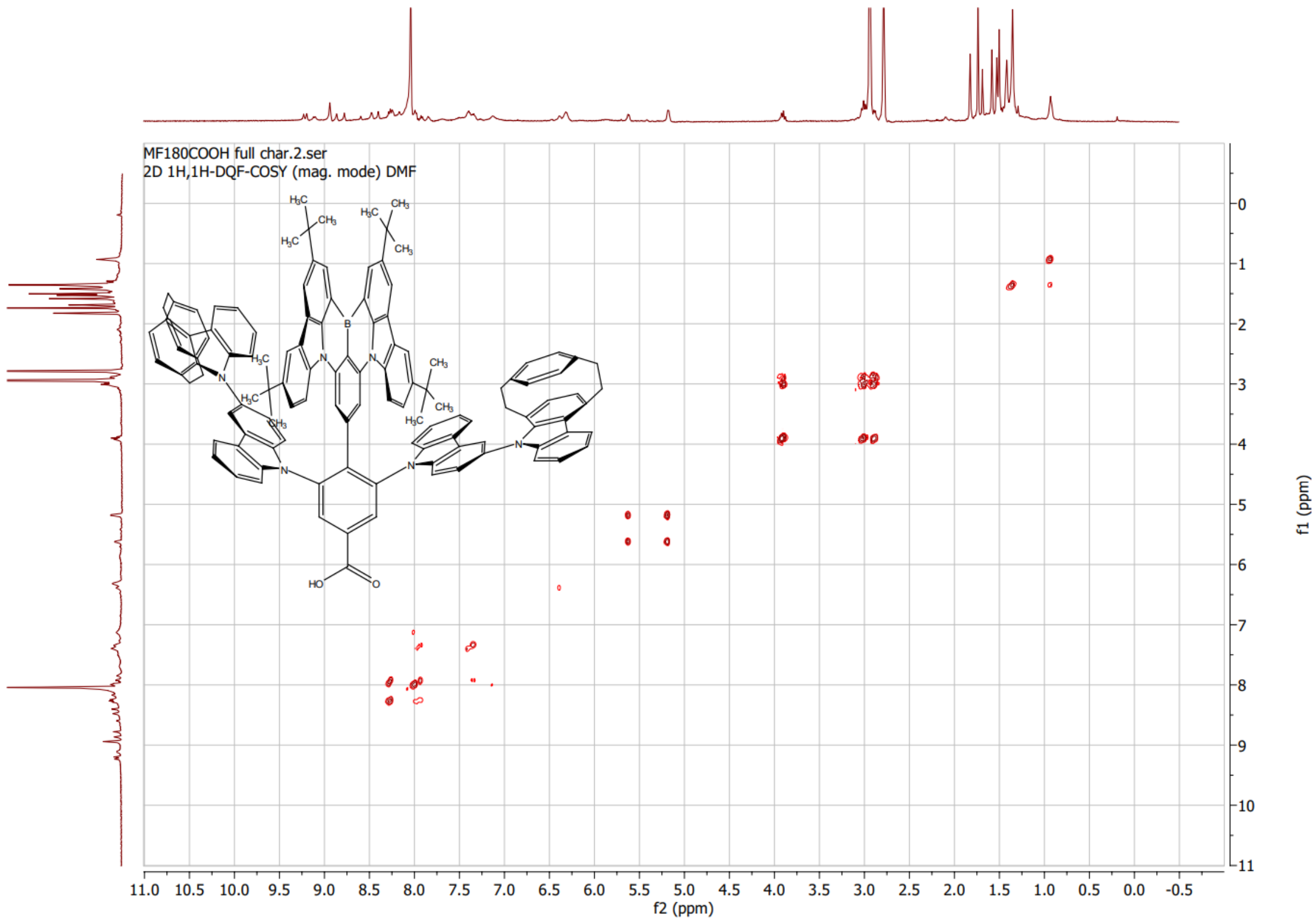

Figure **S33**. ^{1}H-^{1}H COSY NMR spectrum of **CzPBA-CzBN** in DMF-d_7 at 100 °C.

University of Glasgow

Analysis Report

Agilent Trusted Answers

Sample Information

**Name**	85642_MF180 COOH.d
**Data File**	D:\MassHunter\Data\MS Service\2026\January\St Andrews\85642_MF180 COOH-VE.d
**Instrument**	QTOF
**Method (Acq)**	Standard ESI -VE.m
**Inj. Vol. (ul)**	1
**Position**	P1-C5

Sample Chromatograms

Compound Details

Cpd. 1: C121 H101 B N6 O2

Name	Formula	Mass (Tgt)	Mass	Diff (Tgt, ppm)	Score (Tgt)
	C121 H101 B N6 O2	1679.811539	1679.817486	3.54	64.66

Compound Spectra (overlaid)

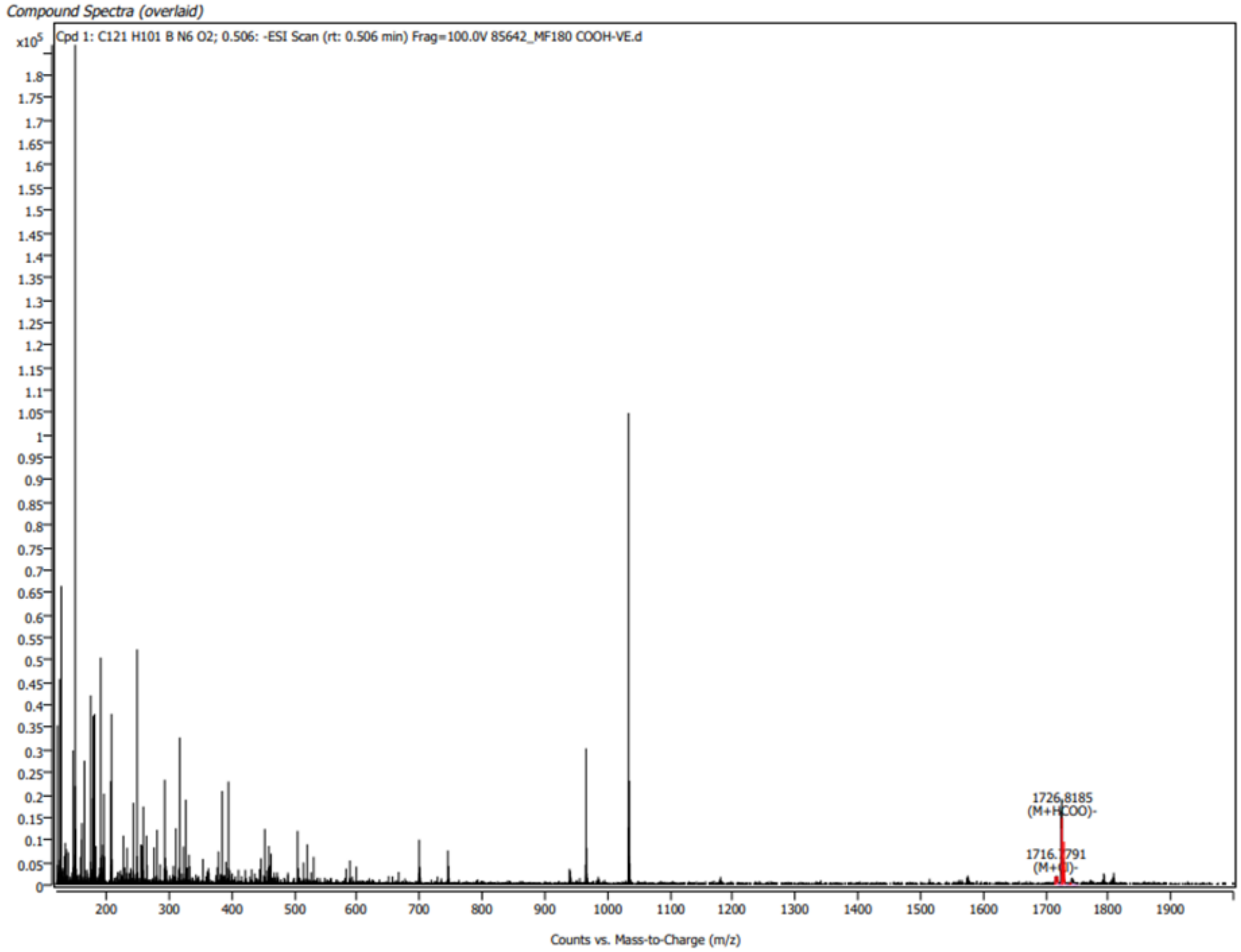

Figure **S34**. HRMS (ESI-MS) spectrum of **CzPBA-CzBN**.

Analysis Report

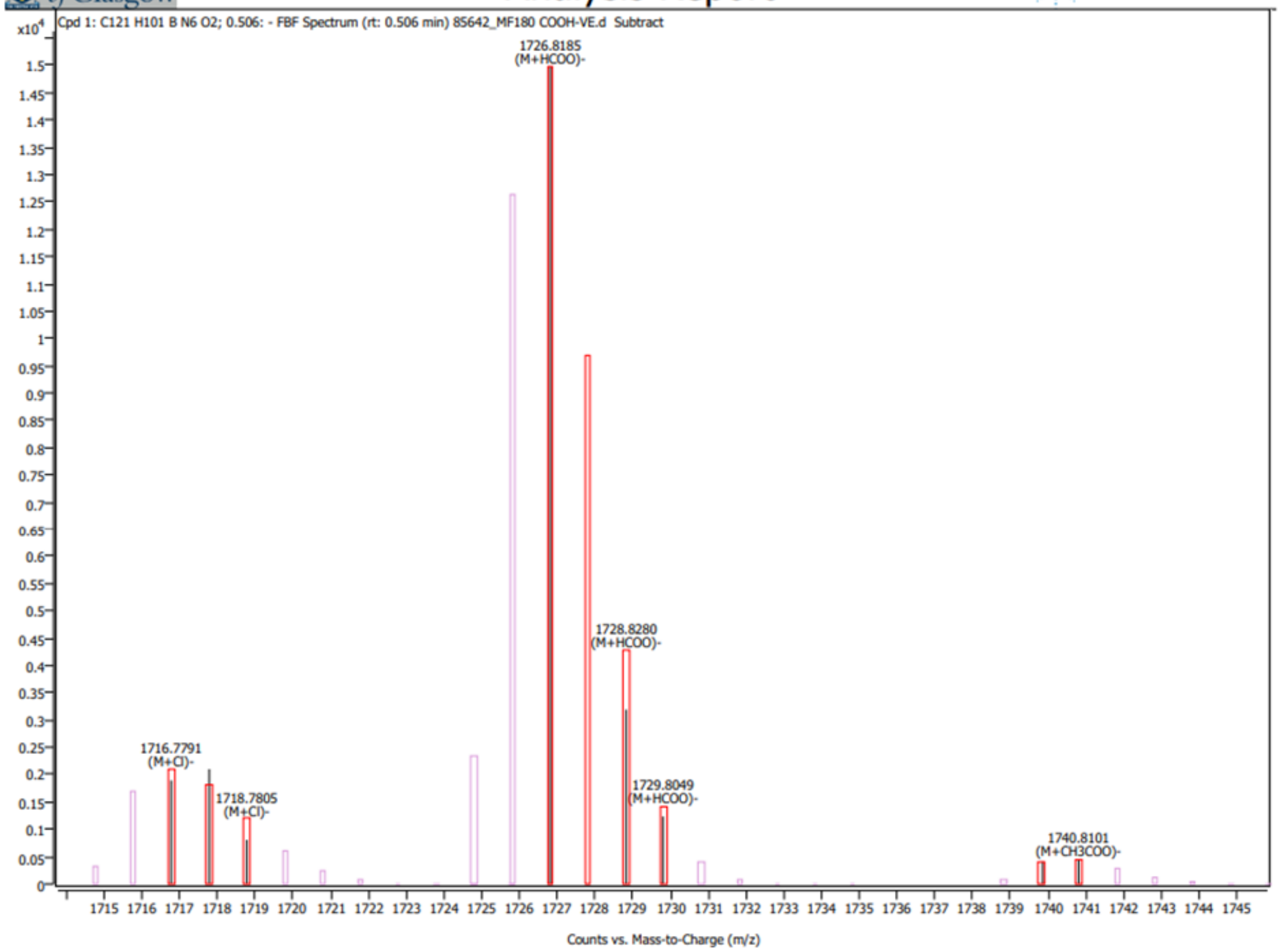

Spectrum Peaks

Ion Species	m/z	m/z (Calc)	Diff (ppm)	Abund	Height %	Height % (Calc)	Z
(M+Cl)-	1716.779126	1716.781396	-1.32	1895	90.20	100.00	1
(M+Cl)-	1717.786082	1717.782006	2.37	2101	100.00	86.57	1
(M+Cl)-	1718.780534	1718.782795	-1.32	806	38.37	56.74	1
(M+HCOO)-	1726.818488	1726.810382	4.69	14980	100.00	100.00	1
(M+HCOO)-	1728.828048	1728.816416	6.73	3190	21.29	28.49	1
(M+HCOO)-	1729.804878	1729.819531	-8.47	1235	8.24	9.53	1
(M+CH3COO)-	1739.855456	1739.823511	18.36	410	93.81	83.83	1
(M+CH3COO)-	1740.810087	1740.826040	-9.16	437	100.00	100.00	1
	112.985687			398015			

Figure **S35**. HRMS (ESI-MS) spectrum zoom in on compound peak of **CzPBA-CzBN**.

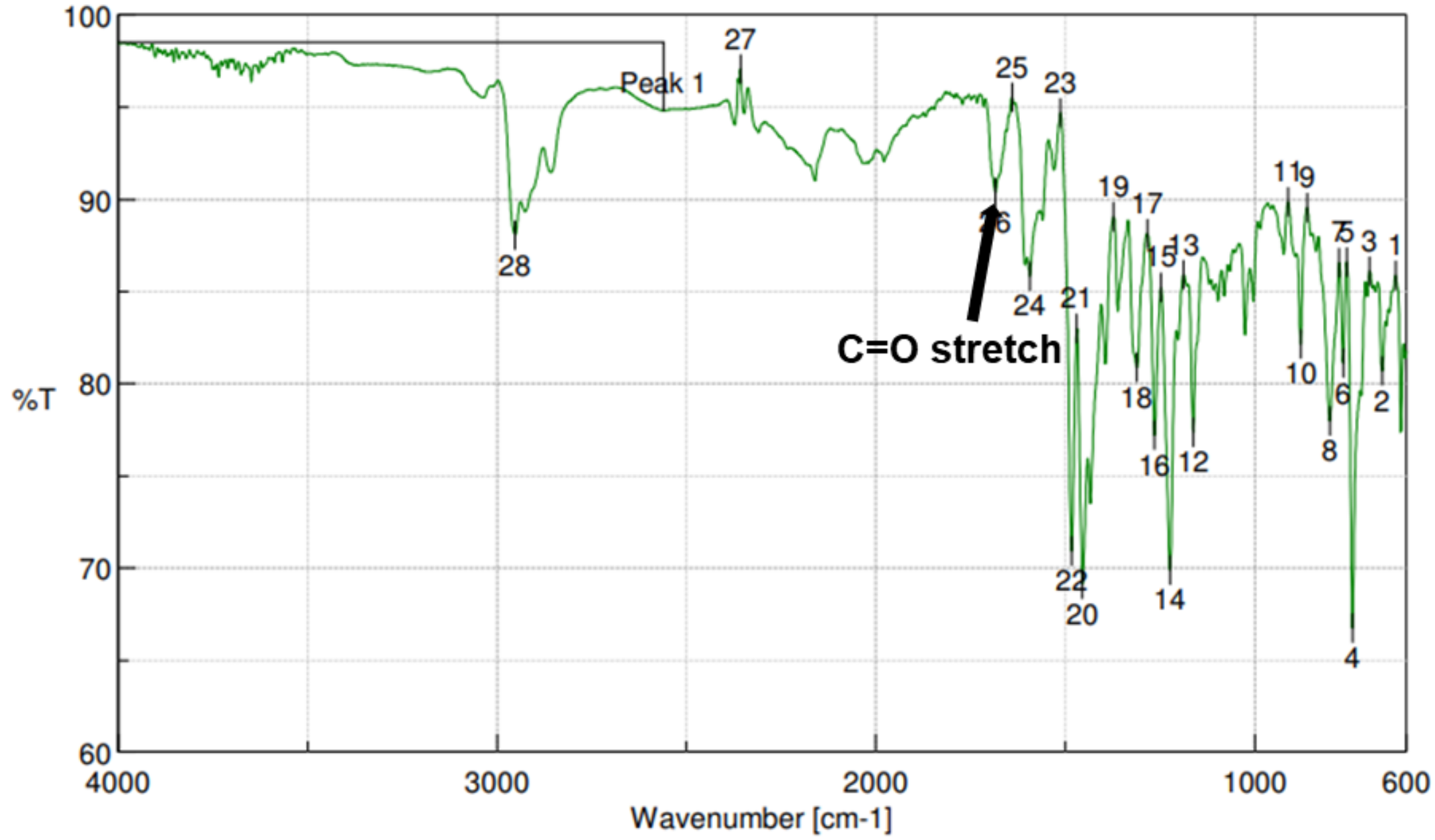

Results of Peak Height

	Peak	Base 1	Base 2	Height
Peak 1	2560	4000	-----	-3.73033

Results of Peak Find

No.	Position	Intensity	No.	Position	Intensity
1	628.68	85.8465	2	663.393	80.6807
3	696.177	86.0911	4	742.46	66.7178
5	756.923	86.567	6	766.566	81.1004
7	777.172	86.5207	8	802.242	77.9428
9	862.025	89.5136	10	878.417	82.1097
11	912.165	89.8315	12	1162.87	77.3495
13	1187.94	85.8852	14	1223.61	69.8425
15	1247.72	85.2111	16	1264.11	77.1742
17	1283.39	88.1073	18	1311.36	80.8475
19	1372.1	89.0493	20	1455.03	69.1077
21	1469.49	82.9786	22	1482.99	70.9066
23	1512.88	94.669	24	1593.88	85.8227
25	1640.16	95.528	26	1684.52	90.3383
27	2357.55	97.0307	28	2952.48	88.0509

[Comments]

Sample name	
Comment	
User	
Division	
Company	MCS Limited

[Measurement Information]

Model Name	FT/IR-6100typeA
Serial Number	A055661020
Measurement Date	05/02/2026 11:56
Light Source	Standard
Detector	TGS
Accumulation	64
Resolution	4 cm-1
Zero Filling	On
Apodization	Cosine
Gain	Auto (8)
Aperture	Auto (7.1 mm)
Scanning Speed	Auto (2 mm/sec)
Filter	Auto (10000 Hz)

Figure **S36**. FT-IR spectrum of **CzPBA-CzBN**.

HPLC Trace Report03Dec2025

<Sample Information>

Sample Name	: mf180.COOH		
Sample ID	:		
Method Filename	: 100% THF 20 mins 280nm - new-please use.lcm		
Batch Filename	: 02122025.lcb		
Vial #	: 1-2	Sample Type	: Unknown
Injection Volume	: 20 uL		
Date Acquired	: 02/12/2025 16:26:39	Acquired by	: System Administrator
Date Processed	: 02/12/2025 16:46:41	Processed by	: System Administrator

<Chromatogram>

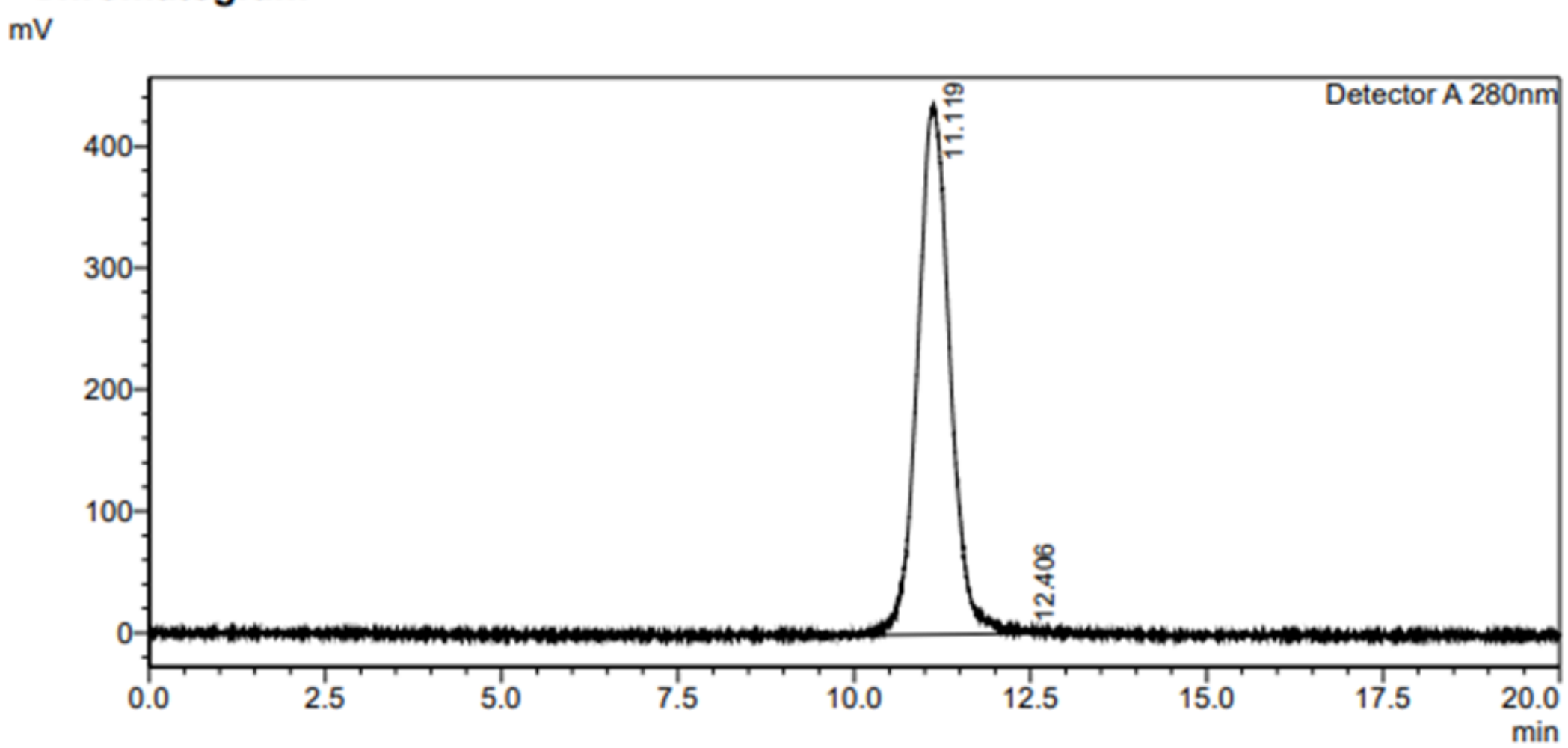

<Peak Table>

Detector A 280nm

Peak#	Ret. Time	Area	Height	Area%	Area/Height	Width at 5% Height
1	11.119	13855972	433261	99.340	31.981	1.095
2	12.406	92004	3316	0.660	27.743	--
Total		13947976	436578	100.000		

Figure **S37**. HPLC trace report of **CzPBA-CzBN**.

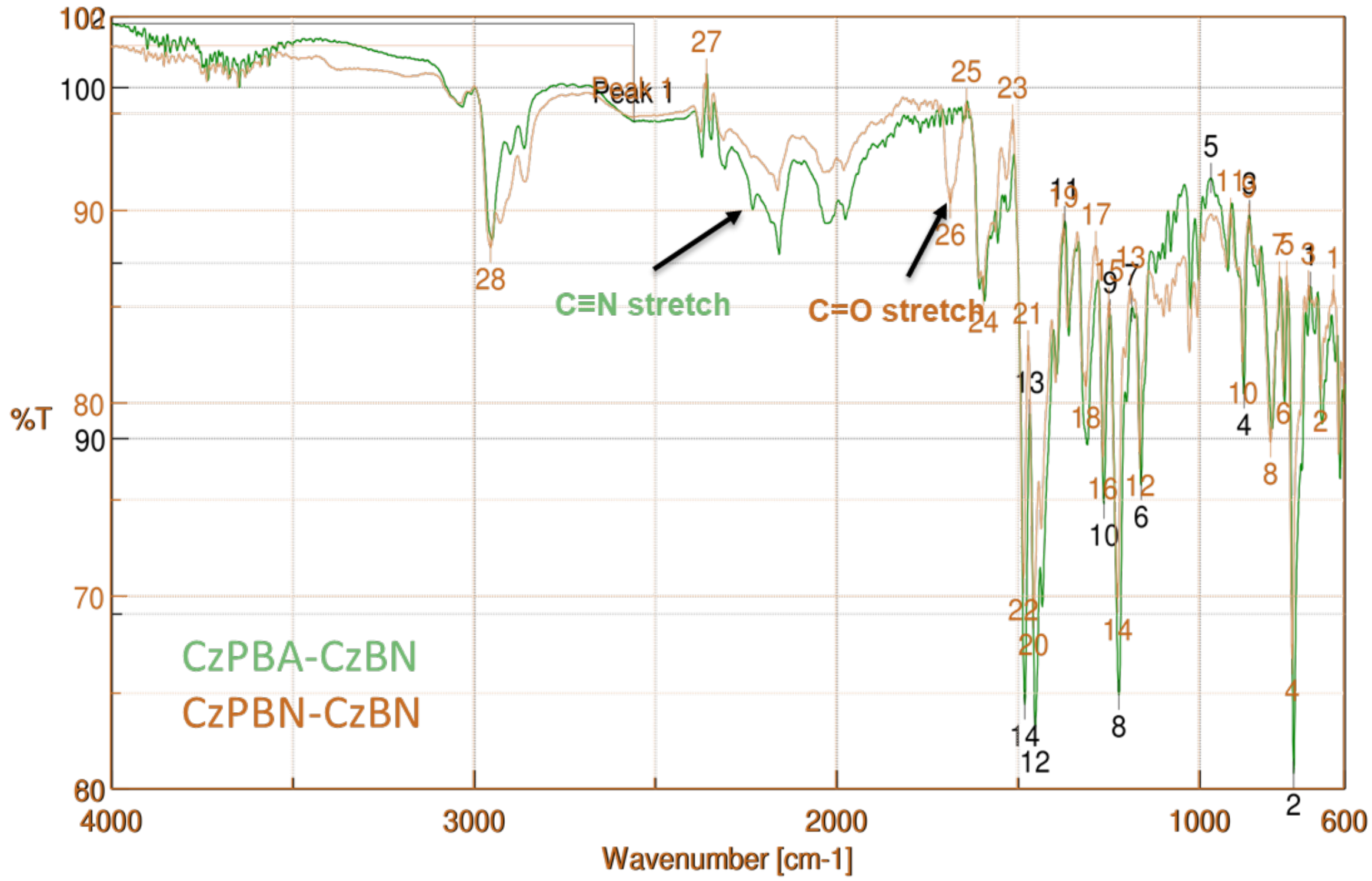

Figure **S38**. Overlapped IR-spectra of **CzPBN-CzBN** and **CzPBA-CzBN**.

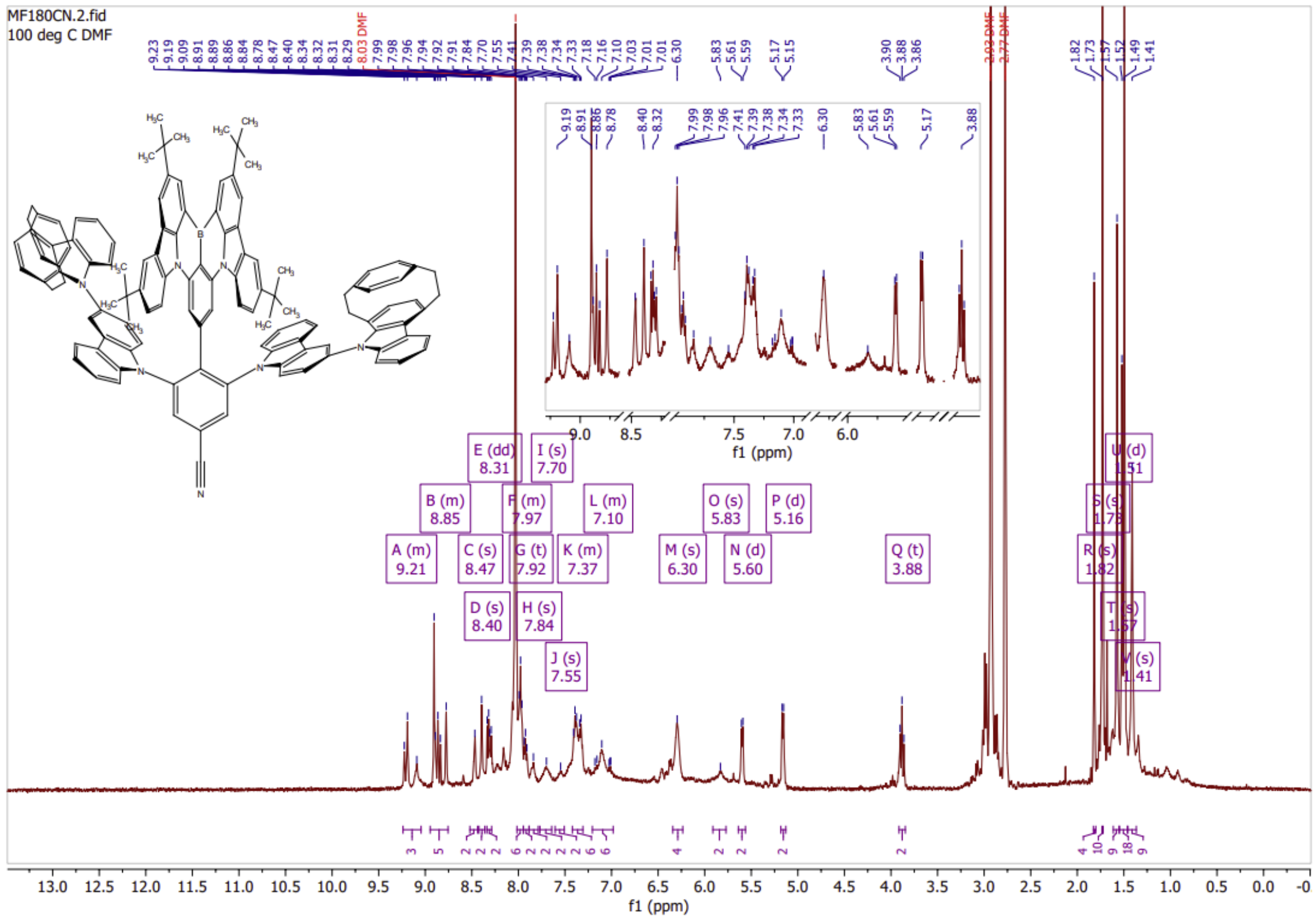

Figure **S39**. ^{1}H-NMR spectrum of ***(R*****p)-CzPBN-CzBN** in DMF-*d*7 at 100 °C.

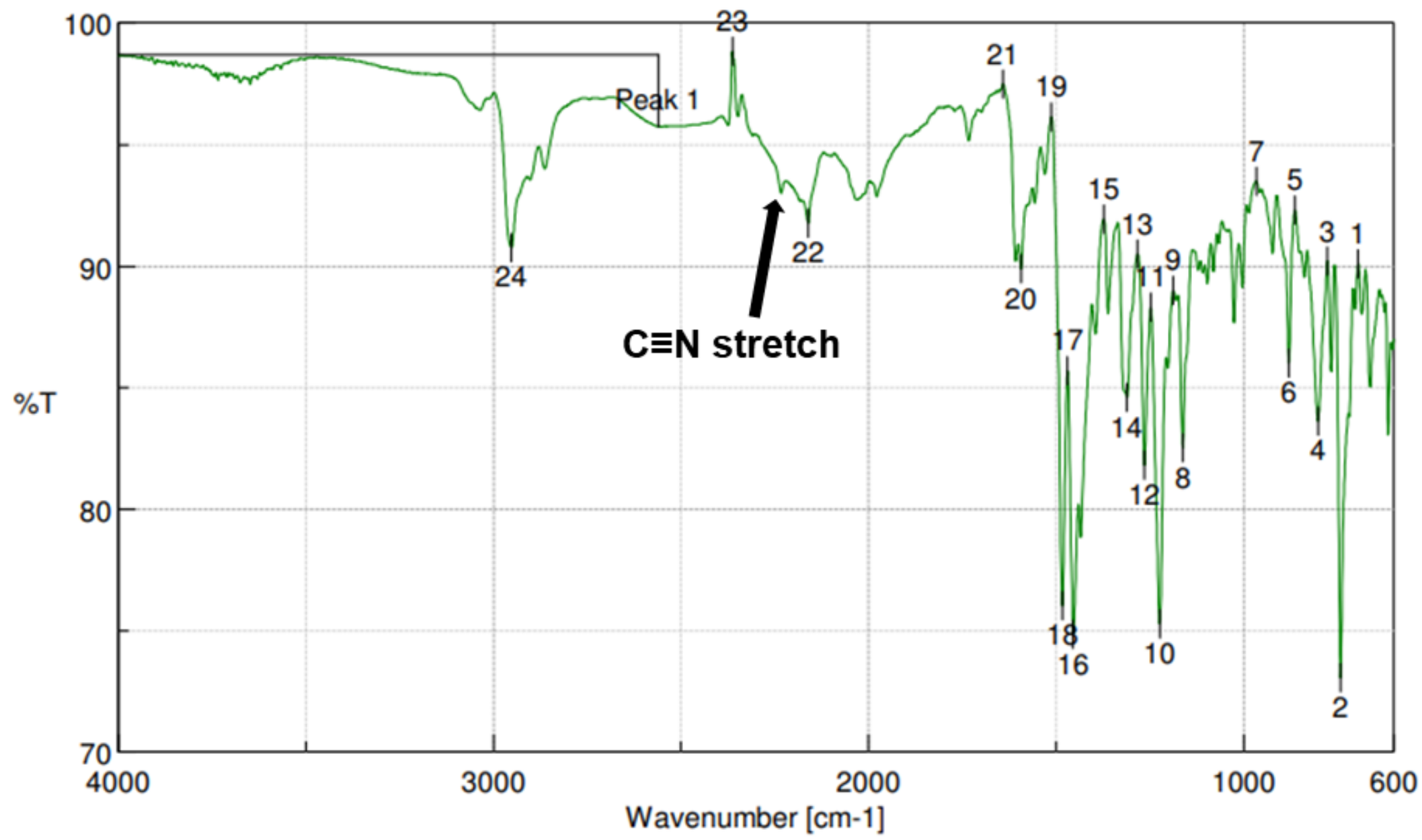

Results of Peak Height

	Peak	Base 1	Base 2	Height
Peak 1	2560	4000	-----	-2.95522

Results of Peak Find

No.	Position	Intensity	No.	Position	Intensity
1	695.212	90.0754	2	741.496	73.0352
3	777.172	90.2054	4	802.242	83.6065
5	863.953	92.2902	6	879.381	85.9911
7	966.162	93.4643	8	1162.87	82.5185
9	1187.94	88.9742	10	1223.61	75.2496
11	1247.72	88.2868	12	1264.11	81.7847
13	1282.43	90.4652	14	1311.36	84.5971
15	1372.1	91.9047	16	1455.03	74.8409
17	1469.49	85.6672	18	1482.99	76.0037
19	1512.88	96.1166	20	1593.88	89.8857
21	1641.13	97.4735	22	2160.85	91.7542
23	2363.34	98.8234	24	2952.48	90.7322

[Comments]

Sample name	
Comment	
User	
Division	
Company	MCS Limited

[Measurement Information]

Model Name	FT/IR-6100typeA
Serial Number	A055661020
Measurement Date	05/02/2026 11:33
Light Source	Standard
Detector	TGS
Accumulation	64
Resolution	4 cm-1
Zero Filling	On
Apodization	Cosine
Gain	Auto (8)
Aperture	Auto (7.1 mm)
Scanning Speed	Auto (2 mm/sec)
Filter	Auto (10000 Hz)

Figure **S40**. FT-IR spectrum of **(*R*$_p$)-CzPBN-CzBN**.

HPLC Trace Report05Dec2025

<Sample Information>

Sample Name	: mf180CN_R		
Sample ID	:		
Method Filename	: 100% THF 20 mins 280nm - new-please use.lcm		
Batch Filename	: enantiopure180.lcb		
Vial #	: 1-11	Sample Type	: Unknown
Injection Volume	: 15 uL		
Date Acquired	: 05/12/2025 13:43:00	Acquired by	: System Administrator
Date Processed	: 05/12/2025 14:03:03	Processed by	: System Administrator

<Chromatogram>

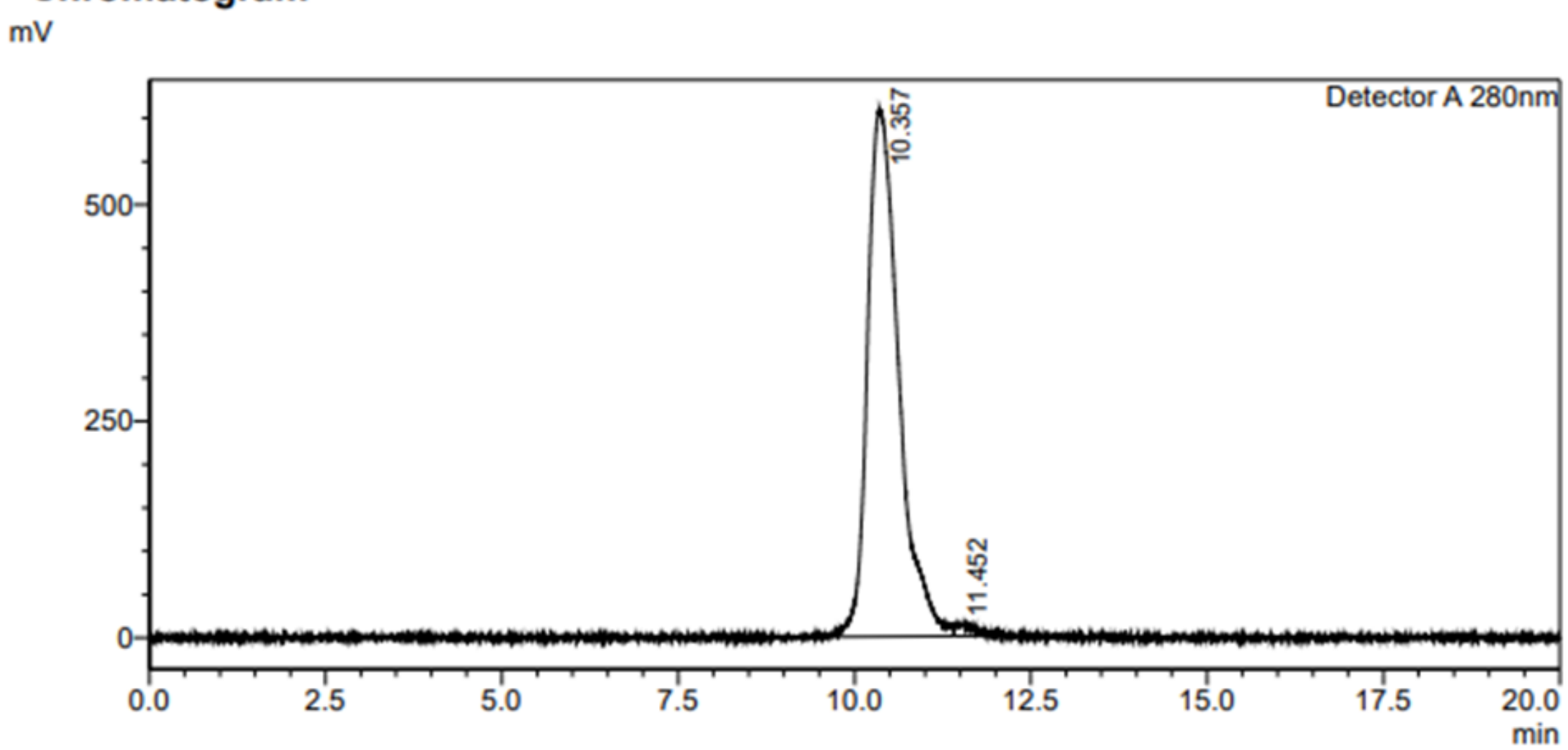

<Peak Table>

Detector A 280nm

Peak#	Ret. Time	Area	Height	Area%	Area/Height	Width at 5% Height
1	10.357	18756216	608666	98.509	30.815	1.126
2	11.452	283803	12986	1.491	21.854	--
Total		19040019	621652	100.000		

Figure **S41**. HPLC trace report of **(*R*$_p$)-CzPBN-CzBN**.

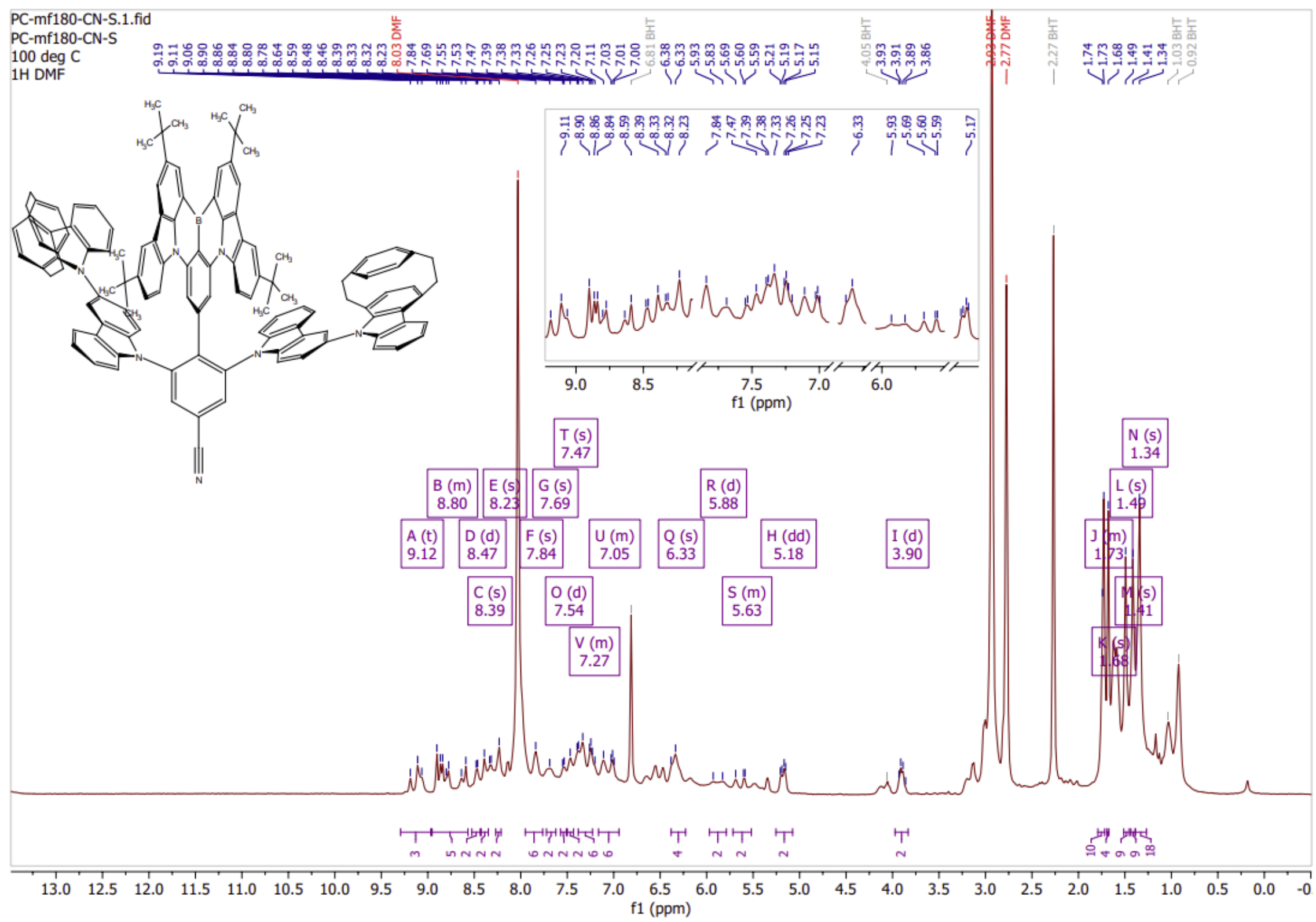

Figure **S42**. ^{1}H-NMR spectrum of **(S_p)-CzPBN-CzBN** in DMF-d_7 at 100 °C.

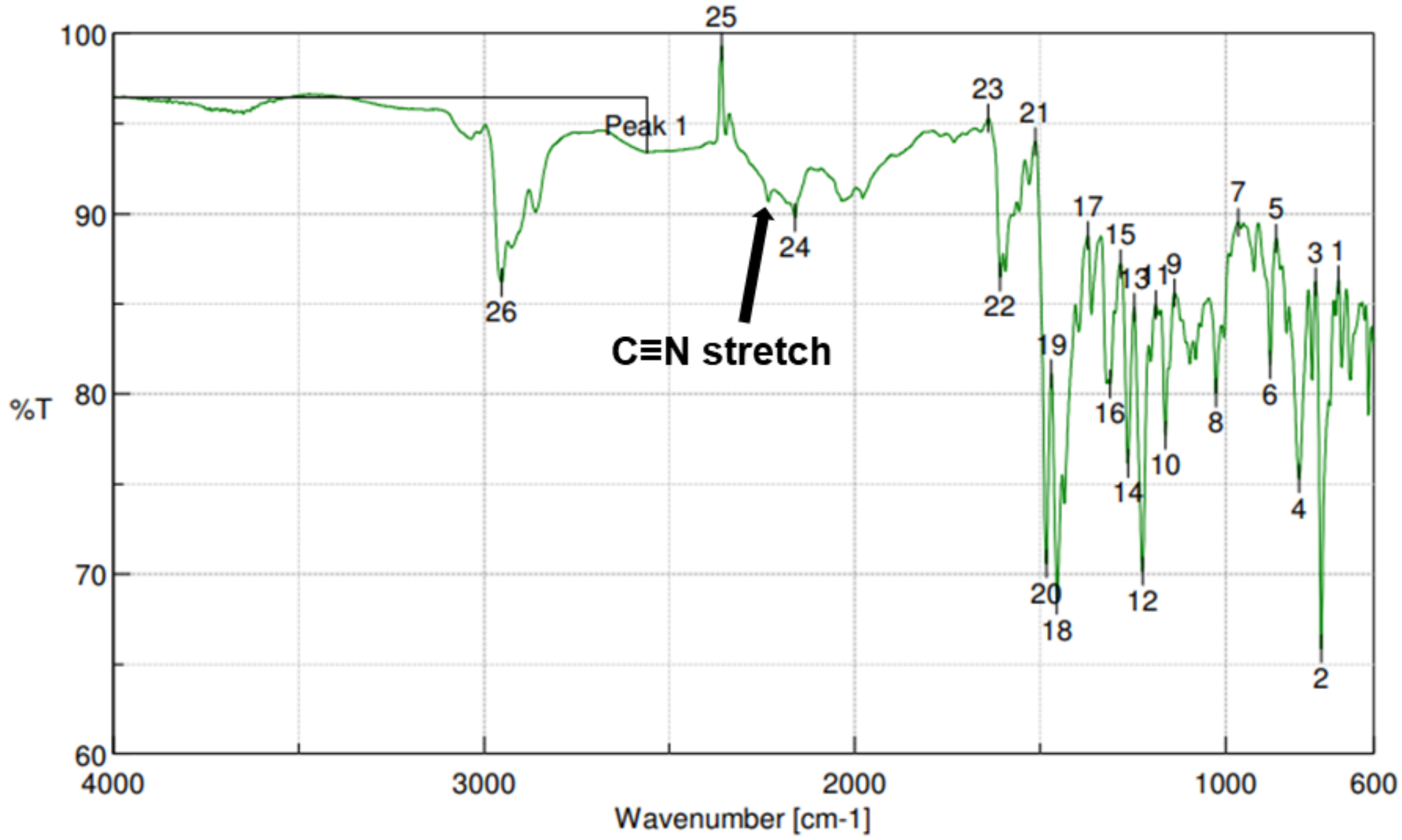

Results of Peak Height

	Peak	Base 1	Base 2	Height
Peak 1	2560	4000	-----	-3.09123

Results of Peak Find

No.	Position	Intensity	No.	Position	Intensity
1	695.212	86.3234	2	741.496	65.8166
3	756.923	86.1944	4	801.278	75.2786
5	862.989	88.6258	6	879.381	81.5787
7	965.198	89.5087	8	1024.98	80.0172
9	1137.8	85.5813	10	1162.87	77.6687
11	1188.9	84.9113	12	1223.61	70.1297
13	1246.75	84.7872	14	1263.15	76.1025
15	1282.43	87.2004	16	1311.36	80.5339
17	1371.14	88.768	18	1455.03	68.53
19	1469.49	81.0986	20	1483.96	70.5621
21	1513.85	93.9716	22	1607.38	86.4479
23	1639.2	95.2893	24	2160.85	89.7581
25	2359.48	99.2775	26	2952.48	86.1616

[Comments]

Sample name	
Comment	
User	
Division	
Company	MCS Limited

[Measurement Information]

Model Name	FT/IR-6100typeA
Serial Number	A055661020
Measurement Date	05/02/2026 11:29
Light Source	Standard
Detector	TGS
Accumulation	64
Resolution	4 cm-1
Zero Filling	On
Apodization	Cosine
Gain	Auto (8)
Aperture	Auto (7.1 mm)
Scanning Speed	Auto (2 mm/sec)
Filter	Auto (10000 Hz)

Figure **S43**. FT-IR spectrum of **(*S*$_{p}$)-CzPBN-CzBN**.

HPLC Trace Report05Dec2025

<Sample Information>

Sample Name	: mf180CN_S		
Sample ID	:		
Method Filename	: 100% THF 20 mins 280nm - new-please use.lcm		
Batch Filename	: enantiopure180.lcb		
Vial #	: 1-10	Sample Type	: Unknown
Injection Volume	: 15 uL		
Date Acquired	: 05/12/2025 13:05:24	Acquired by	: System Administrator
Date Processed	: 05/12/2025 13:25:27	Processed by	: System Administrator

<Chromatogram>

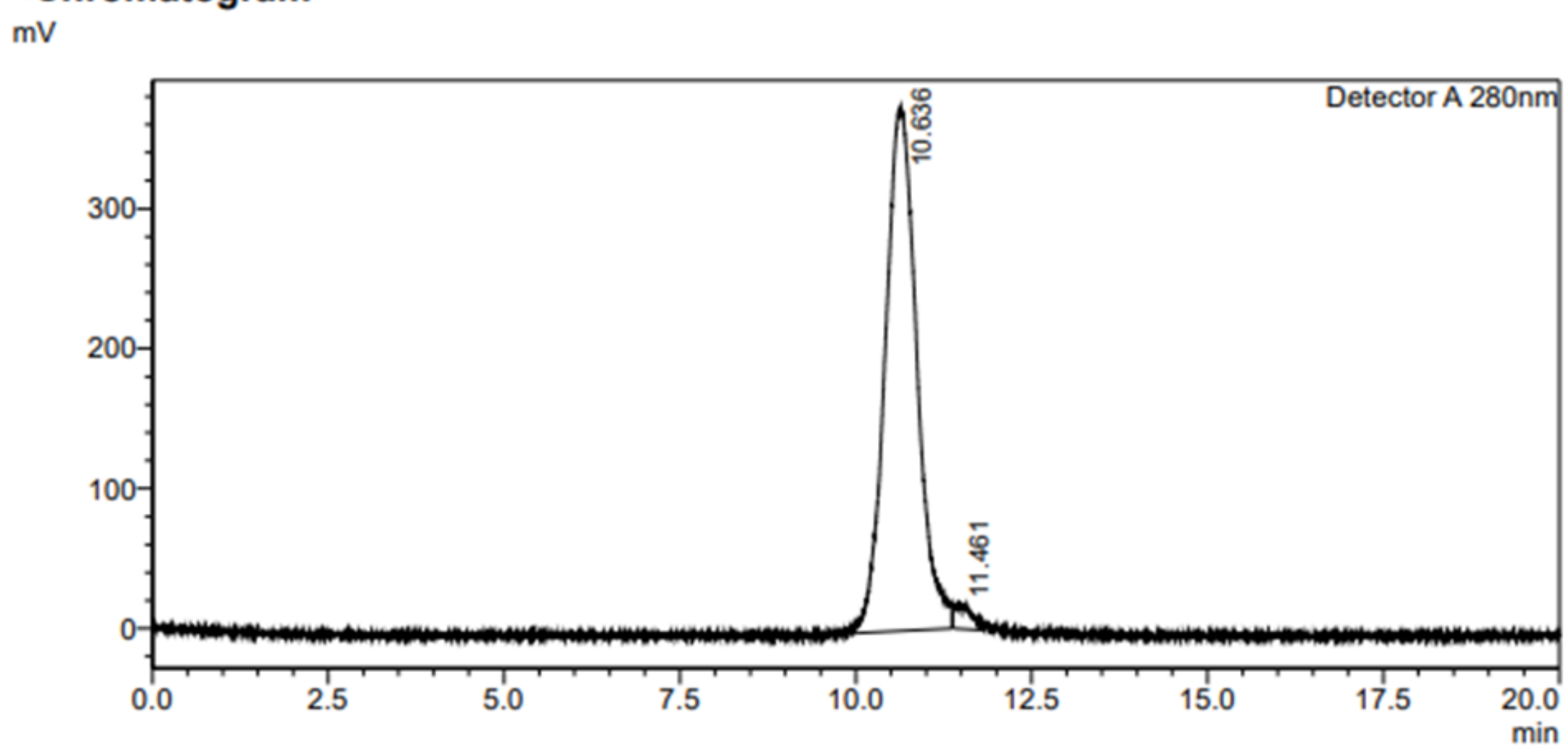

<Peak Table>

Detector A 280nm

Peak#	Ret. Time	Area	Height	Area%	Area/Height	Width at 5% Height
1	10.636	11703188	372118	97.396	31.450	1.157
2	11.461	312869	16805	2.604	18.617	--
Total		12016057	388923	100.000		

Figure **S44**. HPLC trace report of **(*S*$_p$)-CzPBN-CzBN**.

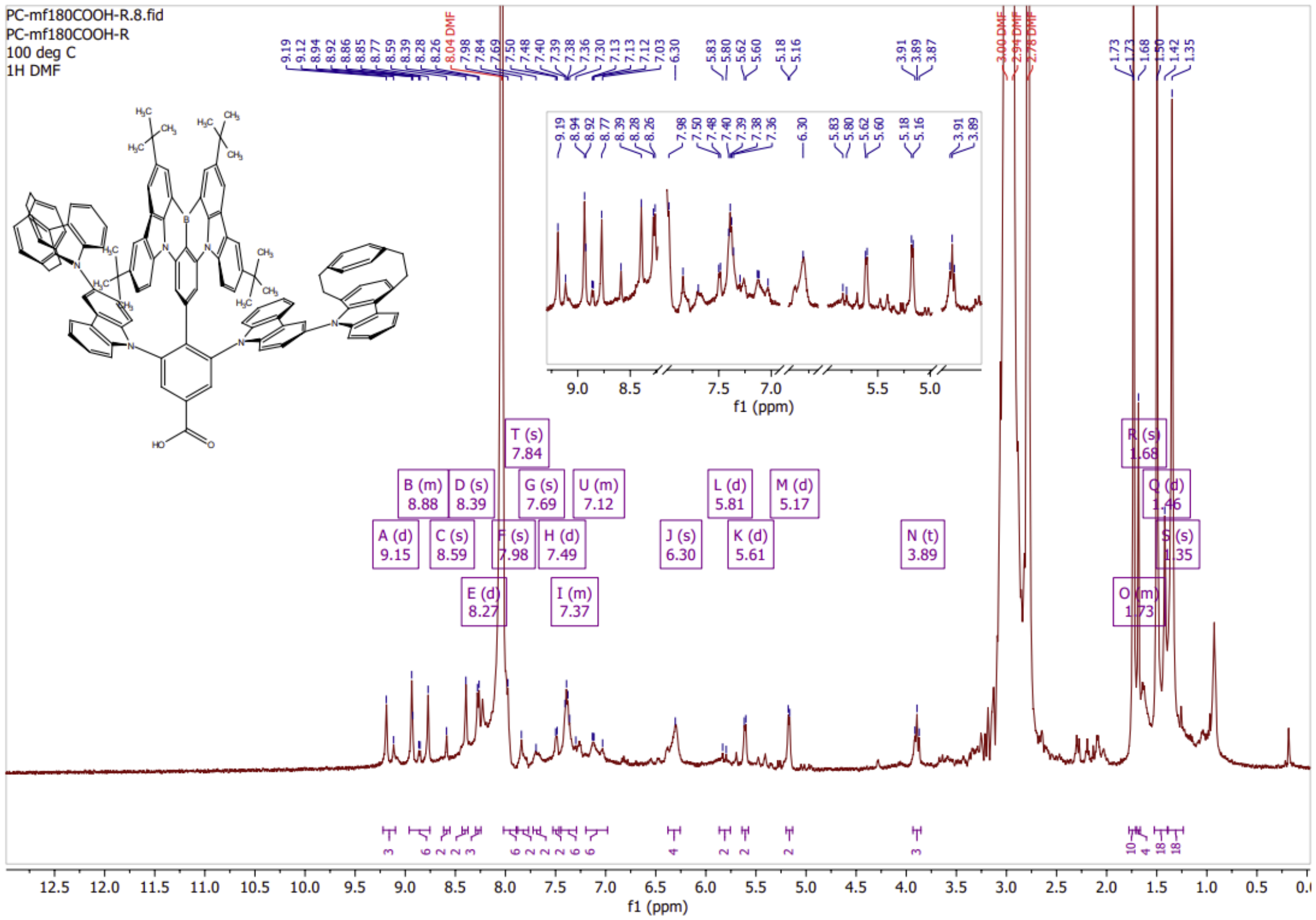

Figure **S45**. ^{1}H-NMR spectrum of **(*R*$_p$)-CzPBA-CzBN** in DMF-d_7 at 100 °C.

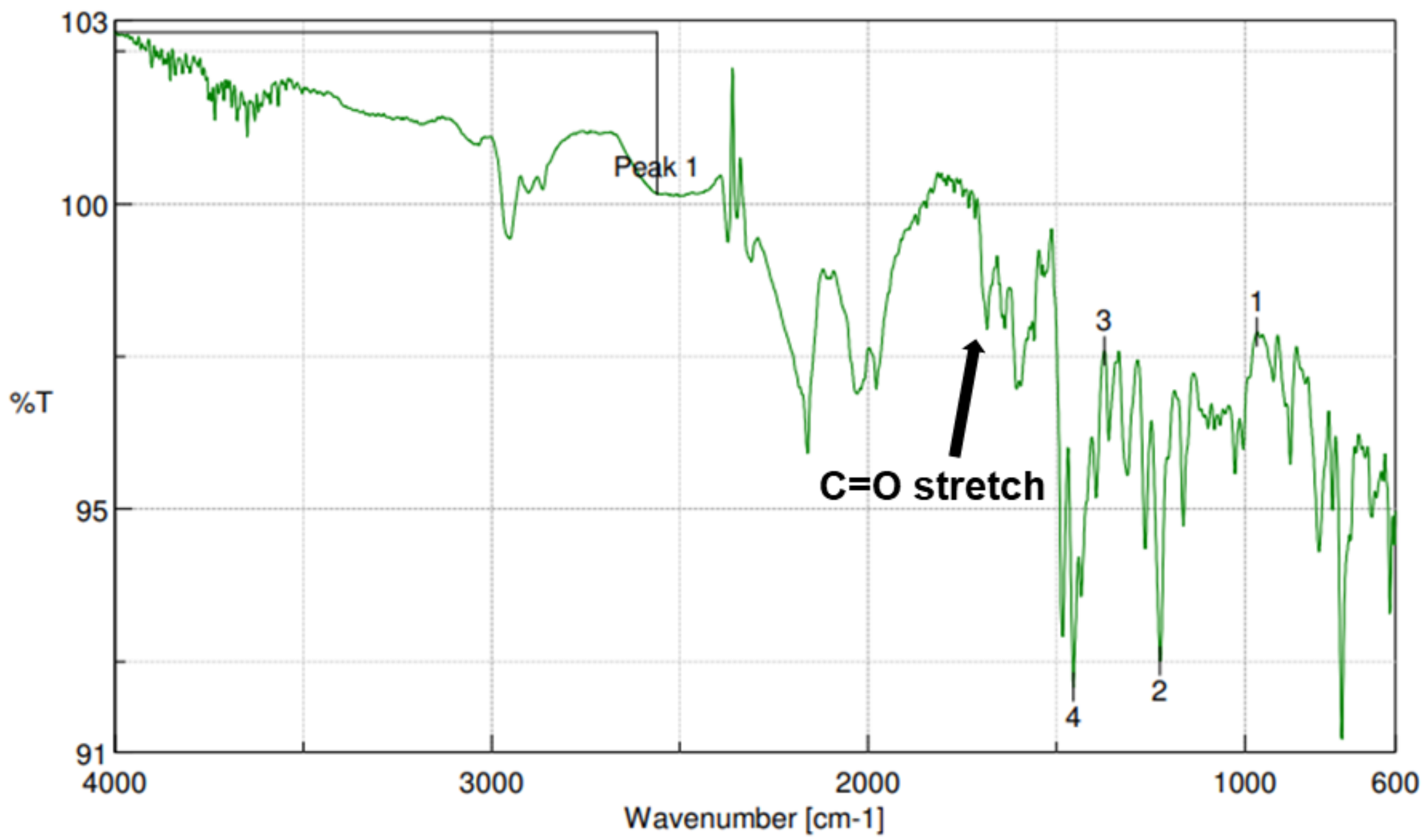

Results of Peak Height

	Peak	Base 1	Base 2	Height
Peak 1	2560	4000	-----	-2.65112

Results of Peak Find

No.	Position	Intensity	No.	Position	Intensity
1	967.126	97.8936	2	1224.58	92.495
3	1372.1	97.5837	4	1455.99	92.0713

[Comments]

Sample name	
Comment	
User	
Division	
Company	MCS Limited

[Measurement Information]

Model Name	FT/IR-6100typeA
Serial Number	A055661020

Measurement Date 05/02/2026 11:44

Light Source	Standard
Detector	TGS
Accumulation	64
Resolution	4 cm-1
Zero Filling	On
Apodization	Cosine
Gain	Auto (4)
Aperture	Auto (7.1 mm)
Scanning Speed	Auto (2 mm/sec)
Filter	Auto (10000 Hz)

Figure **S46**. FT-IR spectrum of **(*R*$_p$)-CzPBA-CzBN**.

HPLC Trace Report08Jan2026

<Sample Information>

Sample Name	: mf180.COOH_R		
Sample ID	:		
Method Filename	: 100% THF 20 mins 280nm - new-please use.lcm		
Batch Filename	: enantiopure180COOH.lcb		
Vial #	: 1-3	Sample Type	: Unknown
Injection Volume	: 10 uL		
Date Acquired	: 10/12/2025 15:43:56	Acquired by	: System Administrator
Date Processed	: 10/12/2025 16:03:57	Processed by	: System Administrator

<Chromatogram>

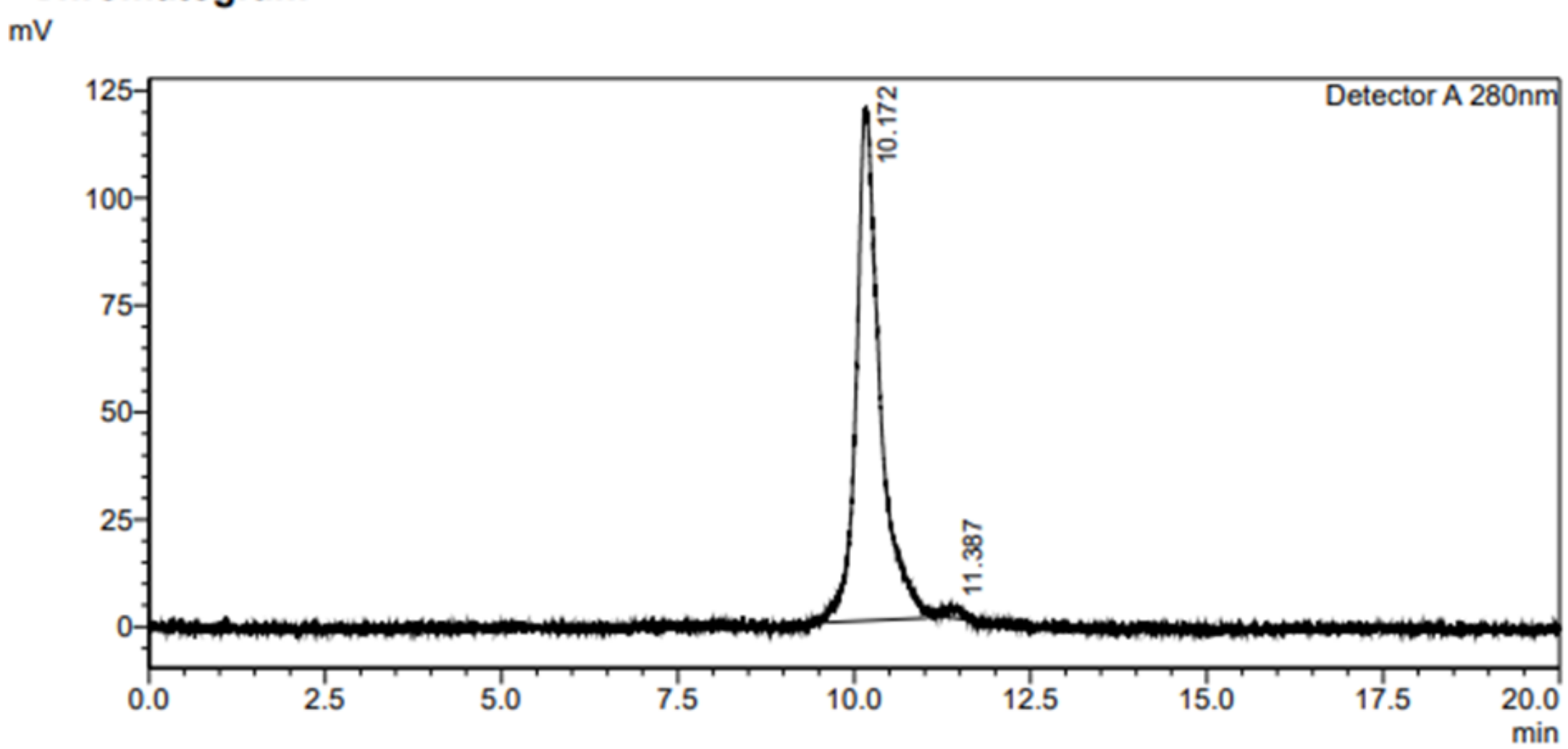

<Peak Table>

Detector A 280nm

Peak#	Ret. Time	Area	Height	Area%	Area/Height	Width at 5% Height
1	10.172	2799601	119197	98.633	23.487	1.028
2	11.387	38803	2357	1.367	16.465	0.405
Total		2838404	121553	100.000		

Figure **S47**. HPLC trace report of **(*R*$_p$)-CzPBA-CzBN**.

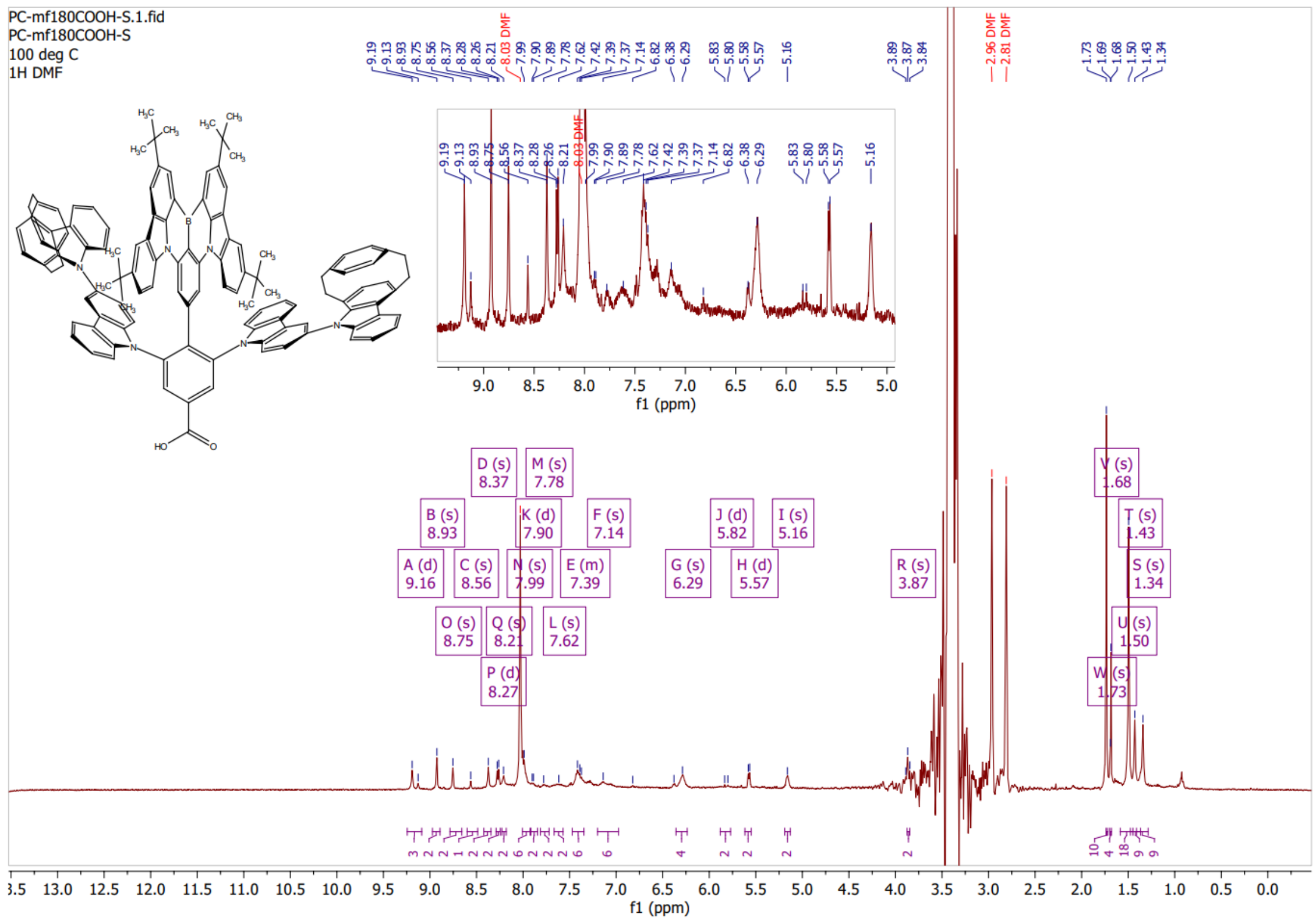

Figure **S48**. ^{1}H-NMR spectrum of **(S_p)-CzPBA-CzBN** in DMF-d_7 at 100 °C.

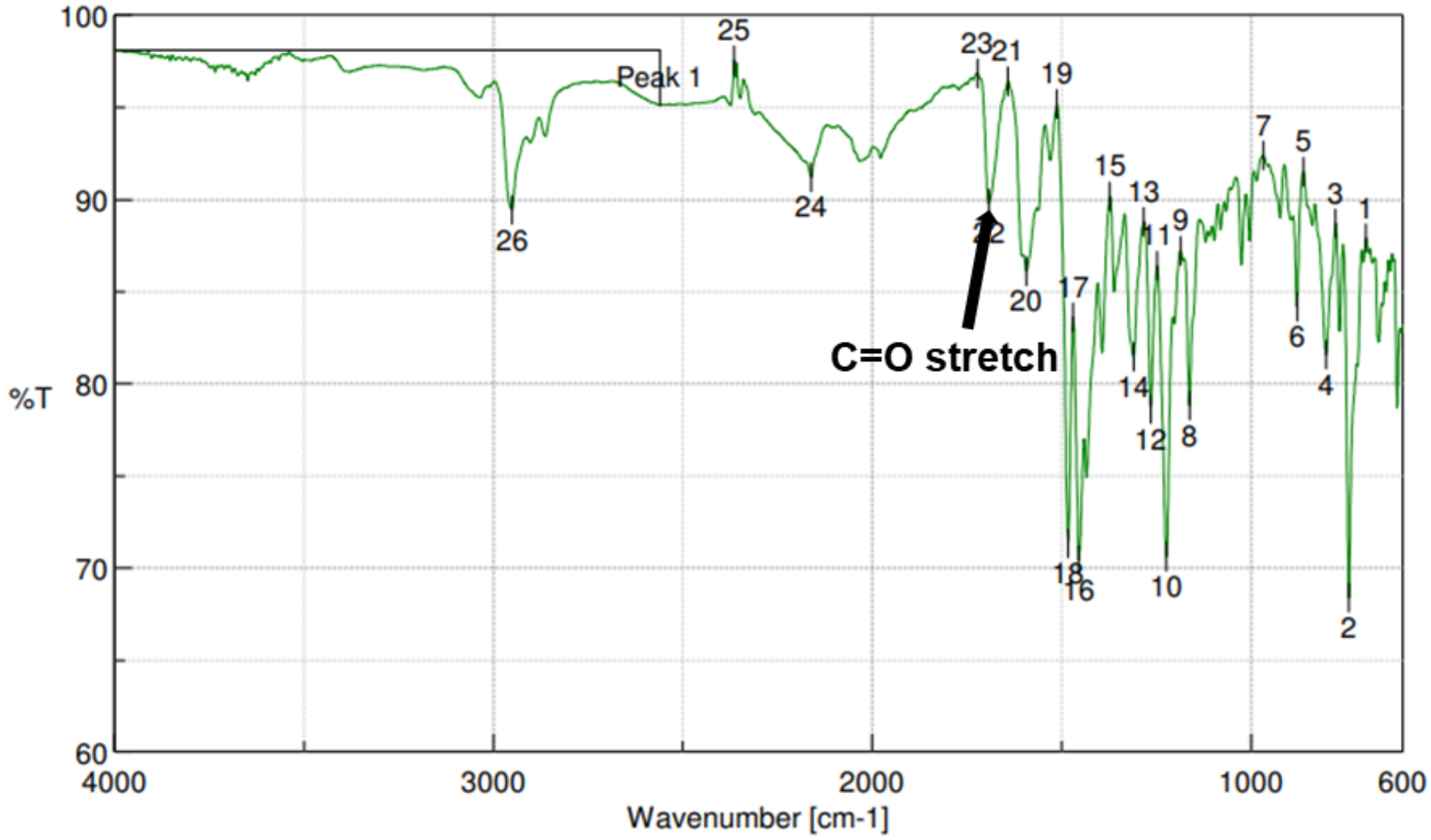

Results of Peak Height

	Peak	Base 1	Base 2	Height
Peak 1	2560	4000	-----	-2.95941

Results of Peak Find

No.	Position	Intensity	No.	Position	Intensity
1	696.177	87.8831	2	741.496	68.3766
3	777.172	88.6939	4	802.242	81.5372
5	862.025	91.4944	6	878.417	84.162
7	968.09	92.3513	8	1162.87	78.799
9	1186.01	87.189	10	1223.61	70.5995
11	1248.68	86.3818	12	1265.07	78.6089
13	1282.43	88.7613	14	1310.39	81.4499
15	1372.1	90.1502	16	1455.03	70.3914
17	1469.49	83.5696	18	1482.99	71.3322
19	1512.88	95.1612	20	1592.91	86.0727
21	1641.13	96.3971	22	1692.23	89.7788
23	1722.12	96.8243	24	2160.85	91.1694
25	2364.3	97.5554	26	2951.52	89.4284

[Comments]

Sample name	
Comment	
User	
Division	
Company	MCS Limited

[Measurement Information]

Model Name	FT/IR-6100typeA
Serial Number	A055661020
Measurement Date	05/02/2026 11:37
Light Source	Standard
Detector	TGS
Accumulation	64
Resolution	4 cm-1
Zero Filling	On
Apodization	Cosine
Gain	Auto (8)
Aperture	Auto (7.1 mm)
Scanning Speed	Auto (2 mm/sec)
Filter	Auto (10000 Hz)

Figure **S49**. FT-IR spectrum of ***(S*$_p$)-CzPBA-CzBN**.

HPLC Trace Report08Jan2026

<Sample Information>

Sample Name : mf180.COOH_S
Sample ID :
Method Filename : 100% THF 20 mins 280nm - new-please use.lcm
Batch Filename : enantiopure180COOH.lcb
Vial # : 1-2 Sample Type : Unknown
Injection Volume : 50 uL
Date Acquired : 10/12/2025 16:24:57 Acquired by : System Administrator
Date Processed : 10/12/2025 16:44:59 Processed by : System Administrator

<Chromatogram>

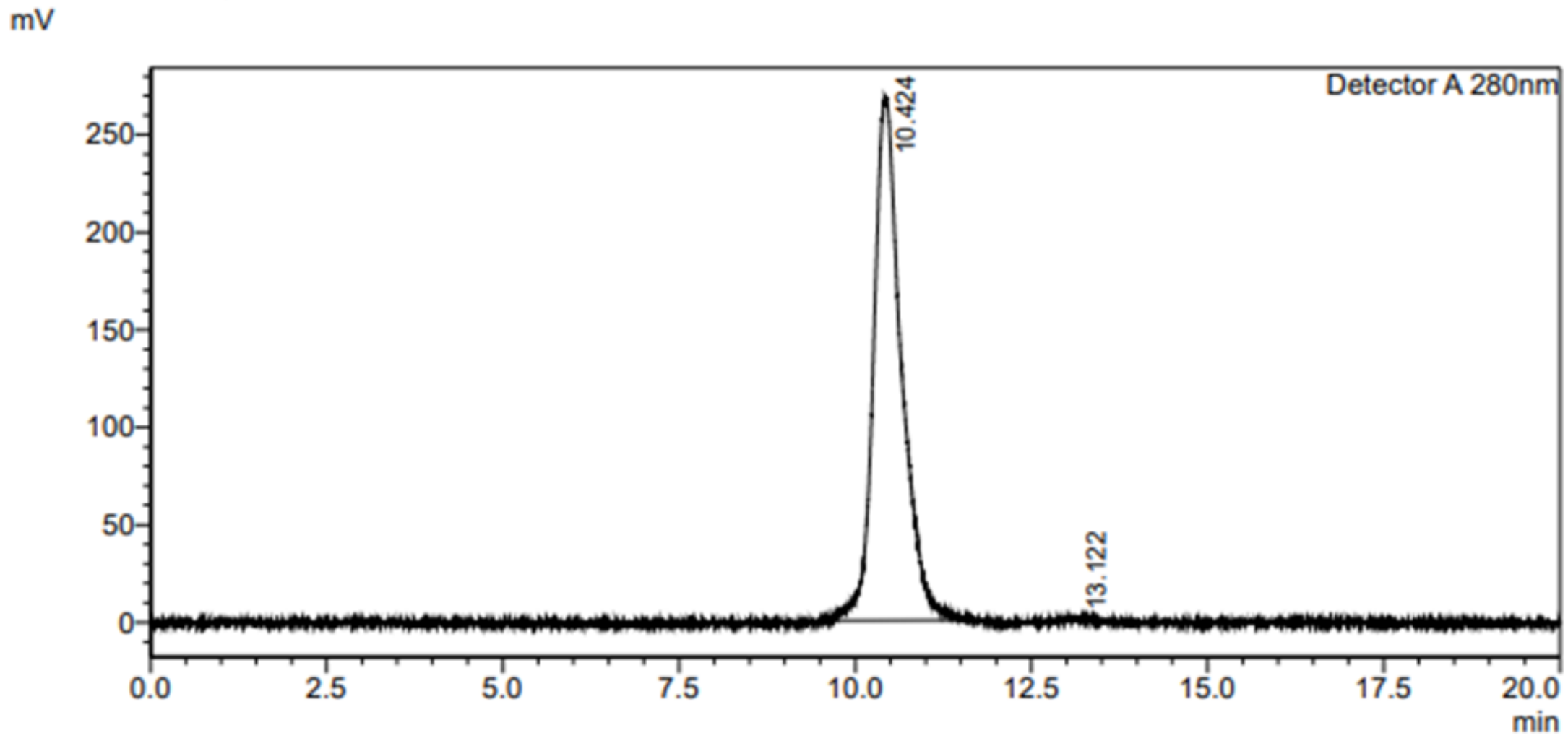

<Peak Table>

Detector A 280nm

Peak#	Ret. Time	Area	Height	Area%	Area/Height	Width at 5% Height
1	10.424	7390206	268013	99.542	27.574	0.994
2	13.122	34037	1866	0.458	18.245	0.119
Total		7424243	269879	100.000		

Figure **S50**. HPLC trace report of **(*S*$_p$)-CzPBA-CzBN**.

References

[1] M. Y. Wong, G. J. Hedley, G. Xie, L. S. Kölln, I. D. W. Samuel, A. Pertegás, H. J. Bolink, E. Zysman-Colman, *Chemistry of Materials* **2015**, 27, 6535.

[2] N. Sharma, E. Spuling, Cornelia M. Mattern, W. Li, O. Fuhr, Y. Tsuchiya, C. Adachi, S. Bräse, I. D. W. Samuel, E. Zysman-Colman, *Chemical Science* **2019**, 10, 6689.

[3] S. Wu, D. Chen, X.-H. Zhang, D. Sun, E. Zysman-Colman, *Advanced Materials* **2025**, 37, 2415289.

[4] M. J. Frisch, G. W. Trucks, H. B. Schlegel, G. E. Scuseria, M. A. Robb, J. R. Cheeseman, G. Scalmani, V. Barone, G. A. Petersson, H. Nakatsuji, X. Li, M. Caricato, A. V. Marenich, J. Bloino, B. G. Janesko, R. Gomperts, B. Mennucci, H. P. Hratchian, J. V. Ortiz, A. F. Izmaylov, J. L. Sonnenberg, Williams, F. Ding, F. Lipparini, F. Egidi, J. Goings, B. Peng, A. Petrone, T. Henderson, D. Ranasinghe, V. G. Zakrzewski, J. Gao, N. Rega, G. Zheng, W. Liang, M. Hada, M. Ehara, K. Toyota, R. Fukuda, J. Hasegawa, M. Ishida, T. Nakajima, Y. Honda, O. Kitao, H. Nakai, T. Vreven, K. Throssell, J. A. Montgomery Jr., J. E. Peralta, F. Ogliaro, M. J. Bearpark, J. J. Heyd, E. N. Brothers, K. N. Kudin, V. N. Staroverov, T. A. Keith, R. Kobayashi, J. Normand, K. Raghavachari, A. P. Rendell, J. C. Burant, S. S. Iyengar, J. Tomasi, M. Cossi, J. M. Millam, M. Klene, C. Adamo, R. Cammi, J. W. Ochterski, R. L. Martin, K. Morokuma, O. Farkas, J. B. Foresman, D. J. Fox, Wallingford, CT 2016.

[5] C. Adamo, V. Barone, *J. Chem. Phys.* **1999**, 110, 6158.

[6] G. A. Petersson, M. A. Al-Laham, *J. Chem. Phys.* **1991**, 94, 6081.

[7] S. Grimme, J. Antony, S. Ehrlich, H. Krieg, *J. Chem. Phys.* **2010**, 132, 154104.

[8] R. Dennington, T. A. Keith, J. M. Millam, Semichem Inc., Shawnee Mission, KS, 2016.

[9] S. Hirata, M. Head-Gordon, *Chem. Phys. Lett.* **1999**, 314, 291.

[10] X. Gao, S. Bai, D. Fazzi, T. Niehaus, M. Barbatti, W. Thiel, *J. Chem. Theory Comput.* **2017**, 13, 515.

[11] O. S. Lee, E. Zysman-Colman, *Digichem (version 6) InSilico Computing*, St Andrews, Scotland, **2024**.

[12] O. Lee, M. Gather, E. Zysman-Colman, *Digital Discovery* **2024**, 3, 1695.

[13] N. M. O'Boyle, A. L. Tenderholt, K. M. Langner, *J. Comput. Chem.* **2008**, 29, 839.

[14] W. Humphrey, A. Dalke, K. Schulten, *Journal of Molecular Graphics* **1996**, 14, 33.

[15] J. E. Stone, in *Faculty of the Graduate School of the University of Missouri - Rolla*, Vol. Master of Science in Computer Science, University of Missouri - Rolla, 1998.

[16] J. D. Hunter, *Computing in Science & Engineering* **2007**, 9, 90.

[17] N. M. O'Boyle, M. Banck, C. A. James, C. Morley, T. Vandermeersch, G. R. Hutchison, *Journal of Cheminformatics* **2011**, 3, 1.

[18] N. M. O'Boyle, G. R. Hutchison, *Chemistry Central Journal* **2008**, 2, 24.

[19] G. A. Crosby, J. N. Demas, *J. Phys. Chem.* **1971**, 75, 991.

[20] W. H. Melhuish, *J. Phys. Chem.* **1961**, 65, 229.

[21] V. V. Pavlishchuk, A. W. Addison, *Inorg. Chim. Acta* **2000**, 298, 97.

[22] Y. Tsuchiya, S. Diesing, F. Bencheikh, Y. Wada, P. L. Dos Santos, H. Kaji, E. Zysman-Colman, I. D. W. Samuel, C. Adachi, *J. Phys. Chem. A* **2021**, 125, 8074.
